# Solid‐State Quantum Coherence From a High‐Spin Donor–Acceptor Conjugated Polymer

**DOI:** 10.1002/adma.202501884

**Published:** 2025-09-06

**Authors:** Alexander J. Bushnell, Tanya A. Balandin, Paramasivam Mahalingam, Chih‐Ting Liu, Michael K. Bowman, Jason D. Azoulay

**Affiliations:** ^1^ School of Chemistry and Biochemistry School of Materials Science and Engineering Center for Organic Photonics and Electronics Georgia Institute of Technology Atlanta GA 30332 USA; ^2^ Department of Chemistry and Biochemistry The University of Alabama Tuscaloosa AL 35487‐0336 USA

**Keywords:** conjugated polymers, organic semiconductors, quantum materials, triplets

## Abstract

Molecular spin systems that can be chemically tuned, coherently controlled, and readily integrated within devices remain central to the realization of emerging quantum technologies. Organic high‐spin materials are prime candidates owing to their similarity in electronic structure to leading solid‐state defect‐based systems, light element composition, and the potential for entanglement and qubit operations mediated through spin‐spin exchange. However, the inherent instability of these species precludes their rational design, development, and application. Here, the first example of an organic high‐spin qubit based on a conjugated polymer semiconductor comprised of alternating dithienosilole and thiadiazoloquinoxaline heterocycles is demonstrated. It is shown that electron spins within the macromolecule demonstrate high‐fidelity coherent control of the superposition state with room temperature coherence and solid‐state relaxation times that are competitive with or exceed other synthetic molecular qubits. These attributes, along with robust stability, chemical tunability, rich interrelated optoelectronic functionalities, and solution processability, offer a fundamentally new approach to integrating quantum phenomena within functional device platforms.

## Graphical abstract

High‐spin donor–acceptor conjugated polymers demonstrate newly realized quantum functionalities such as long solid‐state spin relaxation times, coherent control of the superposition state, and single qubit operations. These attributes coupled with high chemical tunability, robust stability, interrelated optoelectronic properties, and solution processability offer a fundamentally new approach to readily integrating quantum phenomena within functional device platforms.

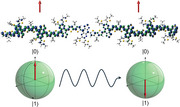

## 1 Introduction

Discovering pathways to understand and control the quantum properties of matter remains central to the realization of a new generation of quantum technologies spanning computation, information science, communications, energy, sensing, and many others.^[^ ^1^, ^2^, ^3^, ^4^, ^5^, ^6^ ^]^ Important materials derive their functionality from defect engineering in solid‐state materials and semiconductors^[^ ^7^, ^8^, ^9^, ^10^ ^]^ such as nitrogen‐vacancy (NV^−^) centers in diamond,^[^ ^11^, ^12^, ^13^, ^14^ ^]^ donors in silicon,^[^ ^15^, ^16^ ^]^ and defects in silicon carbide (SiC).^[^ ^17^, ^18^ ^]^ These paramagnetic defects define the core quantum unit (i.e., the “qubit”)—a structure with discrete energy levels and associated quantum states capable of encoding information. Despite the maturity and performance of these materials,^[^ ^8^, ^9^, ^10^ ^]^ emerging technologies require an extended range of functionality that is not available from solid‐state defect‐based systems. This has motivated the development of a new generation of quantum materials based on paramagnetic molecules that benefit from the unparalleled flexibility and atomic precision offered by synthetic chemistry.^[^ ^1^, ^2^, ^3^, ^4^, ^5^, ^6^ ^]^ Worldwide efforts in scientific and engineering disciplines aim to produce robust qubit platforms that benefit from tailorable and engineered functionality, high‐temperature operation, scalability, and facile integration within diverse electronic, photonic, sensing, and other device platforms.

Electron spins in paramagnetic molecules represent leading qubit candidates since they can be coherently manipulated by microwave or radio‐frequency pulses, which provides the necessary control for initialization, manipulation, and high‐fidelity readout. Different chemical approaches to molecular spin qubits rely on tailoring structures and their surrounding environments to optimize the spin‐lattice relaxation time (*T_1_*), a measure of the qubit lifetime, and the coherence time (*T_2_*), the “survival” time of a qubit in a pure quantum state; important temporal figures of merit which dictate utility in quantum applications. In this context, diverse chemistries have been explored, such as paramagnetic organometallic species, coordination compounds, and organic radicals.^[^ ^1^, ^2^, ^3^, ^4^, ^5^, ^19^, ^20^, ^21^ ^]^ These materials have demonstrated impressive functionalities including tunable coherence lifetimes, entanglement, fundamental qubit operations, and the realization of multiqubit architectures.^[^ ^22^, ^23^, ^24^, ^25^ ^]^ However, only a small fraction demonstrate operationally useful coherence lifetimes above cryogenic temperatures owing to the rapid decoherence of electron spins in molecules.^[^ ^5^ ^]^ Furthermore, molecular design guidelines that promote improved chemical robustness, solid‐state operation, interqubit interactions, and coupling with device functionalities (e.g., optical, electrical, transport) remain nascent, precluding the realization of new technologies.

Organic paramagnetic materials, namely radicals, represent synthetically tunable coherent systems whose light element (e.g., C, H, N, O, S) composition offers weaker spin‐orbit coupling than their inorganic and organometallic counterparts, thereby extending electron spin relaxation and coherence lifetimes. In nearly all cases, these species are monoradicals (spin quantum number, *S* = ½) or low‐spin (*S* = 0) Kekulé diradicaloids.^[^ ^20^, ^25^, ^26^, ^27^, ^28^ ^]^ Organic materials with high‐spin (*S* = 1) ground states are promising targets due to their similarity in electronic structure to leading quantum materials such as NV^−^ centers and defects in SiC whose non‐degenerate spin configuration yields long *T_1_* times, while the low density of nuclear spins prolongs *T_2_*.^[^ ^7^, ^13^ ^]^ Those with delocalized *π*‐systems would not only offer synthetic analogs to these defect‐based systems but also fulfill important needs for qubit design. These include: i) chemical tailorability and atomic level control over spin‐orbit, dipolar, exchange, and hyperfine interactions to enable tunable quantum states, ii) interrelated optical, electronic, and magnetic properties, and iii) the capability to bridge atomic, mesoscopic, and macroscopic scales with tunable solid‐state interactions.^[^ ^1^, ^2^, ^3^, ^4^, ^5^, ^19^ ^]^ Control of these properties is anticipated to enable initialization and readout, controlled interqubit interactions via spin‐spin exchange and/or dipolar interactions, rich spin physics for entanglement, functionality for information storage and processing, and interfacing with device technologies via the interactions of spins with photons, charges, excitons, phonons, magnons, etc.^[^ ^29^ ^]^ However, the inherent instability of organic high‐spin materials precludes their rational design, development, and application. Recently, donor–acceptor (DA) conjugated polymers (CPs) have offered a fundamentally new platform for stabilizing high‐spin ground states^[^ ^30^, ^31^ ^]^ and integrating emerging optoelectronic,^[^ ^32^, ^33^ ^]^ transport,^[^ ^34^ ^]^ spin‐based,^[^ ^35^ ^]^ and magnetic^[^ ^36^ ^]^ functionalities within solid‐state devices. These macromolecules provide new mechanisms of intramolecular exchange that extend beyond prototypical design guidelines for organic open‐shell materials, offering a fundamentally new approach to control structural, electronic, and environmental features that influence spin‐dynamics in *π*‐conjugated materials.

## 2 Design and Synthesis

**Figure 1:**
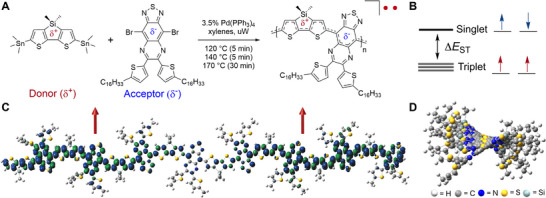
Synthesis of the high‐spin donor–acceptor conjugated polymer and correlation of macromolecular, spin, and topological structure. A) The molecular building blocks and Stille cross‐coupling polymerization used to synthesize the polymer that exhibits B) a high‐to‐low spin energy gap. C) Spin density distribution for the triplet state of the *n* = 8 oligomer modeled at the (U)DFT/B3LYP/6‐31G** level. The blue and green surfaces represent positive and negative contributions of the spin density at an isovalue = 0.02 au. D) Topological structure of the backbone.

Here, we demonstrate the first example of an organic high‐spin qubit based on an open‐shell donor–acceptor (DA) CP, poly[4‐(4,4‐dimethyl‐4*H*‐silolo[3,2‐*b*:4,5‐*b* *′*]dithiophene)‐*alt*‐6,7‐bis(5‐hexadecyl‐thiophen‐2‐yl)‐[1,2,5]thiadiazolo[3,4‐*g*]quinoxaline] (**Figure** **1**). Salient design features include a 4,4‐dimethyl‐4*H*‐silolo[3,2‐*b*:4,5‐*b* *′*]dithiophene donor, which stabilizes the highest occupied MO (HOMO) and strong thiadiazoloquinoxaline acceptor that lowers the lowest unoccupied MO (LUMO). Strategically positioned −C_16_H_33_ substituents on ancillary thiophene units of the acceptor control solubility and facilitate chain conformations with a high degree of electronic coherence by minimizing torsion of the polymer backbone. The alternating copolymer structure promotes strong electronic correlations that form and stabilize unpaired spins within the *π*‐conjugated backbone.^[^ ^30^, ^31^, ^37^ ^]^ The polymer was synthesized using a microwave‐assisted Stille cross‐coupling copolymerization between (4,4‐dimethyl‐4*H*‐silolo[3,2‐*b*:4,5‐*b* *'*]dithiophene)bis(trimethylstannane) and 4,9‐dibromo‐6,7‐bis(5‐hexadecylthiophen‐2‐yl)‐[1,2,5]thiadiazolo[3,4‐*g*]quinoxaline (Figure 1A). Using Pd(PPh_3_)_4_ as the catalyst, we obtained a polymer with a weight average molecular weight (*M*_w_) of 36.8 kg mol^−1^ and a dispersity (*Đ*) of 3.05 determined by gel permeation chromatography (GPC) (Figure S1, Supporting Information). Full details can be found in the Supporting Information. As shown in Figure 1B, this DA combination results in an orbital manifold in which valence α‐ and β‐spins occupy singly occupied MOs (SOMOs) with a characteristic energy splitting that defines the exchange interaction (Δ*E*_ST_ *= 2J*). This value denotes a spin‐flip transition between the ground triplet state |T_0,+,−_⟩ with a total spin *S* = 1 and the singlet state |S⟩ with *S* = 0.

Figure 1C shows the spin density distribution of the triplet state of an oligomer with eight repeat units (*n =* 8) modeled using density functional theory (DFT) at the unrestricted (U)DFT/B3LYP/6‐31G** level of theory. Strong electronic correlations result in extensive delocalization of electron density promoted by internal charge transfer between intrachain electron‐donor (δ^+^) and electron‐acceptor (δ^−^) units. An evolution of the electronic structure arises from rapid HOMO−LUMO configuration mixing as a function of increasing chain length and adoption of an open‐shell ground state at *n* = 4 (Figures S22 and S23, Supporting Information). A further progression from *n* = 4 to 8 enhances spatial separation of progressively weaker interacting α‐ and β‐spins, coincident with a decrease in *J* (|Δ*E*_ST_|  = 0.13 → 0.05 eV from *n*  = 4 → 8; Table S1, Supporting Information). Extrapolation of these data indicates that an inflection point is achieved at *n* = ≈13 (Figure S24, Supporting Information), consistent with the adoption of a high‐spin ground state. This corresponds to an *M*_w_ of ≈13.6 kg mol^−1^, well below the value obtained by GPC. At this critical length, spin delocalization and magnetic properties remain relatively constant and independent of molecular weight (see Section S3.10, Supporting Information). This interplay of electronic hybridization of localized frontier states with Coulomb repulsion between valence electron spins is a hallmark of organic diradicals.^[^ ^26^, ^30^, ^31^ ^]^ However, the spin density distribution maintains extensive spin polarization along the *π*‐conjugated backbone, in stark contrast with small molecule and topologically localized organic spin systems (e.g., organic radicals, polycyclic aromatic hydrocarbons, magnetic edge states in graphene structures).^[^ ^20^, ^25^, ^26^, ^27^, ^28^, ^38^, ^39^, ^40^ ^]^ The bridgehead Si atom has the unique effect of distorting the backbone planarity (Figure 1D), and increasing rotation between donor and acceptor units (dihedral angles (*θ*) of 1.68–7.95° respectively) while preserving electronic coherence (full details in Section S3.9, Supporting Information). Such a framework promotes spin delocalization and structural rigidity and inhibits strong *π*‐stacking and interchain interactions. These features are known to mitigate both intra‐ and interchain decoherence pathways, such as reducing the density of low‐frequency phonons capable of coupling to electron spins within the backbone of a molecular framework and deleterious dipolar and hyperfine interactions in the solid‐state.^[^ ^20^, ^41^, ^42^, ^43^, ^44^ ^]^

## 3 Solid‐State Properties

**Figure 2:**
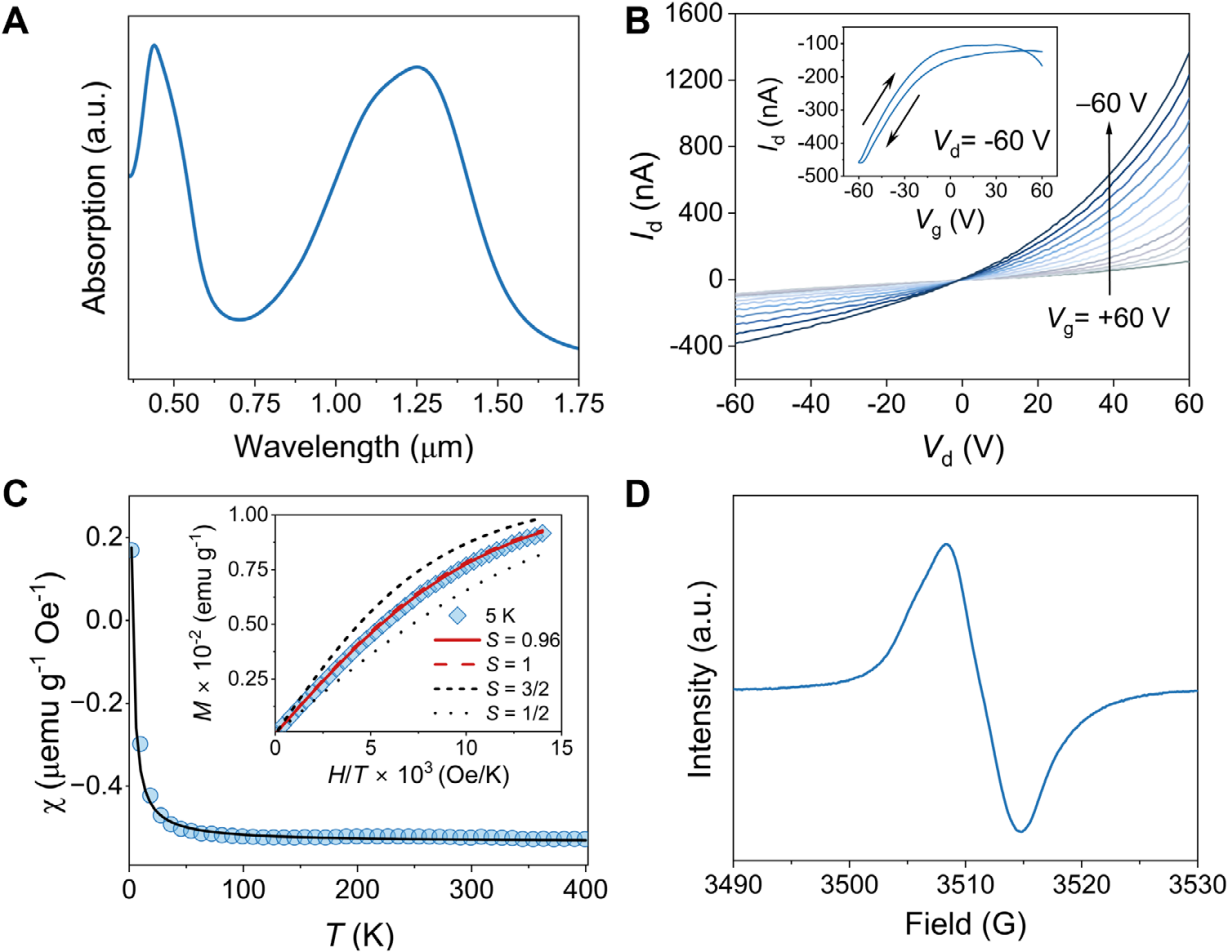
Solid‐state properties of the polymer. A) Absorption spectra of a thin film spin‐coated from chlorobenzene (10 mg mL^−1^) onto a KBr substrate. B) Representative field effect transistor output characteristics (inset: transfer curve) of a spin‐coated film. C) SQUID magnetometry of a solid sample. Main plot: Magnetic susceptibility, *χ* versus *T*, from 2 to 400 K fit to the Curie‐Weiss Law. Inset: Magnetic field (*H*) dependence of the magnetization at 5 K, with Brillouin functions for *S* = 3/2, *S* = 1, *S*  = 1/2, and *S*  = 0.96. The fit with *S* = 0.96 confirms the high‐spin ground state. D) Room temperature continuous‐wave EPR spectra of the solid‐state powder.

The unique topological structure of the copolymer promotes high solubility in common organic solvents. Thus, thin films could be readily processed by spin‐coating solutions onto glass, silicon, or plastic substrates (Figure S3, Supporting Information). As shown in **Figure** **2** **A**, this macromolecule has an absorption maximum (*λ*_max_) of 1.25 µm and measurable absorbance extending beyond the short‐wave infrared (SWIR). These features are attributable to extensive *π*‐conjugation and a high of electronic coherence. The low‐energy absorption correlates with diradical character and a near‐degenerate partially occupied orbital manifold.^[^ ^30^, ^31^, ^34^ ^]^ Cyclic voltammetry (CV) shows distinct peaks for the oxidation and reduction located at −5.38 and −4.47 eV, respectively (Figure S2, Supporting Information). The narrow bandgap is characteristic of DA CPs with open‐shell electronic structures in which unpaired electrons evolve from strong configuration mixing between frontier molecular orbitals.^[^ ^30^, ^31^ ^]^ The transport properties were assessed by fabricating a bottom gate, bottom contact field effect transistor (FET) with the architecture Si/SiO_2_ (300 nm)/octadecyltrichlorosilane/Au (60 nm) (full details in Section S3.3, Supporting Information). Figure 2B shows the transfer current‐voltage (*I*–*V*) characteristics obtained by sweeping the gate bias (*V*_g_) from −60 to 60 V under a source‐drain bias (*V*_d_) of +60 and −60 V. From the transfer curve, the polymer demonstrates p‐type‐dominated FET behavior with a hole mobility (*µ_h_*) of 2.07 × 10^−4^ cm^2^ V^−1^ s^−1^ and intrinsic conductivity (*σ*) of 1.64 × 10^−4^ S cm^−1^ with no discernable changes after repeated cycling (Figure S4, Supporting Information). These transport properties are consistent with narrow bandgap *π*‐conjugated and open‐shell materials.^[^ ^45^ ^]^ Such processing and performance compatibility with current thin‐film‐based optoelectronic technologies is unavailable from other molecular qubit systems.

The solid‐state electronic structure was corroborated by superconducting quantum interference device (SQUID) magnetometry measurements of a solid powder, which revealed that the magnetic susceptibility (*χ*) decreased as temperature increased (Figure 2C), consistent with thermal depopulation of the high‐spin manifold. The magnetization (*M*) versus applied field (*H*) plots at 5 K were fit to the Brillouin function, from which we find *S* = 0.96 (Figure 2C, inset), consistent with the polymer having an *S* = 1 ground state.^[^ ^46^ ^]^ Fitting of the *χ* versus *T* data to the Curie‐Weiss law (*χ* = *C*/(*T*–*θ)* + *χ_0_*), where *C* is the material‐dependent Curie constant, *θ* is the Curie–Weiss temperature constant, and *χ_0_* is a vertical offset that accounts for any observable diamagnetism in the susceptibility, gives a *θ* = −0.56 K, consistent with short‐range antiferromagnetic interactions in the amorphous, paramagnetic polymer matrix.^[^ ^36^ ^]^ Grazing‐incidence wide‐angle X‐ray scattering (GIWAXS, Figure S5, Supporting Information) is consistent with a largely amorphous microstructure and the absence of backbone scattering and *π–π* stacking common in open‐shell diradicals or more quinoidal, rigid, and linear open‐shell macromolecules.^[^ ^31^ ^]^ Thus, the novel topological structure of the backbone and resultant solid‐state morphology can be anticipated to enable protection of the spin states against decoherence by mitigating deleterious dipolar and spin‐phonon interactions.^[^ ^27^, ^41^, ^42^, ^43^, ^44^ ^]^ The room temperature continuous‐wave (CW) EPR spectrum of the solid‐state sample shows a single peak of Voigt lineshape (≈6 G wide) centered at 3511 G with a *g‐*factor of 2.0047 (Figure 2D).^[^ ^47^ ^]^ This is consistent with organic delocalized diradicals having little spin‐orbit coupling from heteroatoms along the *π*‐conjugated backbone.^[^ ^30^, ^36^, ^37^ ^]^ The CW spectrum has a homogeneous broadening contribution of 1.9 G and an inhomogeneous broadening of 5.6 G (15.7 MHz), mainly from hyperfine interactions with ^1^H and ^14^N nuclei in the conjugation path but with a minor contribution from solid‐state *g*‐anisotropy.^[^ ^48^, ^49^ ^]^ Variable‐temperature (VT) EPR data were fit to the Bleaney–Bowers equation in the range of 5–25 K, giving Δ*E*_ST_ = 1.73 ± 0.09 × 10^−2^ kcal mol^−1^ and an exchange coupling constant *J* = 3.02 ± 0.16 cm^−1^. This indicates weak ferromagnetic coupling (*J* > 0) between spins (Figure S9, Supporting Information). This is consistent with the Brillouin fitting of the magnetization in demonstrating a high‐spin triplet ground state. Quantitative EPR revealed a spin count that exhibited no changes over a period of six months under ambient conditions (Figure S8, Supporting Information). The stability of the polymer was further assessed using thermogravimetric analysis (TGA), which revealed that thermal decomposition begins at ≈390 °C (Figure S6, Supporting Information).

## 4 Spin Dynamics

**Figure 3:**
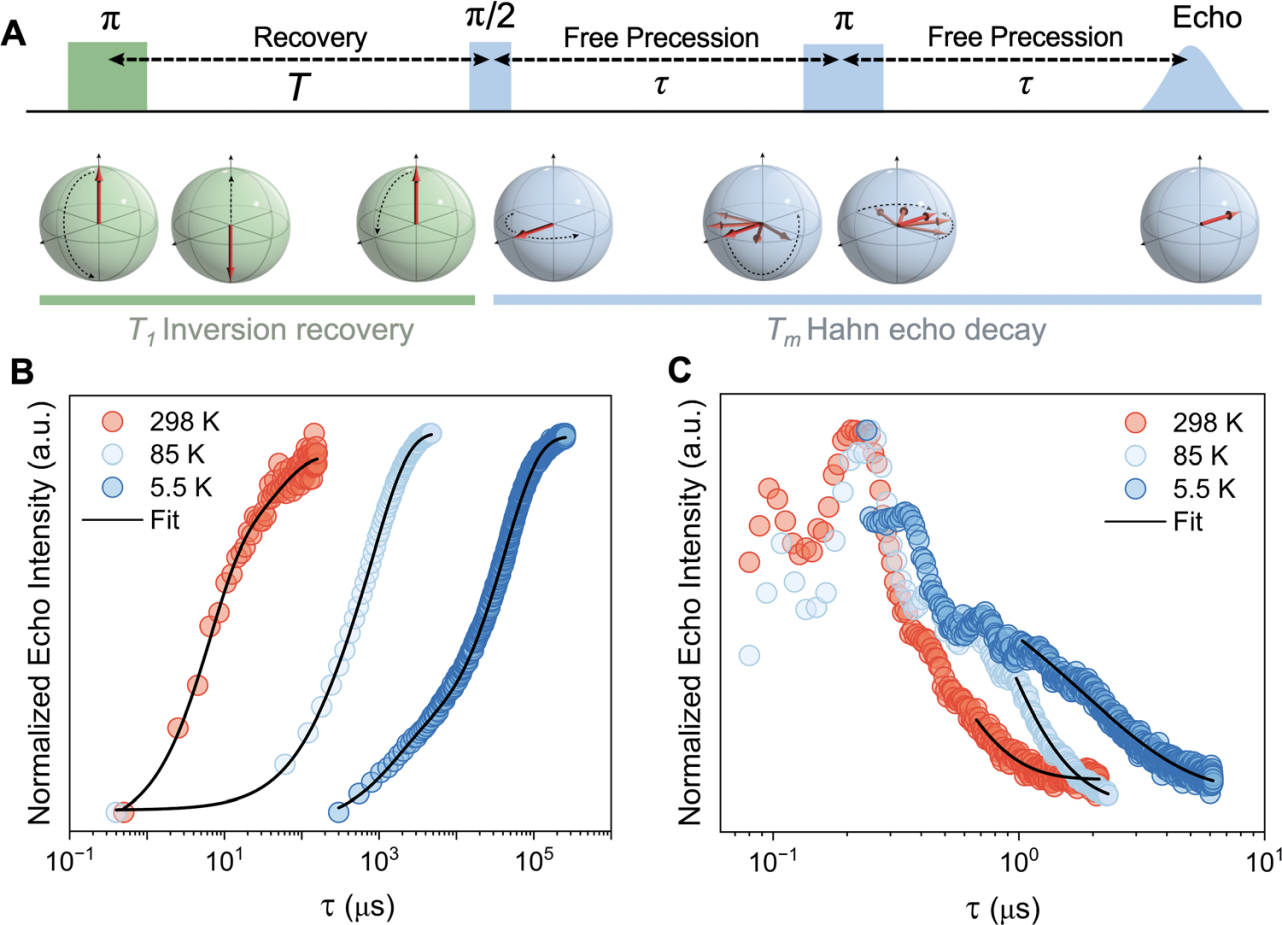
Spin‐lattice and phase memory relaxation times. A) Pulse sequences used to measure the spin‐flip time *T_1_* and the phase memory time *T_m_*. The effect on the Bloch representation of the spin is depicted below the pulse sequence. B) Normalized inversion recoveries with curve fits (black) and C) normalized Hahn echo decays with curve fits (black) at 298, 85, and 5.5 K.

The quantum behavior of the polymer powder was probed using pulsed EPR at 298, 85, and 5.5 K (**Figure** **3**). Two times describe the spin dynamics of electron spins: the spin‐lattice relaxation time (*T_1_*) characterizes the decay of magnetization to equilibrium, and the phase memory time (*T_m_*) characterizes the electron spin coherence and provides an effective lower limit of the pure spin‐spin relaxation time (*T_2_*).^[^ ^50^ ^]^ *T_1_* was determined from the exponential decay after the inversion of the spin magnetization (Figure 3A; see Experimental Section and Section S3.6 (Supporting Information) for detailed explanations of the pulse sequences).^[^ ^48^, ^50^ ^]^ The recoveries were fit to a biexponential decay function with a fast‐relaxing component consistent with decoherence effects such as spectral diffusion, which is associated with stochastic changes in the local magnetic field of observed spins via coupling to neighboring electron or nuclear spins.^[^ ^49^ ^]^ The slow component of the decay thus represents the lower boundary for the spin‐lattice relaxation time and gave values of *T_1_* = 44.3 ± 9 µs, 969 ± 77 µs, and 44.3 ± 0.1 ms at 298, 85, and 5.5 K, respectively (Figure 3B). These values exceed nearly all other molecular spin qubits irrespective of sample state (see Figure S16, Supporting Information for comparison). We attribute this to the rigid *π*‐conjugated backbone and weak spin‐orbit coupling intrinsic to the organic framework, which has been shown to reduce the density of low‐energy phonons and the spin's ability to be affected by vibrational motion, providing an effective shield against spin‐lattice relaxation.^[^ ^41^, ^42^, ^43^, ^44^ ^]^ This material contrasts strongly with other *π*‐conjugated quantum systems, which perform poorly in the solid‐state as a result of strong *π*‐stacking interactions, mitigated here by the backbone torsion produced by the silicon‐bridgehead and steric bulk of the ─C_16_H_33_ sidechains.^[^ ^20^ ^]^

To function in quantum applications (e.g., as a qubit), the *T_m_* coherence time must be long enough to allow precise control and extensive manipulation of spins. As shown in Figure 3C, *T_m_* was measured using the Hahn echo pulse sequence and fit to an exponential decay function, giving *T_m_* = 0.30 ± 0.01 µs, 0.50 ± 0.01 µs, and 1.58 ± 0.01 µs at 298, 85, and 5.5 K, respectively. The initial decay profiles are dominated by strong electron spin echo envelope modulation (ESEEM), characteristic of hyperfine coupling between unpaired spins and the magnetic nuclei in the polymer chain. Three‐pulse (3P) ESEEM measurements (Figure S10, Supporting Information) substantiate this, revealing couplings from 0–4 MHz and ≈15 MHz correlating to the nuclear Zeeman frequencies of ^14^N and ^1^H, respectively. This agrees with the electron density distribution predicted by DFT where both ^14^N and ^1^H nuclei are present in the conjugation pathway and overlap with the regions of high electron density (Figure 1C). Hyperfine sublevel correlation spectroscopy (HYSCORE) (Figure S11, Supporting Information) reveals a complex hyperfine structure consistent with coupling to multiple nuclear spin sources with varying strengths. These sources may be intramolecular, such as repeat units that differ in distances from the unpaired spin, or intermolecular, such as interchain interactions resulting from the solid‐state morphology.^[^ ^27^ ^]^ Interestingly, only weak coupling to ^1^H (presumably from the hydrogens on the donor) is observed despite two ancillary ─C_16_H_33_ side chains per acceptor moiety. This indicates that there is little interaction between the side chains and spin centers, so the solubilizing properties enabling film formation and device integration are not contingent on a detrimental increase in hyperfine coupling.

The *T_m_* was found to depend on the microwave power (Figure S12, Supporting Information), revealing instantaneous diffusion (see Section S3.6, Supporting Information) mediated by interactions between electron spins.^[^ ^48^, ^49^, ^51^ ^]^ These electron‐electron dipolar interactions, together with electron‐nuclear interactions, are the primary sources of decoherence and limit *T_m_* to well below its theoretical limit of *T_1_* (*T_m, max_* = 2*T_1_*). A comparative analysis with an isostructural high‐spin polymer in which the bridgehead silicon atom was substituted for a carbon atom gives *T_m_ =* 130 ns and *T_1_ =* 1.04 µs at 78 K in the solid‐state (Figure S14, Supporting Information). This four fold decrease in *T_m_* and a three‐orders‐of‐magnitude decrease in *T_1_* can be associated with increased backbone planarity emanating from stronger quinoidal bonding in the carbon‐substituted variant.^[^ ^31^ ^]^ This atom‐specific substitution leads to higher levels of molecular ordering, *π‐* *π* stacking, and backbone scattering (Figure S15, Supporting Information). These structural features are a key source of decoherence, dramatically decreasing relaxation times in open‐shell molecular graphenoids.^[^ ^20^ ^]^ These features are absent in the parent silicon‐bridgehead polymer, which has an amorphous microstructure with no *π–π* stacking, thus demonstrating how structural and electronic perturbations within this new molecular framework lead to dramatic changes in quantum properties.

Despite decoherence channels due to the abundance of dipolar interactions in the solid‐state matrix, the silicon‐bridgehead polymer exhibits *T_1_* and *T_m_* values that contrast with leading molecular quantum materials such as organometallic species and organic radicals that only demonstrate measurable lifetimes when diluted in nuclear‐spin‐free solvents or diamagnetic matrices (see Figure S16, Supporting Information for a comparison).^[^ ^20^, ^26^, ^28^ ^]^ Thus, to the best of our knowledge, these solid‐state coherence lifetimes represent the highest values reported for a neutral, synthetic, molecular spin qubit in its native form.

**Figure 4:**
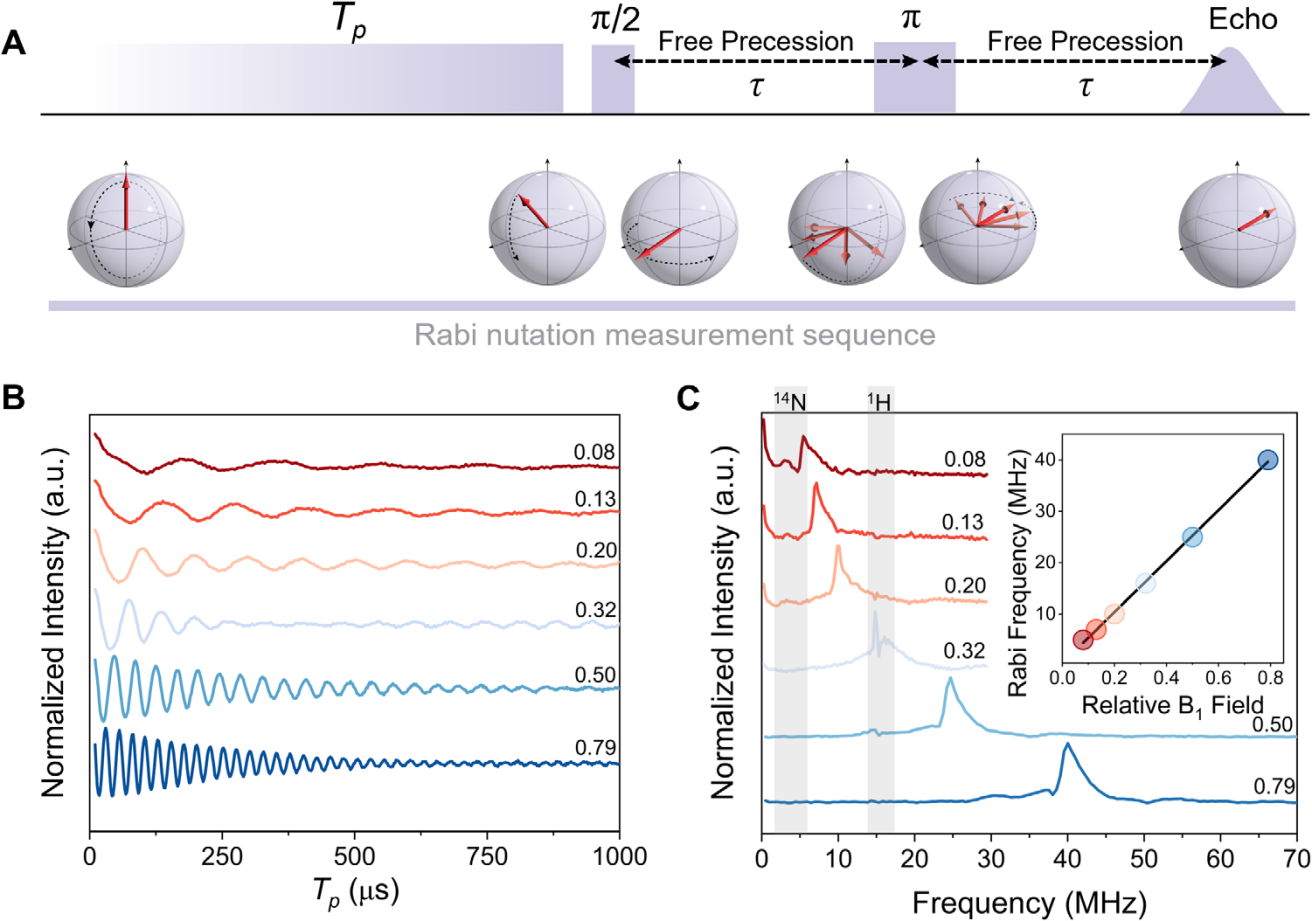
Coherent manipulation of high‐spin qubits. A) Pulse sequence for the measurement of Rabi oscillations. The nutation pulse length (*T_p_*) is incremented to vary the azimuthal position on the Bloch sphere, followed by detection. B) Rabi oscillations at different relative B_1_ field amplitudes, where a relative value of 1 is equal to the B_1_ amplitude generated from a microwave attenuation of 0 dB. C) Fourier transformation of the nutation curves with the linear correlation between Rabi frequency and relative B_1_ field (inset).

The coherent control of spin states and their manipulation on the Bloch sphere is the foundation for their use in quantum information technologies. Rabi beats characterize how well the system can be placed in a quantum superposition by measuring spin nutation driven by strong, resonant microwave fields (B_1_) (**Figure** **4** **A**).^[^ ^3^ ^]^ The polymer gives strong Rabi oscillations at 85 K (Figure 4B) with frequencies proportional to B_1_ (Figure 4C), indicating coherent control of the superposition state and meeting the criteria for single‐qubit operations.^[^ ^29^ ^]^ The amplitude of the Rabi frequency at varying B_1_ field strengths has a range that is relatively constant until the Rabi beat frequency becomes comparable to the CW EPR linewidth (Figure S13, Supporting Information). At that point, the amplitude begins to decrease as spins with different EPR frequencies perform Rabi beats around rather different “tilted” axes or “effective” fields that are the vector sum of the B_1_ field and the resonance offset. The relatively broad signals start to lose intensity when the Rabi frequency reaches ≈20 MHz. The polymer also shows a strong decrease in amplitude for Rabi frequencies roughly matching the ^1^H nuclear Zeeman frequency of 14.6 MHz. The Rabi beats are replaced by long‐lived oscillations from entangled electron‐nuclear spin states created by “matched pulses” and appear in the Rabi spectra as a peak that is quite sharp and intense for ^1^H (Figure 4C).^[^ ^52^ ^]^ Further reduction of B_1_ results in another decrease in amplitude and the emergence of a second peak at ^14^N nuclear Zeeman frequencies (Figure 4C), consistent with entangled electron‐nuclear spin states dominated by the ^14^N nuclear quadrupole interaction. These ^14^N or ^1^H entangled states are a consequence of the hyperfine interactions of the electron spins along the polymer chain which manifest as ESEEM in *T_m_* measurements (Figure 3B) and are consistent with 3P ESEEM and HYSCORE results.

This macromolecular platform provides a novel landscape for quantum technologies far beyond the convenience of working in the solid‐state and at temperatures above liquid helium. The high‐spin polymer supports the coherent manipulation of spins on the Bloch sphere, enabling solid‐state single‐qubit quantum operations. We anticipate that the tunability of the exchange interaction *J*, which has been demonstrated in DA CP systems,^[^ ^31^, ^37^ ^]^ could be used for the design of weakly coupled two‐spin systems meeting criteria to enable multi‐qubit quantum operations, e.g., CNOT and SWAP.^[^ ^23^, ^24^, ^53^ ^]^ Demonstrating single and multi‐qubit operations would satisfy the requirements for a universal set of quantum gates, essential for quantum computation, within a single material platform.^[^ ^29^ ^]^ In the regime of quantum sensing, DA CPs present an attractive platform where the room temperature quantum coherence and sensitivity to the nuclear spin bath could enable the detection of analytes under ambient conditions via modulation of the coherence.^[^ ^6^ ^]^ The transport properties enable coupling to device functionality and electrical readout through methods such as electrically detected magnetic resonance (EDMR), which utilizes the spin‐dependent recombination of charge carriers for the detection of spin states in paramagnetic semiconducting materials.^[^ ^54^, ^55^ ^]^ Additionally, the solution processability and magnetically dense solid‐state phase compatibility enable the fabrication of devices where the qubit signal intensity is not compromised by dilution into a diamagnetic matrix. Such DA CP optoelectronic devices benefit from exceptional stability, with examples demonstrating multi‐year stability with no change in performance.^[^ ^32^ ^]^

In a broader context, DA CPs critically overcome the synthetic inaccessibility, lack of tunability, and intrinsic instability of open‐shell electronic configurations in light‐element quantum materials, while also enabling high‐spin configurations that are similar in electronic structure to leading quantum materials based on solid‐state defect systems and organometallic species. Functionality in the magnetically dense solid‐state phase also avoids the fundamental and critical paradox created by the requisite isolation of spins in other systems, which precludes their realization in functional quantum devices. Recent reports of high‐spin DA CPs have demonstrated unprecedented levels of control over SOMO (de)localization (i.e., the spatial distribution of spins), exchange interactions, electron‐nuclear coupling, magnetic dipolar interactions, and structure and composition.^[^ ^30^, ^31^, ^32^, ^33^, ^34^, ^35^, ^36^ ^]^ These features cannot be systematically controlled within any other quantum materials and offer a novel pathway to tune spin dynamics and quantum properties.^[^ ^4^ ^]^ Moreover, the delocalized nature of the *π*‐systems and interrelated functionalities fulfill numerous important needs for emerging quantum material platforms (e.g., entanglement, addressability, and qubit operations), and offer the ability to interface with device technologies via the interactions of spins with photons, charges, excitons, phonons, and other fundamental and quasiparticle excitations.

## 5 Conclusion

Articulating the complex relationships between molecular, electronic, spin, and quantum properties is a major obstacle in the development of future solid‐state quantum technologies. This work demonstrates a fundamentally new approach toward practically applicable organic, high‐spin qubits that enable coherent control in the solid‐state. The combination of straightforward synthesis and modularity inherent to these macromolecules will enable the practical development of quantum materials with tunable electronic structures, varying magnitudes of intra‐ and intermolecular spin‐spin coupling, cooperative electronic properties based on *π*‐electrons, and interrelated optoelectronic functionalities. Furthermore, the material's properties and quantum coherence relationships reported here demonstrate the suitability of donor–acceptor molecular architectures and high‐spin organic materials for building useful quantum technologies. We anticipate these unique capabilities to facilitate the realization of functional electronic devices with magnetic control and electrical readout via solution‐processing methods, future qubit manipulation by coupling to a variety of optoelectronic, transport, spin, and magnetic degrees of freedom, and multi‐qubit operations based on magnetic resonance.

## 6 Experimental Section

### General Remarks

All manipulations of air and/or moisture‐sensitive compounds were performed under an inert atmosphere using standard glove box and Schlenk techniques. Reagents, unless otherwise specified, were purchased from Fisher Scientific or Sigma–Aldrich and used without further purification. Toluene, tetrahydrofuran (THF), dichloromethane (CH_2_Cl_2_), chloroform (CHCl_3_), ethanol (EtOH), xylenes, and chlorobenzene were degassed and dried over 4 Å molecular sieves prior to use. Deuterated solvents (1,1,2,2‐tetrachloroethane‐*d*_2_ and chloroform‐*d*) were purchased from Cambridge Isotope Laboratories and used as received. Tetrakis(triphenylphosphine)palladium (0) was purchased from Strem Chemicals and used as received. 3,3′‐dibromo‐2,2′‐bithiophene was purchased from Derthon Optoelectronic Materials Science Technology Co. and used as received. 4,9‐dibromo‐6,7‐bis(5‐hexadecylthiophen‐2‐yl)‐[1,2,5]thiadiazolo[3,4‐*g*]quinoxaline was prepared according to previously reported procedures.^[^ ^31^ ^] 1^H and ^13^C nuclear magnetic resonance (NMR) spectra were collected on a Bruker Avance III HD 500 MHz or Bruker Avance III 400 MHz spectrometer, and chemical shifts, δ (ppm), were referenced to the residual solvent impurity peak. Data are reported as: s = singlet, d = doublet, t = triplet, m = multiplet, br = broad, and coupling constants (*J*) are reported in Hertz (Hz). Flash chromatography was performed on a Teledyne Isco CombiFlash Purification System. Microwave‐assisted reactions were performed in a CEM Discover 2.0 microwave reactor. The weight average molecular weight (*M*_w_) and dispersity (*Đ*) were determined by gel permeation chromatography (GPC) at 160 °C in 1,2,4‐trichlorobenzene (stabilized with 125 ppm of BHT) in an Agilent 1260 Infinity II high‐temperature GPC/SEC system using a set of three PLgel 13 µm Olexis columns. Polymer samples were dissolved at a concentration of 1 mg mL^−1^ in 1,2,4‐trichlorobenzene with agitation for 4 h at 160 °C. Additional details regarding materials characterization can be found in the Supporting Information.

### 4,4‐Dimethyl‐4H‐silolo[3,2‐b:4,5‐b']dithiophene

*n*‐Butyllithium (2.5 м in hexanes, 11.13 mL, 27.82 mmol) was added to anhydrous THF (30 mL) in a Schlenk flask and cooled to −90 °C under a flow of nitrogen. 3,3′‐dibromo‐2,2′‐bithiophene (3.01 g, 9.27 mmol) dissolved in anhydrous THF (18 mL) was added dropwise to the mixture, which was stirred for an additional 1 h at −90 °C. Subsequently, dichlorodimethylsilane (2.25 mL, 18.55 mmol) in THF (2 mL) was added dropwise, and the reaction was stirred for an additional 3 h at −90 °C. After this time, the reaction was allowed to warm to room temperature over a period of 12 h. The reaction was quenched with cold deionized (DI) water (30 mL), extracted with CH_2_Cl_2_ (3 × 30 mL), and dried with anhydrous MgSO_4_. The solvent was removed in vacuo, and the crude product was purified by flash column chromatography using hexanes as the eluent to afford 1.473 g (6.623 mmol, 71%) of the product as a white solid. ^1^H NMR (400 MHz, chloroform‐*d*): δ 7.21 (d, *J* = 4.7 Hz, 2H), 7.08 (d, *J* = 4.7 Hz, 2H), 0.42 (s, 6H). ^13^C NMR (126 MHz, chloroform‐*d*) δ 149.16, 142.80, 129.36, 125.32, −3.29. Mass spectrometry (MS) [electrospray ionization (ESI)] exact mass calculated for C_10_H_10_S_2_Si is as follows: *m/z* 223.0066 ([M^+^ + H^+^]) and 223.0064 (found).

### 4,4‐Dimethyl‐2,6‐bis(trimethylstannyl)‐4H‐silolo[3,2‐b:4,5‐b*'*]dithiophene

4,4‐dimethyl‐4*H*‐silolo[3,2‐*b*:4,5‐*b′*]dithiophene (500 mg, 2.25 mmol) was added to an oven‐dried Schlenk flask in a nitrogen filled glovebox and sealed. Anhydrous THF (17 mL) was added, and the solution was cooled to −90 °C under a flow of nitrogen. Lithium diisopropylamide (2.0 м in hexanes, 2.47 mL, 4.96 mmol) was added dropwise, and the reaction mixture was stirred for 30 min at −90 °C, warmed to 0 °C for 30 min, and then cooled to −90 °C. Trimethyltin chloride (1.12 g, 5.62 mmol) in anhydrous THF (2.5 mL) was slowly added dropwise and then stirred for 30 min at −90 °C. The solution was warmed to room temperature, stirred for an additional 30 min, and then quenched with cold DI water (30 mL). The mixture was extracted with hexanes, and the organic layer was washed with DI water (5 × 30 mL), and brine (1 × 30 mL), then dried over anhydrous MgSO_4_. The solvent was removed in vacuo, and purification by flash chromatography on reverse‐phase silica using ethanol (EtOH) containing 1% triethylamine as the eluent gave 694 mg (1.27 mmol, 56%) of the compound as a light brown solid. ^1^H NMR (500 MHz, chloroform‐*d*): δ 7.13 (s, 2H), 0.42 (s, 6H), 0.38 (s, 18H). ^13^C NMR (126 MHz, chloroform‐*d*) δ 155.07, 144.23, 129.38, 125.21, −3.00, −7.96. Mass spectrometry (MS) [electrospray ionization (ESI)] exact mass calculated for C_16_H_26_S_2_SiSn_2_ is as follows: *m/z* 547.9273 ([M^+^ + H^+^]) and 547.9278 (found).

### Polymer Synthesis

A microwave tube was loaded with 4,4‐dimethyl‐2,6‐bis(trimethylstannyl)‐4*H*‐silolo[3,2‐*b*:4,5‐*b* *'*]dithiophene (56.0 mg, 0.102 mmol) and 4,9‐dibromo‐6,7‐bis(5‐hexadecylthiophen‐2‐yl)‐[1,2,5]thiadiazolo[3,4‐*g*]quinoxaline (98.0 mg, 0.102 mmol). The tube was brought inside a nitrogen‐filled glovebox, and 491 µL of a Pd(PPh_3_)_4_/xylenes stock solution (3.50 mol%) was added. The tube was sealed and subjected to the following reaction conditions in a microwave reactor with stirring: 120 °C for 5 min, 140 °C for 5 min, and 170 °C for 30 min. After cooling, the polymer was precipitated into methanol and collected via filtration. The solid was transferred to an extraction thimble and washed (in an inert atmosphere and in the absence of light) with methanol (2 h), acetone (2 h), hexane (12 h), and then acetone (2 h). The polymer was dried in vacuo to give 75 mg (64%) of a black solid. Data are as follows: *M*_w_ = 36.8 kg mol^−1^ and *Đ* = 3.05; ^1^H NMR (600 MHz, 1,1,2,2‐tetrachloroethane‐*d*_2_, 398 K) δ 9.20 (br, 2H), 7.63 (br, 2H), 6.80 (br, 2H), 2.87 (br, 4H), 1.29–1.82 (br, 62H), 0.88 (br, 6H).

### Quantum Chemical Calculations

DFT calculations on model oligomers with 1–8 repeat units (*n* = 1–8) were performed using the Gaussian 16 software package.^[^ ^56^ ^]^ Hexadecyl (─C_16_H_33_) side chains were replaced with methyl (─CH_3_) groups. Geometry optimizations were initially performed using a restricted wave function in the gas phase, followed by broken‐symmetry (BS) calculations. BS‐geometries for singlet and triplet states were further optimized using (U)DFT with Becke's three‐parameter functional (B3LYP) and a 6–31G** basis set.^[^ ^57^ ^]^ The diradical character index (*y*) was calculated at the (U)B3LYP/6‐31G** level of theory from the natural orbital occupancies of the highest occupied natural orbital (HONO) and lowest unoccupied natural orbital (LUNO) using Yamaguchi's formula (Equation S6, Supporting Information).^[^ ^58^ ^]^ Spin density distributions were determined from the occupancy of natural orbitals by examining the difference between α‐ and β‐spin densities.

### SQUID Magnetometry

Magnetometry measurements were carried out on powder samples using a Quantum Design MPMS3 SQUID with vibrating sample magnetometry (VSM). For *χ* versus *T* measurements, the sample was cooled in a 200 Oe magnetic field to 2 K, allowed to reach thermal equilibrium, and the magnetic moment was recorded by SQUID‐VSM upon warming from 2 to 400 K.

For the magnetization as a function of field (*H*) isotherms, the magnetic moment was recorded from −70 000 Oe≤ *H*≤ 70 000 Oe at 5 K. Measurements were started from a low field and ramped up incrementally, allowing the field to stabilize at each step. The data were fit to the Brillouin function for paramagnets to determine the spin quantum number *S*:

(1) $$M=M_{0}\left[\frac{2S+1}{2S}\coth\left(\frac{2S+1}{2S}\frac{gS\mu_{B}H}{k_{B}T}\right)-\frac{1}{2S}\coth\left(\frac{1}{2S}\frac{gS\mu_{B}H}{k_{B}T}\right)\right]$$

where *M_0_* is the saturation magnetization, *g* is the electron *g*‐factor, *µ_B_* is the Bohr magneton, *H* is the applied magnetic field, *k*_B_ is the Boltzmann constant, and *T* is the temperature. All data were background subtracted to account for the VSM capsule and sample holder and corrected for the intrinsic diamagnetism of the sample.

### EPR Spectroscopy

Continuous‐wave EPR measurements were performed using a Bruker ELEXSYS‐II E500 spectrometer operating in the X‐band. Powder samples were loaded into 3 mm high‐purity quartz tubes and evacuated to 0.01 mbar for 3 h before being sealed under an inert atmosphere. Variable‐temperature EPR measurements were collected from 5 to 25 K after achieving thermal equilibrium at each temperature. The integrated signal intensity was fit to the Bleaney–Bowers equation to determine the singlet‐triplet energy splitting (Δ*E*_ST_):

(2) $$I_{\mathrm{EPR}}=\frac{C}{T}\frac{3e^{-2J/k_{B}T}}{1+3e^{-2J/k_{B}T}}$$

where *C* is a constant, *k*_B_ is the Boltzmann constant, *J* is the intramolecular exchange coupling constant, and 2*J* is Δ*E*_ST._

Pulsed EPR measurements were performed on an ELEXSYS‐II E580 spectrometer operating in the X‐band at 5.5, 85, and 298 K. Prior to every sample measurement, all the pulses were phased by applying the Hahn echo sequence at the resonant field and tuning the Hahn echo signal so that the real component was maximized, and the imaginary component was minimized. Phase cycling was used to minimize noise from the defense pulse and eliminate artifacts from unwanted echoes moving across the echo signal.^[^ ^48^ ^]^ Spin coherence was measured via a two‐pulse Hahn echo sequence (*π*/2 – *τ* *– π – τ – echo*) tuned at the proper magnetic field and attenuation with pulse lengths of 16 and 32 ns for *π*/2 and *π*, respectively, and *τ* set to 80 ns. Spin‐lattice relaxation was measured via an inversion recovery sequence (*π – T – π*/2 – *τ – π – τ – echo*) with pulse lengths of 16 and 32 ns for *π*/2 and *π*, respectively, and *T* and *τ* to 500 and 374 ns. The incrementation, number of points, and shot repetition time were optimized depending on the measurement temperature to capture the entire decay. Rabi oscillations were recorded at 85 K via a nutation pulse experiment with an initial nutation pulse length of 10 ns that was incremented by 2 ns with the microwave field attenuated by 0–11 dB, with the echo detection utilizing pulse lengths of 8 and 16 ns for *π*/2 and *π*, respectively. The Rabi oscillations were Fourier transformed to reveal the Rabi frequencies.

## Conflict of Interest

The authors declare no conflict of interest.

## Supporting information



Supporting Information

## Data Availability

The data that support the findings of this study are available in the supplementary material of this article.
