# Supplementary material for: Solid‐State Quantum Coherence From a High‐Spin Donor–Acceptor Conjugated Polymer
Source: Adv Mater. 2025 Sep 6;37(38):2501884. doi: 10.1002/adma.202501884 (PMC12464647; doi:10.1002/adma.202501884)
Supplement: Supplementary file 1 — Supporting Information [file ADMA-37-2501884-s001.pdf]

ADVANCED MATERIALS

Supporting Information

for *Adv. Mater.*, DOI 10.1002/adma.202501884

Solid-State Quantum Coherence From a High-Spin Donor–Acceptor Conjugated Polymer

Alexander J. Bushnell, Tanya A. Balandin, Paramasivam Mahalingam, Chih-Ting Liu, Michael K. Bowman and Jason D. Azoulay**

Supporting Information

Solid-State Quantum Coherence from a High-Spin Donor-Acceptor Conjugated Polymer

Alexander J. Bushnell†, Tanya A. Balandin†, Paramasivam Mahalingam, Chih-Ting Liu, Michael K. Bowman and Jason D. Azoulay**

1. General Remarks
2. Synthesis
3. Experimental Procedures
3.1. UV-Vis-NIR and Fourier Transform Infrared Spectroscopy
3.2. Electrochemistry
3.3. Device Fabrication and Charge Transport Measurements
3.4. Grazing-Incidence Wide-Angle X-ray Scattering (GIWAXS)
3.5. Thermogravimetric Analysis
3.6. Electron Paramagnetic Resonance Spectroscopy
3.7. Superconducting Quantum Interference Device (SQUID) Magnetometry
3.8. Materials Comparison
3.9. Quantum Chemical Calculations
3.10. Spin Delocalization
4. Supporting Figures S1–S39
5. Supporting Tables S1–S14
6. Supporting References

1. General Remarks

All manipulations of air and/or moisture-sensitive compounds were performed under an inert atmosphere using standard glove box and Schlenk techniques. Reagents, unless otherwise specified, were purchased from Fisher Scientific or Sigma-Aldrich and used without further purification. Toluene, tetrahydrofuran (THF), dichloromethane (CH_2Cl_2), chloroform ($CHCl_3$), ethanol (EtOH), xylenes, and chlorobenzene were degassed and dried over 4 Å molecular sieves prior to use. Deuterated solvents (1,1,2,2-tetrachloroethane-d_2 and chloroform-*d*) were purchased from Cambridge Isotope Laboratories and used as received. Tetrakis(triphenylphosphine)palladium (0) was purchased from Strem Chemicals and used as received. 3,3'-dibromo-2,2'-bithiophene was purchased from Derthon Optoelectronic Materials Science Technology Co. and used as received. 4,9-dibromo-6,7-bis(5-hexadecylthiophen-2-yl)-[1,2,5]thiadiazolo[3,4-*g*]quinoxaline was prepared according to previously reported procedures.[1] ^{1}H and ^{13}C nuclear magnetic resonance (NMR) spectra were collected on a Bruker Avance III HD 500 MHz or Bruker Avance III 400 MHz spectrometer, and chemical shifts, δ (ppm), were referenced to the residual solvent impurity peak. Data are reported as: s = singlet, d = doublet, t = triplet, m = multiplet, br = broad, and coupling constants (*J*) are reported in Hertz (Hz). Flash chromatography was performed on a Teledyne Isco CombiFlash Purification System. Microwave-assisted reactions were performed in a CEM Discover 2.0 microwave reactor. The weight average molecular weight (M_w) and dispersity (*Đ*) were determined by gel permeation chromatography (GPC) at 160 °C in 1,2,4-trichlorobenzene (stabilized with 125 ppm of BHT) in an Agilent 1260 Infinity II high-temperature GPC/SEC system using a set of three PLgel 13 μm Olexis columns. Polymer samples were dissolved at a concentration of 1 mg mL^{-1} in 1,2,4-trichlorobenzene with agitation for 4 hours at 160 °C. Additional details regarding materials characterization can be found in the Supporting Information.

2. Synthesis

4,4-Dimethyl-4*H*-silolo[3,2-*b*:4,5-*b'*]dithiophene *n*-Butyllithium (2.5 M in hexanes, 11.13 mL, 27.82 mmol) was added to anhydrous THF (30 mL) in a Schlenk flask and cooled to –90 °C under a flow of nitrogen. 3,3'-dibromo-2,2'-bithiophene (3.01 g, 9.27 mmol) dissolved in anhydrous THF (18 mL) was added dropwise to the mixture, which was stirred for an additional 1 hour at –90 °C. Subsequently, dichlorodimethylsilane (2.25 mL, 18.55 mmol) in THF (2 mL) was added dropwise, and the reaction was stirred for an additional 3 hours at -90 °C. After this time, the reaction was allowed to warm to room temperature over a period of 12 hours. The reaction was quenched with cold deionized (DI) water (30 mL), extracted with CH_2Cl_2 (3 × 30 mL), and dried with anhydrous $MgSO_4$. The solvent was removed in vacuo, and the crude product was purified by flash column chromatography using hexanes as the eluent to afford 1.473 g (6.623 mmol, 71%) of the product as a white solid. ^{1}H NMR (400 MHz, chloroform-*d*): δ 7.21 (d, *J* = 4.7 Hz, 2H), 7.08 (d, *J* = 4.7 Hz, 2H), 0.42 (s, 6H). ^{13}C NMR (126 MHz, chloroform-*d*) δ 149.16, 142.80, 129.36, 125.32, -3.29. Mass spectrometry (MS) [electrospray ionization (ESI)] exact mass calculated for $C_{10}H_{10}S_2Si$ is as follows: *m/z* 223.0066 ([$M^+ + H^+$]) and 223.0064 (found).

4,4-Dimethyl-2,6-bis(trimethylstannyl)-4*H*-silolo[3,2-*b*:4,5-*b'*]dithiophene 4,4-dimethyl-4*H*-silolo[3,2-*b*:4,5-*b*']dithiophene (500 mg, 2.25 mmol) was added to an oven-dried Schlenk flask in a nitrogen filled glovebox and sealed. Anhydrous THF (17 mL) was added, and the solution was cooled to -90 °C under a flow of nitrogen. Lithium diisopropylamide (2.0 M in hexanes, 2.47 mL, 4.96 mmol) was added dropwise, and the reaction mixture was stirred for 30 minutes at -90 °C, warmed to 0 °C for 30 minutes, and then cooled to -90 °C. Trimethyltin chloride (1.12 g, 5.62 mmol) in anhydrous THF (2.5 mL) was slowly added dropwise and then stirred for 30 minutes at -90 °C. The solution was warmed to room temperature, stirred for an additional 30 minutes, and then quenched with cold DI water (30 mL). The mixture was extracted with hexanes, and the organic layer was washed with DI water (5 × 30 mL), brine (1 × 30 mL), then dried over anhydrous $MgSO_4$. The solvent was removed in vacuo, and purification by flash chromatography on reverse-phase silica using ethanol (EtOH) containing 1% triethylamine as the eluent gave 694 mg (1.27 mmol, 56%) of the compound as a light brown solid. ^{1}H NMR (500 MHz, chloroform-*d*): δ 7.13 (s, 2H), 0.42 (s, 6H), 0.38 (s, 18H). ^{13}C NMR (126 MHz, chloroform-*d*) δ 155.07, 144.23, 129.38, 125.21, -3.00, -7.96. Mass spectrometry (MS) [electrospray ionization (ESI)] exact mass calculated for $C_{16}H_{26}S_2SiSn_2$ is as follows: *m/z* 547.9273 ([$M^+ + H^+$]) and 547.9278 (found).

Poly[4-(4,4-dimethyl-4*H*-silolo[3,2-*b*:4,5-*b'*]dithiophene)-*alt*-6,7-bis(5-hexadecylthiophen-2-yl)-[1,2,5]thiadiazolo[3,4-*g*]quinoxaline] A microwave tube was loaded with 4,4-dimethyl-2,6-bis(trimethylstannyl)-4*H*-silolo[3,2-*b*:4,5-*b*']dithiophene (56.0 mg, 0.102 mmol) and 4,9-dibromo-6,7-bis(5-hexadecylthiophen-2-yl)-

[1,2,5]thiadiazolo[3,4-*g*]quinoxaline (98.0 mg, 0.102 mmol). The tube was brought inside a nitrogen-filled glovebox, and 491 µL of a $Pd(PPh_3)_4$/xylenes stock solution (3.50 mol%) was added. The tube was sealed and subjected to the following reaction conditions in a microwave reactor with stirring: 120 °C for 5 minutes, 140 °C for 5 minutes, and 170 °C for 30 minutes. After cooling, the polymer was precipitated into methanol and collected via filtration. The solid was transferred to an extraction thimble and washed (in an inert atmosphere and the absence of light) with methanol (2 hours), acetone (2 hours), hexane (12 hours), and then acetone (2 hours). The polymer was dried in vacuo to give 75 mg (64 %) of a black solid. Data are as follows: M_w =36.8 kg mol^{-1} and $Đ$ = 3.05; ^{1}H NMR (600 MHz, 1,1,2,2-tetrachloroethane-d_2, 398 K) δ 9.20 (br, 2H), 7.63 (br, 2H), 6.80 (br, 2H), 2.87 (br, 4H), 1.29–1.82 (br, 62H), 0.88 (br, 6H).

3. Experimental Procedures

3.1 UV–vis–NIR and Fourier transform infrared spectroscopy. UV–vis–NIR and Fourier transform infrared (FTIR) spectra were recorded from 0.20 to 3.30 µm and from 1.3 to 25.0 µm using a Cary 5000 UV–vis–NIR spectrophotometer and Shimadzu IRAffinity-1 FTIR spectrometer, respectively. Thin films were prepared by spin coating a chlorobenzene solution (10 mg mL^{-1}) onto quartz or KBr substrates at 1000 rpm.

3.2 Electrochemistry. Electrochemical characteristics were determined by cyclic voltammetry (50 mV s^{-1}) carried out on drop-cast polymer films at room temperature in degassed anhydrous acetonitrile with tetrabutylammonium hexafluorophosphate (0.1 M) as the supporting electrolyte. The working electrode was a platinum wire, the counter electrode was a platinum wire, and the reference electrode was Ag/AgCl in 3M KCl. The potential axis was calibrated to the reference electrode +0.21 V vs. the normal hydrogen electrode (NHE). Then, oxidation and reduction onsets were corrected by -4.44 eV for NHE vs. vacuum to determine ionization potentials and electron affinities. The onset of oxidation was identified at -5.38 eV, and the onset of reduction was estimated as -4.47 eV (**Figure S2**).

3.3 Device Fabrication and Charge Transport Measurements. The hole mobility and organic field-effect transistor (OFET) characteristics were evaluated using a typical bottom-gate, bottom-contact geometry. To prepare the Si/SiO_2 substrates (2.0 cm × 1.5 cm), they were first cut and cleaned thoroughly. Substrates were washed with a 2% Hellmanex detergent solution in DI water, followed by DI water, acetone, and 2-isopropanol, each with 10 minutes of sonication. The cleaned substrates were then dried using nitrogen with a filtered nozzle and baked in an oven for at least one hour. Afterward, the substrates undergo UV/ozone cleaning for 20 minutes before being immediately transferred into a glovebox. Inside the glovebox, gold (Au) electrodes (60 nm thick, deposited at a rate of 1 Å s^{-1}) were deposited on a chromium adhesive layer (5 nm thick, rate: 0.1 Å s^{-1}) using a shadow mask (L = 30, 40, 50, 60, 80 µm, W = 1000 µm). The heavily n-doped silicon substrates with a 300 nm thermally grown SiO_2 dielectric were prepared as the bottom-gate electrode. Subsequently, 3 mM octadecyl trichlorosilane ($CH_3(CH_2)_{17}SiCl_3$ (OTS)) in trichloroethylene (TCE) was applied to the pre-fabricated substrates by spin-coating at 3000 rpm for 10 seconds, after allowing the solution to sit for 15 seconds. The substrates were dried in the glovebox for 15 minutes, washed twice with hexane, and then dried for 2 hours before being removed from the glovebox. The OTS-treated substrates were then exposed overnight to ammonium hydroxide (28-30% in water) in a vacuum desiccator to promote hydrolysis. Any residual OTS was then removed by ultrasonication in toluene for 2 minutes, followed by rinsing with hexane and acetone. The substrates were dried with nitrogen before being transferred back into the glovebox, where a 10 mg mL^{-1} polymer solution was spin-cast using 1000 rpm for 60 s onto the substrate with pre-patterned Au electrodes. The polymer is highly soluble in common organic solvents and could be readily processed onto glass, silicon, or plastic substrates (**Figure S3**).

Devices were tested on a probe station (Signatone 1160 series) inside a nitrogen-filled glovebox, and the data were recorded on a Keysight B1500A semiconductor characterization system. The hole mobility was extracted from the linear region of the transfer curve in a transistor geometry based on Eq. S1:

$$\mu = \frac{L}{WC_iV_D}\frac{\Delta I_D}{\Delta V_G} \qquad \text{(Eq. S1)}$$

where μ, L, W, C_i, V_D, I_D, and V_G represent the mobility, channel length, channel width, capacitance of the dielectric layer (300 nm thick SiO_2 layer), drain voltage, drain current, and gate voltage, respectively.[2]

3.4 Grazing-Incidence Wide-Angle X-ray Scattering (GIWAXS). GIWAXS samples were prepared by spin-coating a film at 1000 rpm for 60 s from a 10 mg mL^{-1} chlorobenzene solution onto a Si wafer and thermally annealing

at 110˚C for 15 minutes. The static GIWAXS experiments were carried out at the 11-BM beamline at Brookhaven National Laboratory. The scattering profile was measured at an incidence angle of 0.12 with a 10 s exposure time. The X-ray beam had an energy of 13.5 keV with a 0.2 mm (height) and 0.05 mm (width) size. The sample-to-detector distance was 26 cm. The data was analyzed using the Sci-Analysis package provided by the beamline. Horizontal (in-plane) and vertical (out-of-plane) sector averages were calculated at 20 degrees. In **Figure S5,** the film scattering is consistent with a largely amorphous microstructure as seen by the scattering rings in the 2D profile and the lack of sharp diffracting peaks in the 1D profiles of in-plane and out-of-plane measurements. It is possible to resolve a weak in-plane ordering at a q ~ 0.1 to 0.25 $Å^{-1}$, which is affiliated with alkyl stacking of the polymer chains.[3]

3.5. Thermogravimetric Analysis (TGA). TGA was conducted on a Mettler Toledo TGA2 STAR System Thermogravimetric Analyzer by heating from room temperature to 800 °C at a heating rate of 20 °C min^{-1} under a N_2 flow of 20 mL min^{-1}. The polymers displayed good thermal stability with thermal decomposition beginning at ~390 °C, marked by 5% weight loss (**Figure S6**).

3.6. Electron Paramagnetic Resonance Spectroscopy (EPR). Room temperature continuous-wave EPR spectra were recorded on a Bruker ELEXSYS-II E500 CW EPR spectrometer operating in the X-band. Solid-state samples were loaded into 3 mm high-purity quartz tubes and evacuated to 0.01 mbar for 3 hours in a hot sand bath before being sealed with epoxy resin under an inert atmosphere. The *g*-factor of 2.0047 was confirmed by fitting the acquired CW spectra to a MATLAB EasySpin simulation accounting for a triplet with an isotropic *J*-coupling (**Figure S7**).[4] Due to the lack of fine structure at the X-band, simulations of the EPR spectrum do not provide any certain information beyond the isotropic *g*-value, homogeneous and inhomogeneous broadening. Spin concentration was obtained by comparing solid-state samples against a 2,2-diphenyl-1-picrylhydrazyl standard. Spin counts of solid samples stored at room temperature under a nitrogen atmosphere showed no discernible changes over a period of 6 months (**Figure S8**).

To more definitively corroborate the high-spin ground state, we performed variable temperature (VT) EPR measurements to directly measure the singlet-triplet energy splitting (ΔE_{ST}) (**Figure S9**). By integrating the CW signal collected over a range of 5-25 K and plotting it versus $1/T$, ΔE_{ST} can be extracted by fitting to the Bleaney-Bowers equation (Eq. S2):

$$I_{EPR}=\frac{C}{T}\frac{3e^{-2J/k_BT}}{1+3e^{-2J/k_BT}} \qquad \text{(Eq. S2)}$$

where C is a constant, k_B is the Boltzmann constant, J is the intramolecular exchange coupling constant, and $2J$ is $\Delta E_{ST.}$ These results reveal that the signal intensity decreases as temperature increases, consistent with a high-spin (triplet) ground state.

Pulsed EPR measurements were done on the ELEXSYS-II E580 spectrometer operating in the X-band at different temperatures: 5.5 K, 85 K, and room temperature. Prior to every sample measurement, all the pulses were phased by applying the Hahn echo sequence at the resonant field and tuning the Hahn echo signal so that the real component was maximized and the imaginary component was minimized. Phase cycling was used to minimize noise from the defense pulse and eliminate artifacts from unwanted echoes moving across the echo signal.[5]

Spin coherence was measured via a two-pulse Hahn echo sequence ($\pi/2$ - t - π - t - *echo*) with pulse lengths of 16 ns and 32 ns $\pi/2$ and π, respectively, and τ set to 80 ns. These pulse lengths were optimized via nutation pulse experiments and tuned for measurement at the proper magnetic field and attenuation for every sample. The nutation pulse experiment starts at very short pulse lengths, followed by a Hahn echo measurement, and the initial pulse is then incrementally increased.[7] In the resulting sine trend, half of the first oscillation gives the ideal π pulse length. Spin-lattice relaxation was measured via an inversion recovery sequence (π - T - $\pi/2$ - τ - π - τ - *echo*) with pulse lengths of 16 ns and 32 ns for $\pi/2$ and π, respectively, and T and τ to 500 ns and 374 ns. The delay between the inverting pulse and the Hahn echo sequence component was incremented by 2000 ns (d_{30}) at room temperature. The incrementation, number of points, and shot repetition time were optimized depending on the measurement temperature to capture the entire decay.

Rabi oscillations were measured at 85 K via a nutation pulse experiment using an initial nutation pulse length of 10 ns that was incremented by 2 ns, with the echo detection utilizing pulse lengths of 8 ns and 16 ns for $\pi/2$ and π,

respectively. Rabi oscillations were measured at different attenuations from 0 dB to 11 dB. This data was then Fourier transformed to resolve the Rabi frequencies. Attenuation was converted to a relative B_1 field (relative to the maximum B_1 amplitude at 0 dB attenuation) to show the linear relationship with Rabi frequency.

Three-pulse ESEEM measurements, with a pulse sequence of ($\pi/2$ - τ - $\pi/2$ - T - $\pi/2$ - τ - *stimulated echo*), were conducted to further verify the presence of ^{14}N and ^{1}H hyperfine coupling. The stimulated echo decays more slowly because it is more sensitive to T_1 and gives rise to sharper characteristic frequency peaks.[6] The pulse length was set to 16 ns, τ was set to 240 ns, and T was set to 40 ns, which is incremented by 14 ns every repetition. The real value of the complex time domain data was taken, and a baseline correction was applied, followed by a Hamming window function and a zero fill to 1024 points. A fast Fourier transformation then gave the frequency domain with a positive peak at the proton nuclear Zeeman frequency and several peaks below 5 MHz corresponding to ^{14}N with complex amplitudes resulting from the nuclear Zeeman and quadrupole interaction of a nucleus with I = 1 (**Figure S10**). Hyperfine sublevel correlation (HYSCORE) was also performed and is a two-dimensional three-pulse ESEEM technique in which the transient states evolve in two different electron spin manifolds.[5] Fourier transform of the data produces a two-dimensional frequency domain with cross peaks that are symmetric to the diagonal of the quadrants and correspond to nuclear spin transition frequencies. In **Figure S11**, the weakly coupled (+,+) and the strongly coupled (-,+) quadrants demonstrate peaks at the nuclear Zeeman frequency of ^{14}N splitting at varying frequencies and intensities in addition to weak ^{1}H coupling.

Instantaneous diffusion is an effect that occurs in systems with high spin density and allows for the characterization of interactions between electron spins. The π pulse in the Hahn echo detection sequence flips off-resonant or "unobserved" spins, causing a change in the dipolar interaction with resonant "observed" spins, which instantaneously shifts ΔB and shortens the measured coherence lifetime.[7] It can be studied by measuring the Hahn echo decay as a function of the π pulse turning angle (by either changing the second pulse width or microwave power attenuation). As the turning angle of the second pulse decreases, the impact of instantaneous diffusion also decreases because fewer unobserved spins are flipped, resulting in less impact on the observed spins. The T_m becomes longer as the turning angle decreases from π when there is significant instantaneous diffusion. **Figure S12** demonstrates that there is instantaneous diffusion in the solid-state as T_m increases, ~40 ns at a 3 dB higher attenuation.

3.7 Superconducting Quantum Interference Device (SQUID) Magnetometry. Magnetometry data were collected using the Quantum Design MPMS3 SQUID-VSM. For the magnetic susceptibility as a function of temperature measurements, the magnetic moment was recorded by SQUID-VSM upon warming over the range 2–400 K after cooling in a 200 Oe magnetic field and allowing the sample to reach thermal equilibrium (10 minutes) at 2 K. For the magnetization as a function of applied magnetic field isotherms, the magnetic moment was recorded, after allowing the sample to reach thermal equilibrium (10 minutes) at the measurement temperature, within the range -70,000 Oe $\leq H \leq$ 70,000 Oe. The field sweeps began from -70,000 Oe field, ramping up incrementally, and allowing the field to stabilize at each step before recording. The background signal of the sample holder and an empty VSM capsule of the same length as the one filled with the polymer sample were recorded using the same measurement sequences and subtracted from the original signal. The mass magnetic susceptibility χ was determined from the magnetic moment by Eq. S3, where the magnetization M is defined as the magnetic moment per unit mass and H is the applied magnetic field.[8]

$$\chi = \frac{M}{H} \quad \text{(Eq. S3)}$$

The modified Curie-Weiss Law (Eq. S4) for paramagnets, where C is the material-dependent Curie constant, T is temperature, θ is the Curie-Weiss temperature constant, and χ_0 is for any observable offset in magnetic susceptibility, fits to the χ versus T data, and gives θ = -0.559 K.

$$\chi = \frac{C}{(T-\theta)} + \chi_0 \quad \text{(Eq. S4)}$$

A classical paramagnet assumes spins are noninteracting (θ = 0), so the negative Curie-Weiss temperature constant indicates the presence of weak, short-range antiferromagnetic interactions among populations of nearest-neighbor spins. The magnetization versus applied field data was fit to the Brillouin function (Eq. S5) for paramagnets: [9] [10]

$$M = M_0\left[\frac{2S+1}{2S}\coth\left(\frac{2S+1}{2S}\frac{gS\mu_B H}{k_B T}\right) - \frac{1}{2S}\coth\left(\frac{1}{2S}\frac{gS\mu_B H}{k_B T}\right)\right] \quad \text{(Eq. S5)}$$

where M_0 is the saturation magnetization, g is the electron g-factor, μ_B is the Bohr magneton, H is the applied magnetic field, k_B is the Boltzmann constant, T is temperature, and S is the spin quantum number of the material system and fitting parameter in this equation (**Figure 2C,** inset). The magnetization versus applied field data was corrected for the intrinsic diamagnetic contributions and the high-field magnetic saturation in the sample. This is often crucial for organic diradicals prior to fitting to the Brillouin function because the weak paramagnetic moment of the backbone can saturate at lower fields, and the diamagnetic contribution from orbital diamagnetism and closed-shell components can affect the line shape (e.g., the $–C_{16}H_{33}$ alkyl chains that enable solubilizing properties).[1, 11] Fitting the data collected at 5 K gives a spin quantum number of S=0.96. This slightly deviates from the theoretical S=1 value for high-spin diradicals due to the low-temperature antiferromagnetic interactions between spins.

3.8 Materials Comparison. To clearly define the connection between the donor and acceptor moieties selected here and spin coherence control, we synthesized and compared an isostructural polymer in which the bridgehead silicon atom **Si**$(CH_3)_2$ was substituted with carbon, i.e., **C**$(CH_3)_2$. The spin dynamics of the **C**$(CH_3)_2$ variant were characterized in the solid-state using pulsed EPR with inversion recovery and Hahn echo decays at 78 K, revealing substantially shorter T_m and T_1 of 130 ns and 1.04 µs, respectively (**Figure S14**). These values represent a four-fold decrease in phase memory time and a one-thousand-fold decrease in spin-lattice relaxation compared to the parent Si-bridgehead polymer. We attribute these changes to an increase in intermolecular ordering as a result of the enhanced backbone planarity of the C-bridgehead polymer (**Figure S15**). This change is a result of the enhanced quinoidal bonding pattern in the C-bridgehead and shorter interannular bonds, decreasing the rotation between donor and acceptor units (i.e., dihedral angles (θ)), thereby generating a more rigid, planar structure. GIWAXS measurements corroborate this and reveal the emergence of a distinct out-of-plane peak at q~1.75 Å^{-1} corresponding to π-π stacking (**Figure S15**) along with in-plane (001) peaks at q ~0.54 Å^{-1} (d ~11.6 Å) and weak (200) peaks at q ~0.38 Å^{-1}, which can be attributed to backbone scattering along with additional side-chain ordering. This has been demonstrated to significantly decrease relaxation times in open-shell graphenoids, which is consistent with the results described here.[12] However, this intermolecular and solid-state ordering is mitigated by atom-specific substitution with the Si-bridgehead leading to increased rotation between donor and acceptor units (θ of 1.68 – 7.95° respectively), giving rise to a higher degree of backbone torsion. GIWAXS measurements under the same sample preparation conditions show an amorphous microstructure with no π-π stacking (**Figure S5**).

3.9 Quantum Chemical Calculations. In recent years, computational tools have been utilized to describe the open-shell character of small molecules and polymeric systems. The contributions of strong π-correlations between unpaired electrons to the ground state electronic structure can be described in terms of the singlet-triplet energy gap (ΔE_{ST}), diradical character index (y), spatial overlap of electron spins, spin density distribution between the α- and β-frontier molecular orbitals (FMOs), nucleus independent chemical shift ($NICS_{(1)iso}$), electrostatic potential (ESP) surface, anisotropy of the induced current density (ACID), and bond length alternation (BLA). DFT calculations on the model oligomer units were performed by progressively increasing the size of the π-system from n = 1 to 8 using the Gaussian 16 software package.[13] Hexadecyl ($–C_{16}H_{33}$) side chains were truncated with methyl ($–CH_3$) groups. All energy-minimized geometries obtained from semi-empirical methods (PM6) were subjected to optimization calculations using the spin-restricted Becke's three-parameter (B3LYP) density functional and 6-31G** basis set.[14] Broken-symmetry (BS) formalism was adopted with a restricted wave function and applied to generate the initial guess wave function with a 1:1 mixture to remove α and β and spatial symmetry variation, with $\langle S^2\rangle$ values that are different from 0 (pure singlet) and 2 (pure triplet).[15] However, the wave functions corresponding to oligomers with a π-conjugation length of n = 4 to 8 showed restricted-to-unrestricted instability and were further subjected to a wave function stability test, providing a stable wave function with singlet-triplet electronic ground states at the (U)DFT/B3LYP/6-31G** level of theory. The diradical character index (y) at the same level of theory has been evaluated from HONO and LUNO occupancies by following Yamaguchi's formula (Eq. S6): [16]

$$y = 1 - \frac{2T}{1 - T^2} \quad \text{(Eq. S6)}$$

where T is defined as the orbital overlap that can be calculated using the occupation numbers (n) obtained from the unrestricted natural orbitals in equation (Eq. S7):

$$T = \frac{n_{HONO} - n_{LUNO}}{2} \quad \text{(Eq. S7)}$$

Spin locations were predicted from the natural spin densities of Kohn-Sham molecular orbitals (MO). The molecular electrostatic potential surface (MESP) and FMOs involved in electron and spin density distribution analyses were calculated using the (U)DFT/B3LYP/6-311G** functional and basis set. $NICS_{iso(1)}$ calculations were performed using the gauge-independent atomic orbital (GIAO) method on the BS optimized geometry to assess the effect of the ring current produced by the π-electrons from each ring of the model oligomers. To diminish contributions of σ-bonding to the π-ring current, a so-called ghost atom was placed 1 Å perpendicular to the ring plane. The obtained values have been generally reported as the negative value of the absolute isotropic magnetic shielding, where large negative NICS values indicate a more pronounced aromaticity containing ($4n$+2) π-electrons, while the smaller positive or negative values suggest an involvement of quinoidal characteristics with $4n$ π electrons. ACID calculations were employed to evaluate the flow of ring current density over the π-framework in terms of global and local aromaticity induced by the donor and acceptor units upon π-extension. Natural bond order (NBO) analysis was performed to evaluate the hybridization between the bridged Si atoms and proximal carbons. A large basis set of 6-311++G** was used for NICS, NBO, and ACID calculations to obtain results with better accuracy.

Oligomers with n = 1, 2, 4, 6, and 8 were studied using unrestricted DFT methods to evaluate the impact of the bridged Si atom and qualitatively relate the electronic structural evolution to the spin and magnetic properties of the polymer. The monomer and dimer (n = 1 and 2) adopt a closed-shell ground state (y = 0) with a large $|\Delta E_{ST}|$ of 0.769 and 0.352 eV, respectively. $|\Delta E_{ST}|$ is reduced to 0.133 eV in the tetramer (n = 4) as open-shell (unrestricted) characteristics become lower in energy, resulting in a y of 0.043 (**Figures S22, S23**). Further increasing the oligomer to n = 6 enhances the open-shell ground state spin configuration (y = 0.318) with the singlet (low-spin) state possessing a mixed aromatic-quinoidal-bonding pattern and delocalized spin density distribution over the π-molecular framework. The diradical character increases rapidly with conjugation length and a concomitant decrement of $|\Delta E_{ST}|$, reaching 0.05 eV for n = 8 (**Table S1**). Extrapolation of these data (**Figure S24**) indicates that an inflection point is achieved at n = ~13, where ΔE_{ST} reaches 0 and the ground state becomes high-spin. This transition is facilitated by a shift from aromatic to quinoidal bonding as a result of the π-extension and is substantiated by BLA analysis of the n = 8 octamer showing decreased interannular bond lengths at the octamer core (**Figures S27, S28, and Table S9-S13**). This is accompanied by a progressive localization of the spin density at the peripheral D-A units (**Figures S29-S34**), which is consistent with previous studies of open-shell ground state triplet conjugated polymers.

The hybridization pattern between the bridgehead Si ($sp^{3.22}d^{0.03}$) atom and proximal carbon ($sp^{2.13}$) atoms from the bonding and anti-bonding orbital interactions obtained via NBO analysis verifies a significant contribution from the overlap of $d\pi$-$p\pi$ orbital interactions (**Table S2-S3**). This effect mitigates the π-delocalization between the donor and acceptor units and deteriorates the planarity of the molecular framework, leading to dihedral angles ranging from 1.68 – 7.95° between the D-A units (**Figure S25**). However, the electron density of α- and β-SOMOs are still well-delocalized over the π-molecular framework, and delocalization patterns in the α- and β-SUMOs are nearly identical (only changing in orbital phase), a clear indication of configurational admixing between frontier energy levels. Further, the nearly identical values of 0.038 and 0.040 eV computed from the degree of degeneracy (Δα-Δβ) for the singlet and triplet states, respectively, follow a consistent trend with the electron density distribution (**Figure S26**). Although torsional deviation is present between the donor and acceptor units, ESP plots reveal that there are strong intramolecular H-bonding and S···N interactions due to weak to moderate negative electron densities (yellow → orange) between the (β-CH···N and S···N) heteroatoms of the D–A units (**Figure S35**).

NICS calculations were employed to understand the nature of the ring current produced by the donor and acceptor units. Irrespective of π-extension, the silole ring of the donor shows strong anti-aromatic character with a mean $NICS_{iso(1)}$ value of +1.00 pm, an attribute of σ-π conjugation.[11] Our previous research demonstrated that an ideal anti-aromatic donor unit induces quinoidal characteristics in the central benzenoid ring of the acceptor unit, which is observed in this polymer once n = 6.[1, 11] Extension to the octamer (n = 8) unit demonstrated further aromatic-quinoidal

transformation with the $NICS_{iso(1)}$ value increasing from ~ - 7.0 to - 0.78 ppm (**Tables S4 – S8**). To further corroborate this, we carried out anisotropy of the induced current density (ACID) calculations on the oligomer series. In this method, the diatropic (clockwise) current indicates aromatic characteristics, and the paratropic (anticlockwise) current corresponds to anti-aromatic. The flow of diatropic current (red arrows) over the molecular framework demonstrates the existence of global aromaticity. However, the flow of paratropic current (blue arrows) inside the bridgehead silole ring for all oligomers is evidence of its strong quinoidal characteristics. **Figures S36-39** show a negligible to moderate amount of paratropic current at the central benzenoid ring of the acceptor for n = 1-6. However, a significant paratropic current is developed upon extending the π-conjugation to n = 8, corresponding to the transition of the ground spin-state configuration from singlet to triplet. These results are consistent with the measured $NICS_{iso(1)}$ values and the quinoidal bonding pattern observed in the BLA analysis.

3.10 Spin Delocalization. As demonstrated in section 3.9, the electronic and magnetic structure of the open-shell DA CP rapidly evolves as low molecular weight oligomers grow until the ground state triplet is stabilized (**Figure S22-S23**). At this point, further increases in molecular weight cause very minor changes to the electronic and magnetic properties. Polymers with target number average molecular weights of 15, 20, and 25 kg mol^{-1} were synthesized to demonstrate this point. The theoretical $|\Delta E_{ST}|$ changes by 16.581 kcal mol^{-1} from n = 1 – 8 (**Table S1**), whereas the experimental ΔE_{ST} changes by 0.0014 kcal mol^{-1} between 15 and 37 kg mol^{-1} (n ~15 – 37). The latter is a mere 0.008% of the initial change from n = 1 – 8, despite being three times the change in chain length. Similarly, the spin delocalization shows little change once the ground state triplet is stabilized. CW EPR spectra were recorded to examine the inhomogeneous broadening, which represents the limit of the hyperfine interaction and thus gives information on the relative degree of spin delocalization. Fitting the EPR spectra to a model accounting for a triplet with an isotropic J coupling gave similar inhomogeneous broadening values of 5.73, 5.56, and 5.89 G for the 15, 20, and 25 kg mol^{-1} samples, respectively, compared to the 5.57 G recorded for the 37 kg mol^{-1} polymer. These data indicate little change in the magnetic environment and spin delocalization across a ~20 kg mol^{-1} range.

4. Supporting Figures

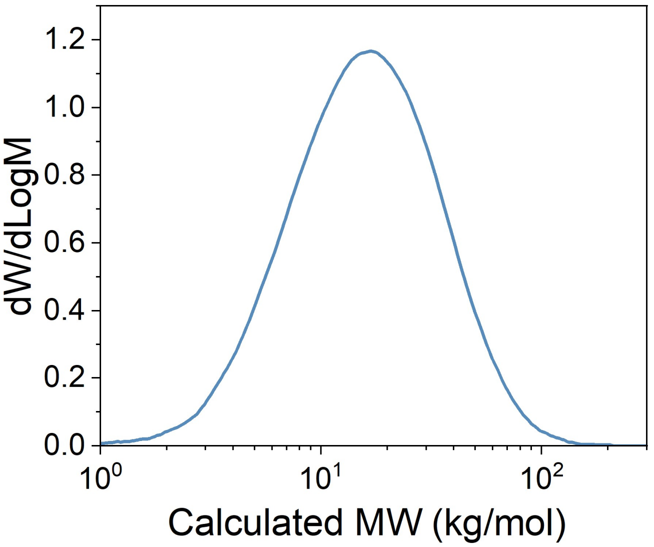

Figure S1. GPC trace obtained at 160°C in 1,2,4-trichlorobenzene.

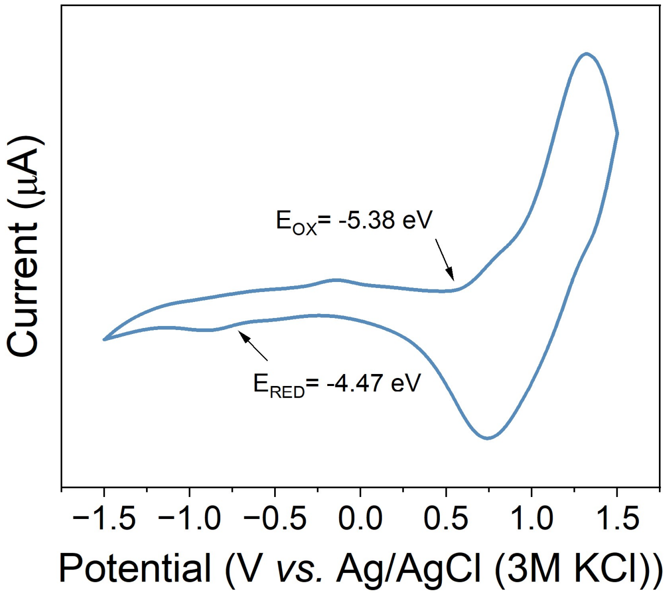

Figure S2. Cyclic voltammetry indicates the onset of oxidation and reduction potentials at -5.38 eV and -4.47 eV respectively.

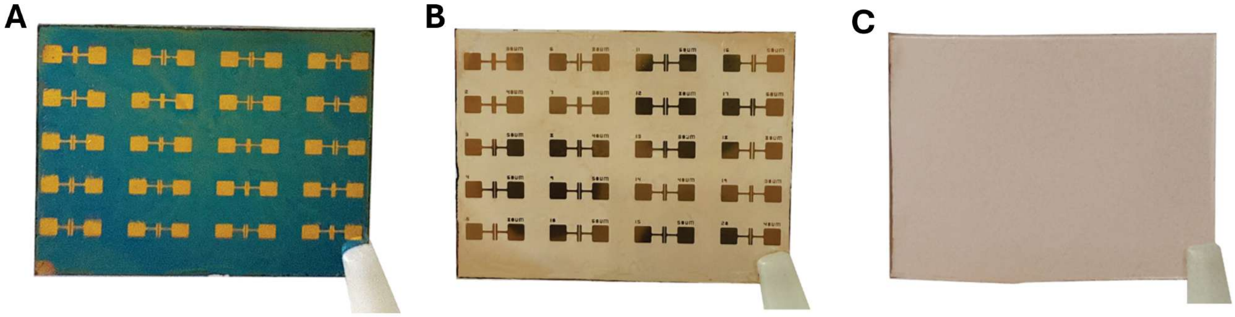

Figure S3. Thin films spin coated onto (**A**) silicon, (B) glass, (**C**) and plastic substrates.

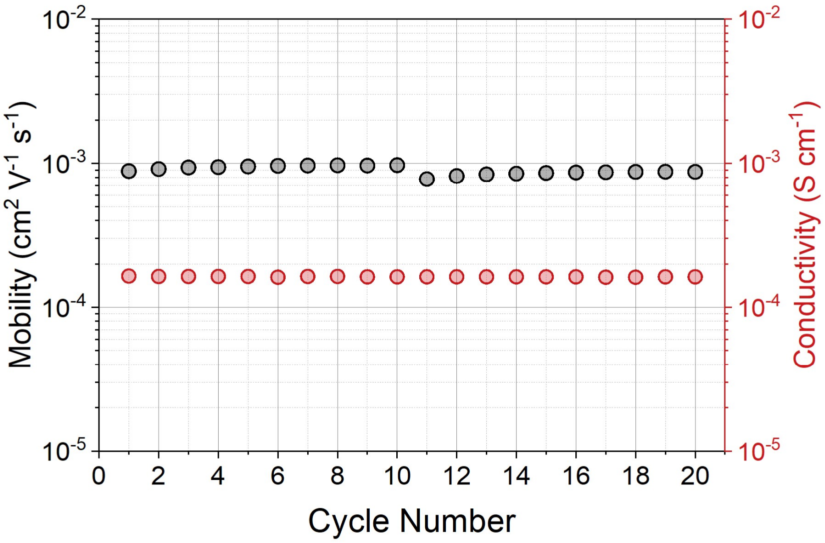

Figure S4. Field effect transistor output characteristics and current–voltage characteristics after repeated cycling.

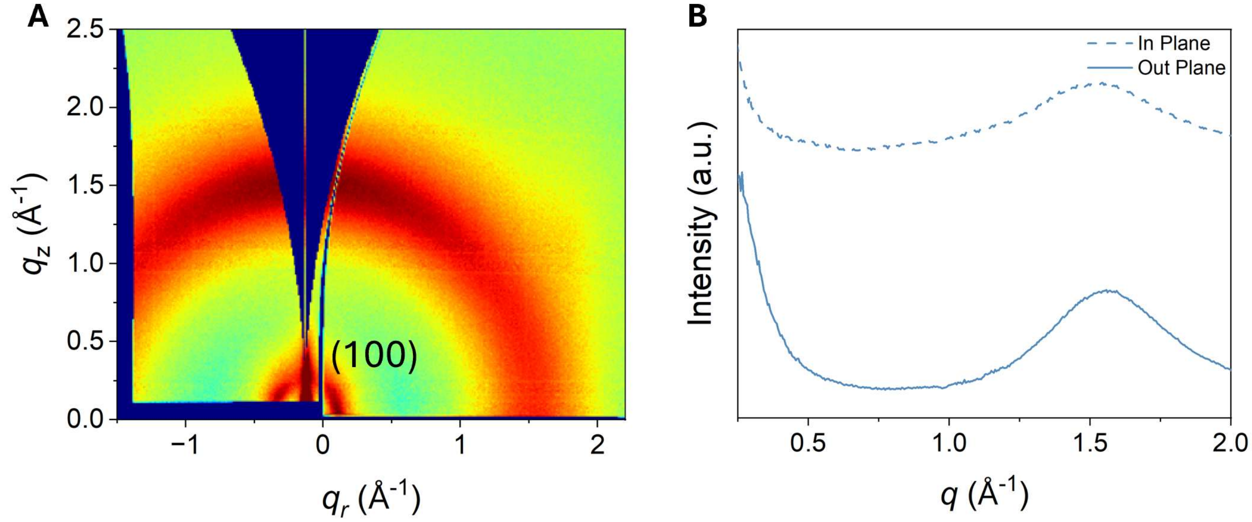

Figure S5. (**A**) Two-dimensional GIWAXS profile and (**B**) one-dimensional line cuts of the integrated in-plane and out-of-plane GIWAXS profiles of the Si-bridgehead polymer thin film spin-coated from chlorobenzene and thermally annealed at 110 °C for 15 minutes.

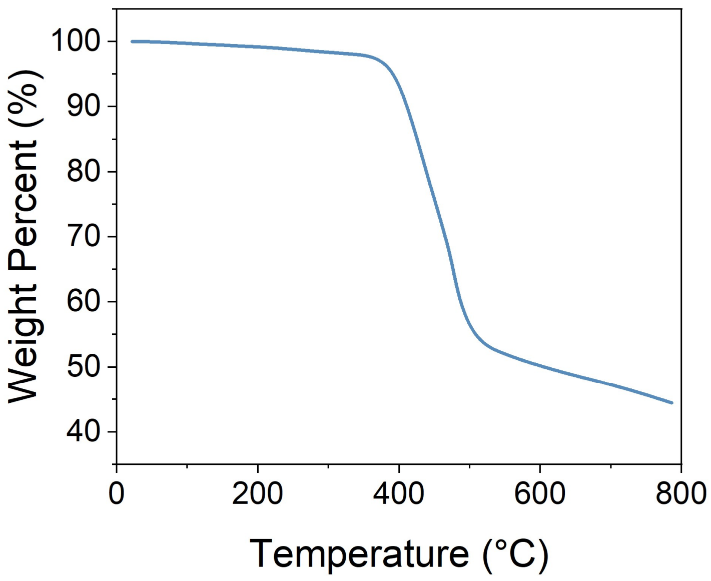

Figure S6. TGA curve of a pristine polymer powder; thermal decomposition begins at ~390 °C.

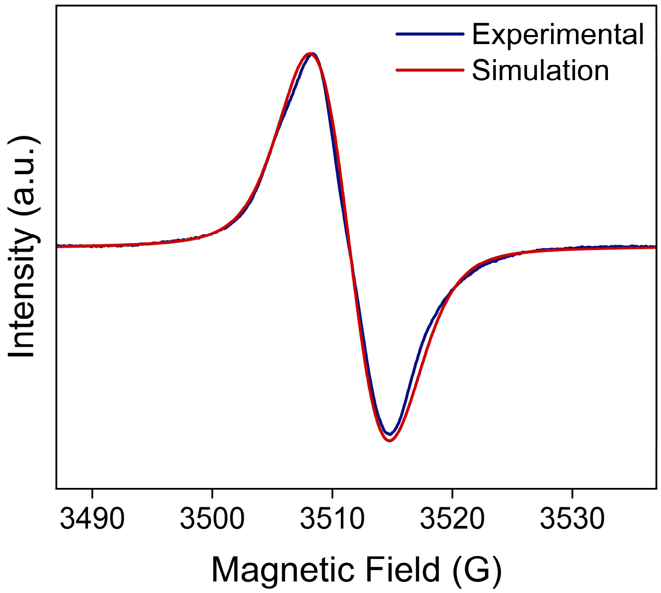

Figure S7. Room temperature CW-EPR of the powder and simulation.

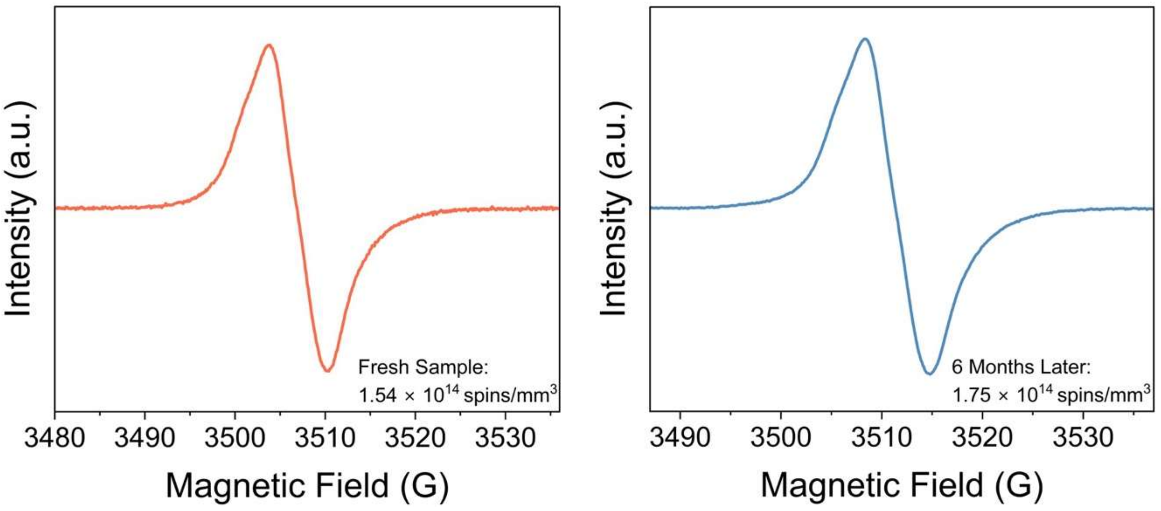

Figure S8. Room temperature CW-EPR spectra of the polymer sample with the spin count quantified against a DPPH standard: A) fresh sample, B) sample stored for 6 months at room temperature. The negligibly higher spin concentration for the aged sample can be attributed to positioning differences of the sample in the resonator and thermal fluctuations.

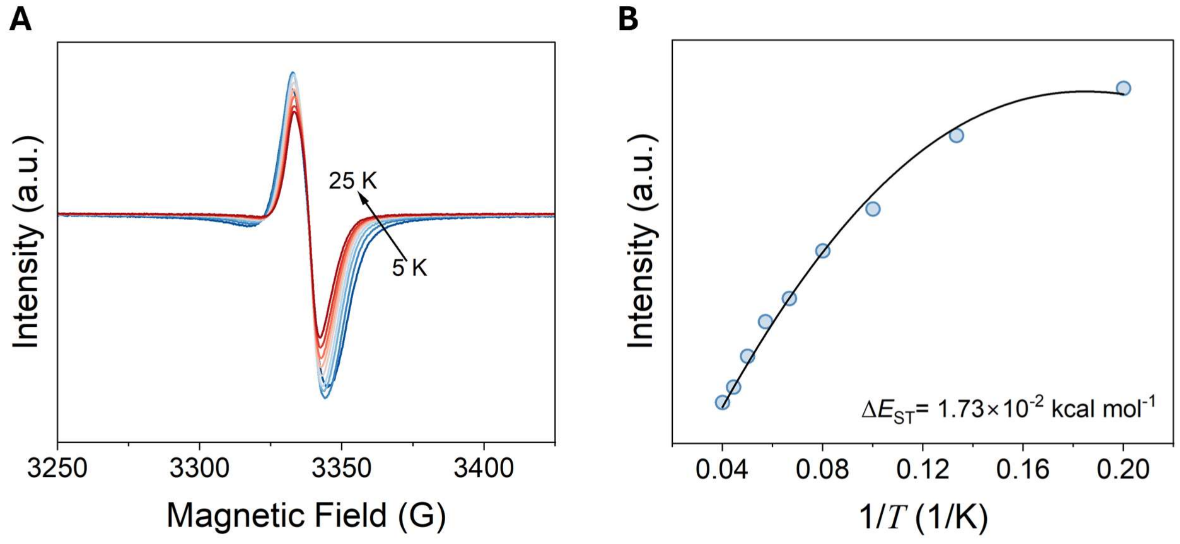

Figure S9. (**A**) VT-EPR spectra from 5 – 25 K and (**B**) temperature-dependent fit to the Bleaney-Bowers equation giving a ΔE_{ST} of 1.73×10^{-2} kcal mol^{-1}.

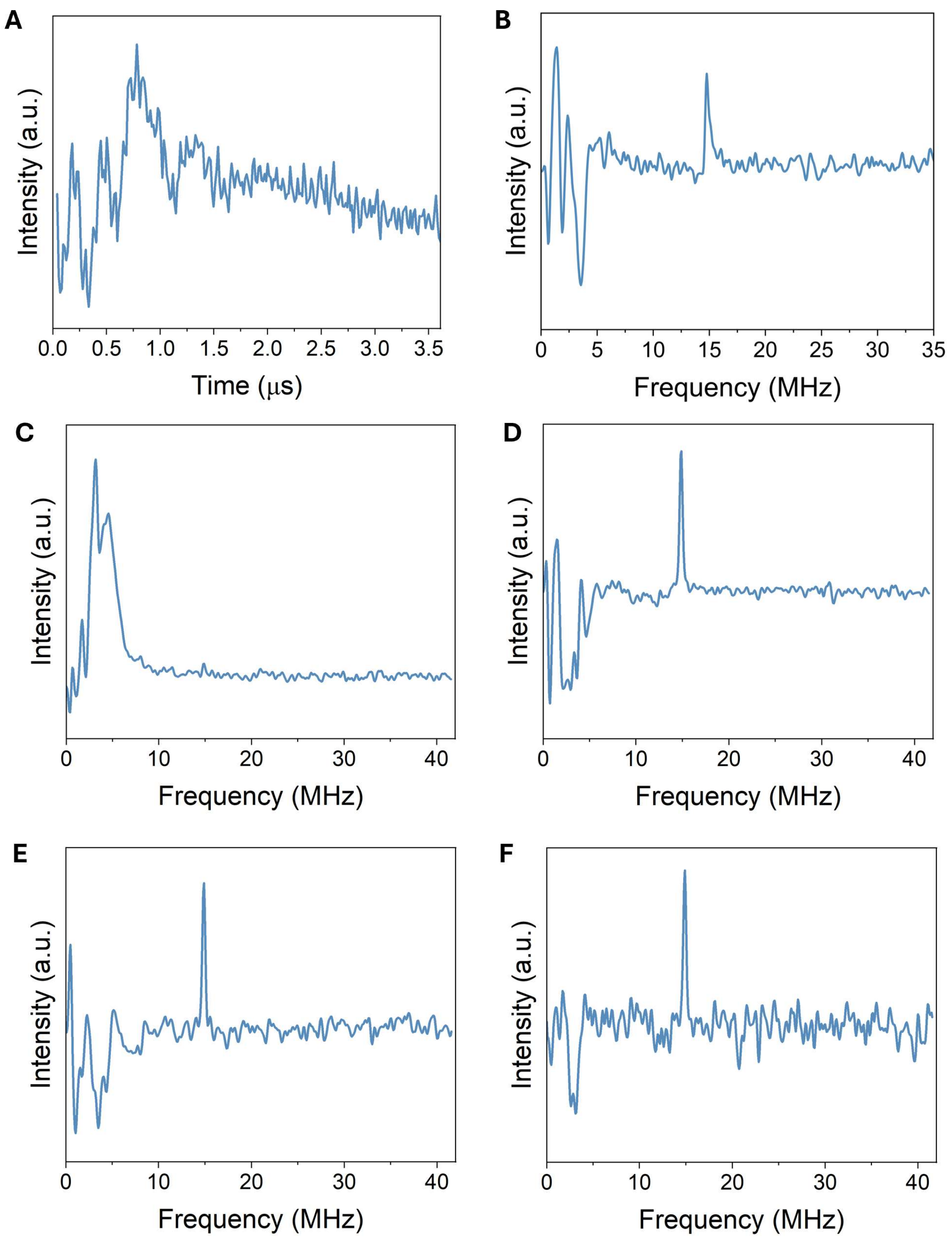

Figure S10. (**A**) Time domain 3-pulse ESEEM data and (**B**) Fourier transformed 3-pulse ESEEM data taken at 85 K and $\tau = 240$ ns with characteristic peaks appearing between 0 MHz and 4 MHz corresponding to ^{14}N hyperfine coupling and at 15 MHz corresponding to ^{1}H hyperfine coupling. Additional 3-pulse ESEEM spectra collected at τ values of (**C**) 128 ns, (**D**) 368 ns, (**E**) 560 ns, and (**F**) 968 ns.

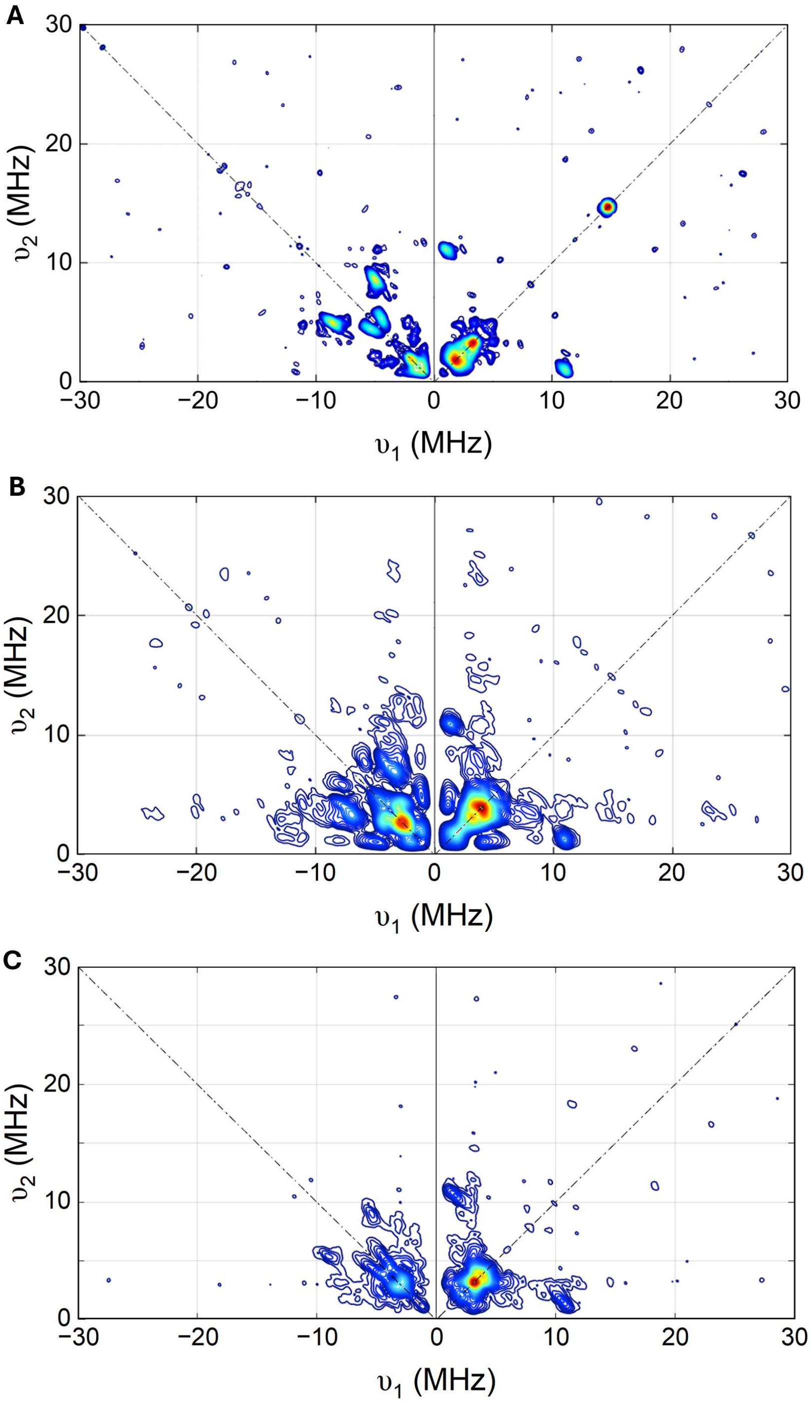

Figure S11. (**A**) HYSCORE data at 5.5 K and τ = 300 ns with weakly and strongly coupled ^{14}N peaks and a weakly coupled ^{1}H peak, along with spectra collected at (**B**) 200 ns and (**C**) 128 ns.

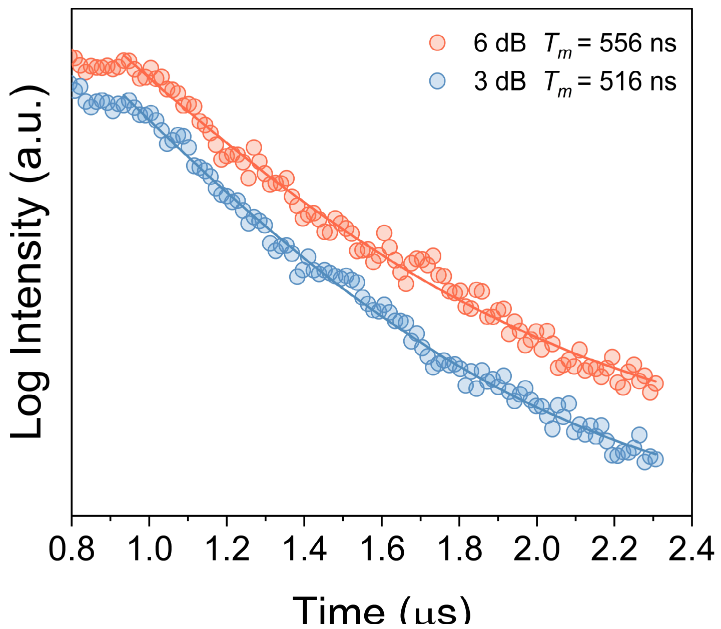

Figure S12. Hahn echo decays taken at 85 K and different microwave power attenuations fit to an exponential decay. The higher attenuation gives a longer T_m value (556 ns versus 516 ns), which is an indication of instantaneous diffusion.

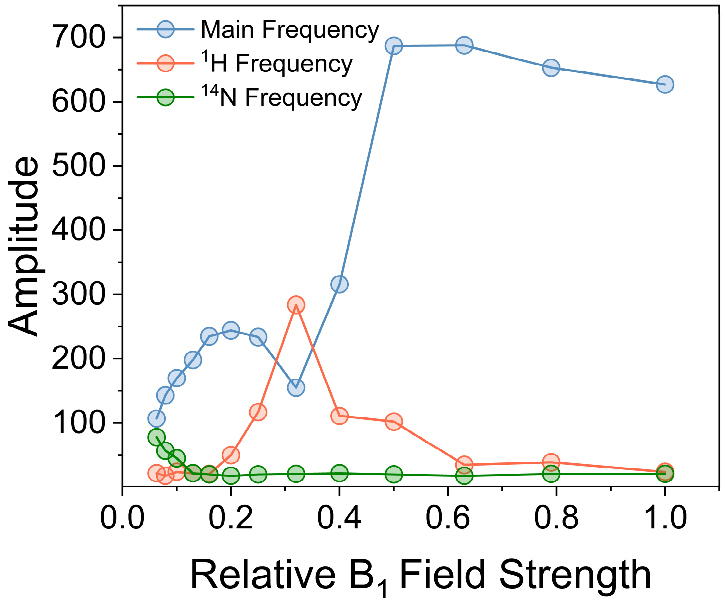

Figure S13. The amplitude of the main signal (recorded at the position of max intensity), ^{1}H (recorded at 15 MHz), and ^{14}N (recorded at 3 MHz) rabi frequencies versus the relative amplitude of the B_1 field (relative to the maximum amplitude at 0 dB microwave attenuation), illustrating a sharp decline in the amplitude of the main signal at weaker B_1 field strengths (beginning at ~0.45 relative B_1 which equals a rabi frequency of ~20 MHz) and frequencies corresponding to the nuclear Zeeman frequency of coupled nuclei.

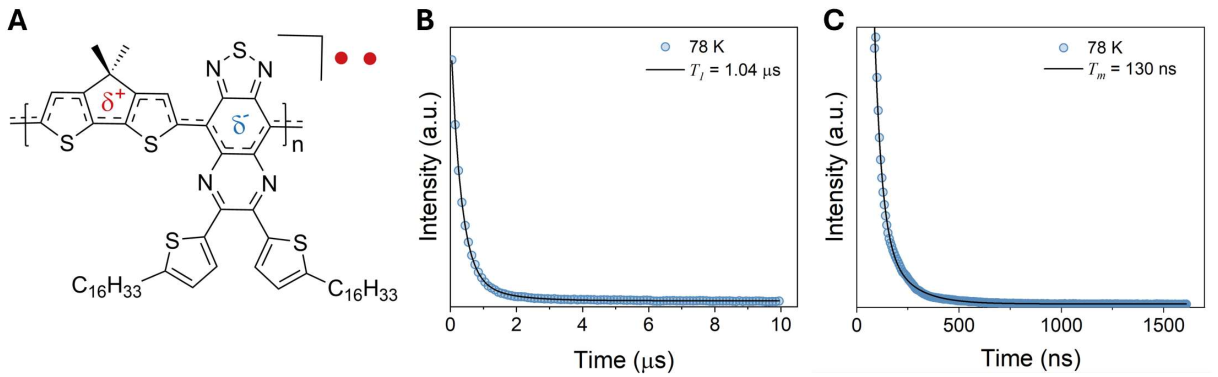

Figure S14. (**A**) Chemical structure of the carbon bridgehead polymer. (**B**) Inversion recovery fit to an exponential decay to give T_1 and (**C**) Hahn echo decay fit to an exponential decay to give T_m.

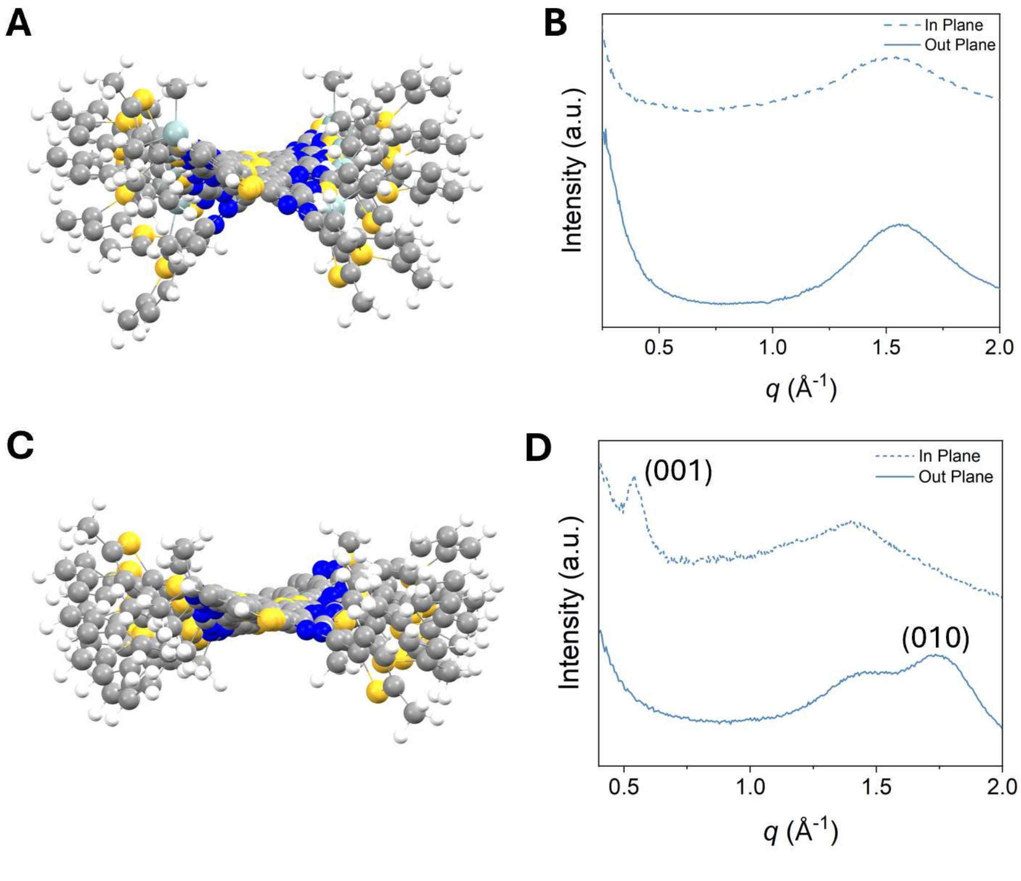

Figure S15. Comparison of the backbone topological structures upon bridgehead substitution. (**A**) Si-bridgehead backbone and (**B**) one-dimensional line cuts of the integrated in-plane and out-of-plane GIWAXS profiles showing a broad peak centered at q~1.5 Å^{-1} corresponding to amorphous scattering. (**C**) C-bridgehead backbone topology and (**D**) one-dimensional line cuts of the integrated in-plane and out-of-plane GIWAXS profiles reveal a distinct out-of-plane peak at q~1.75 Å^{-1} corresponding to π-π stacking.

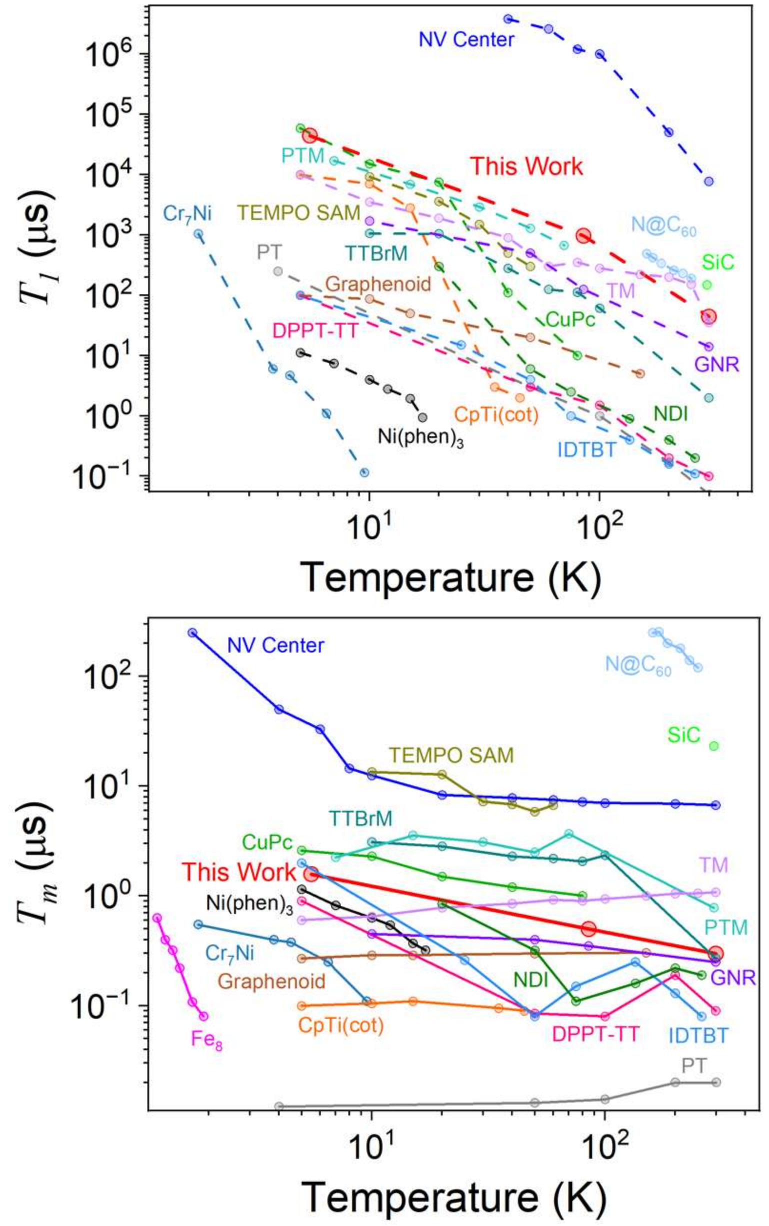

Figure S16. Comparison of this DA CP with other synthetic qubit systems. Data are taken from Fe_8 molecular magnets (Fe_8),[17] Cr_7Ni molecular magnet (Cr_7Ni),[18] (η^8-cyclooctatetraene)(η^5-cyclopentadienyl)titanium (CpTi(cot)),[19] diindeno-fused bischrysene (graphenoids),[12] tris(1,10-phenanthroline-κ^2 *N,N'*) nickel(II) tetrafluoroborate ($Ni(phen)_3$),[20] bis(1,3-dithiole-2-thione-4,5-dithiolate)oxovanadium(IV) tetraphenylphosphonium ($VO(dmit)_2$),[21] copper phthalocyanine (CuPc),[22] nitrogen atom endohedral fullerene ($N@C_{60}$),[23] nitrogen-vacancy center in diamond (NV Center),[24] tris(2,4,6-tribromophenyl)methyl radical (TTBrM),[25] perchlorotriphenylmethyl radical (PTM),[26] tris(3,4,5-trichlorophenyl)methyl radical (TM),[27] 2,2,6,6-tetramethylpiperidine-1-oxyl self-assembled monolayers (TEMPO SAM),[28] magnetic edge states in graphene nanoribbons (GNR),[29] carriers in poly(diketopyrrolopyrrole-*co*-thiophene-thieno[3,2-*b*]thiophene) (DPPT-TT),[30] carriers in polythiophene doped with BF_4^- (PT),[31] carriers in poly(indacenodithiophene-*co*-benzothiadiazole) (IDTBT),[32] carriers in poly(naphthalenediimide-*co*-bithiophene) (NDI),[32] and paramagnetic divacancies in SiC.[33] See **Table S14** for measurement parameters and sample conditions.

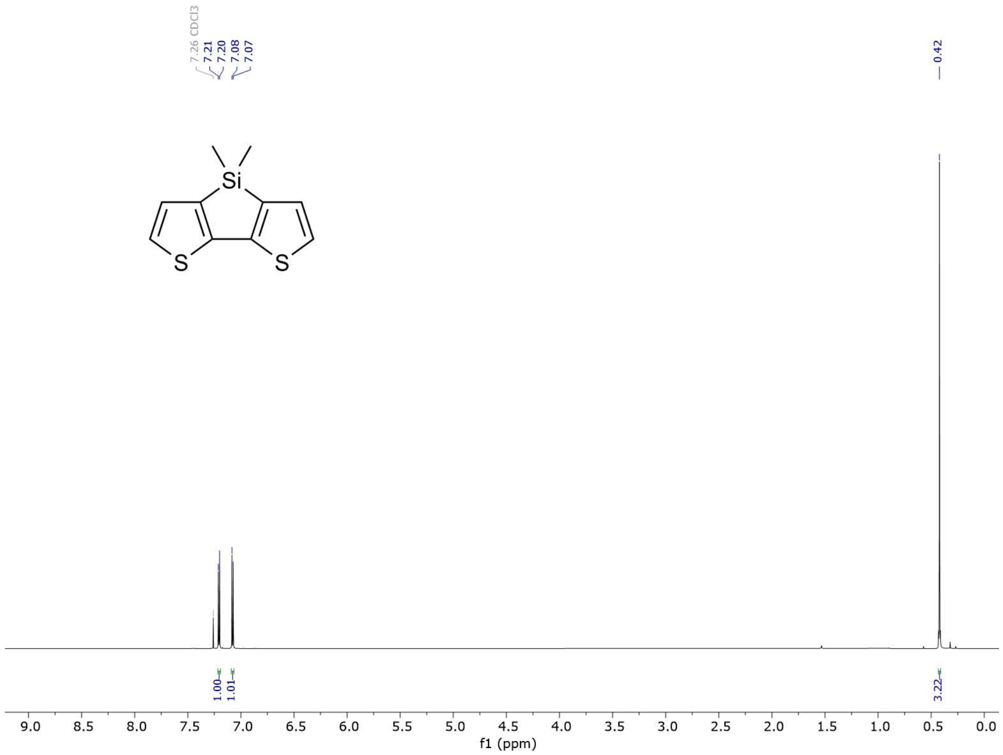

Figure S17. ^{1}H NMR spectrum (400 MHz, chloroform-*d*) of 4,4-dimethyl-4*H*-silolo[3,2-*b*:4,5-*b*']dithiophene.

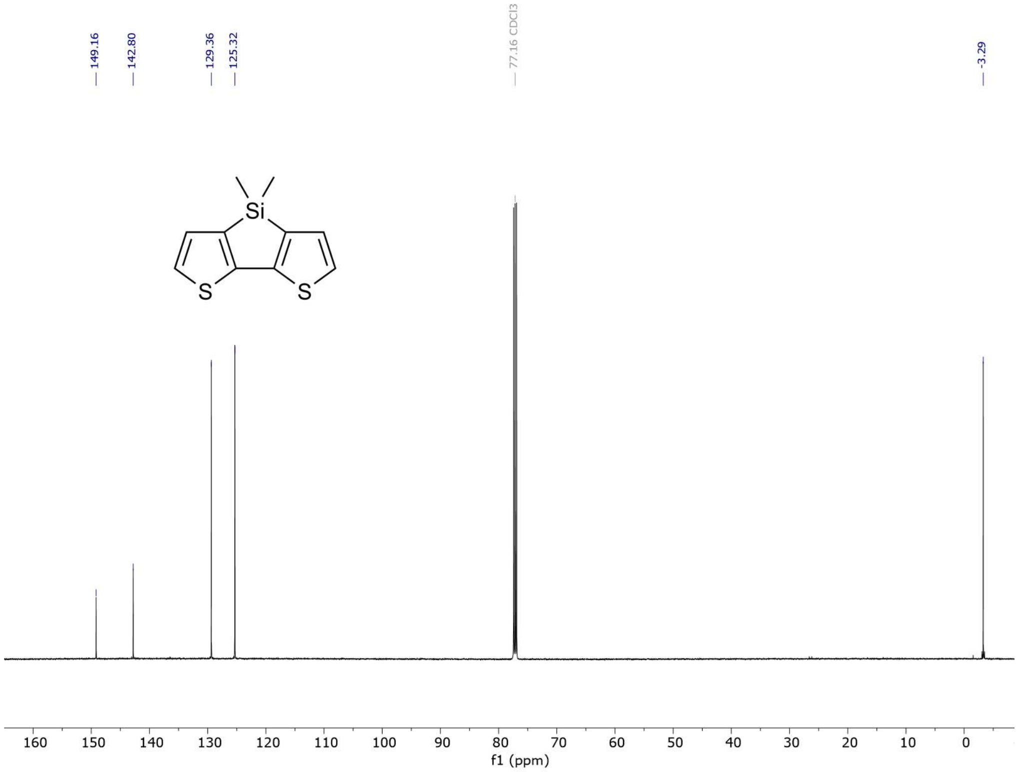

Figure S18. ^{13}C NMR spectrum (126 MHz, chloroform-*d*) of 4,4-dimethyl-4*H*-silolo[3,2-*b*:4,5-*b*']dithiophene.

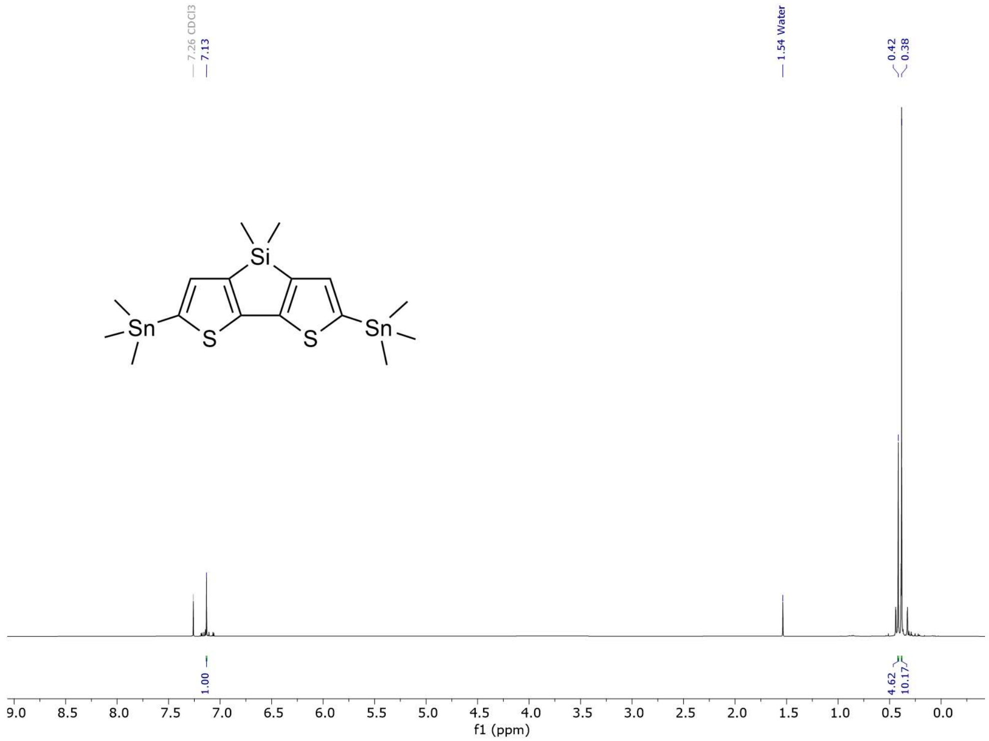

Figure S19. ^{1}H NMR spectrum (500 MHz, chloroform-*d*) of 4,4-dimethyl-2,6-bis(trimethylstannyl)-4*H*-silolo[3,2-*b*:4,5-*b*']dithiophene.

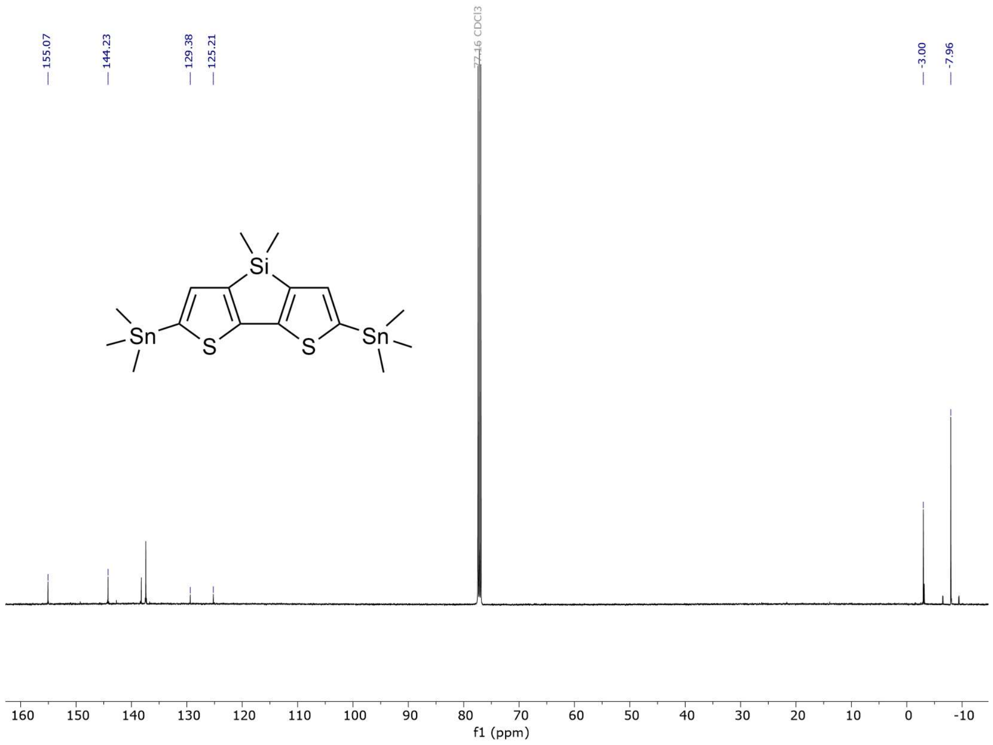

Figure S20. ^{13}C NMR spectrum (126 MHz, chloroform-d) of 4,4-dimethyl-2,6-bis(trimethylstannyl)-4*H*-silolo[3,2-*b*:4,5-*b*']dithiophene.

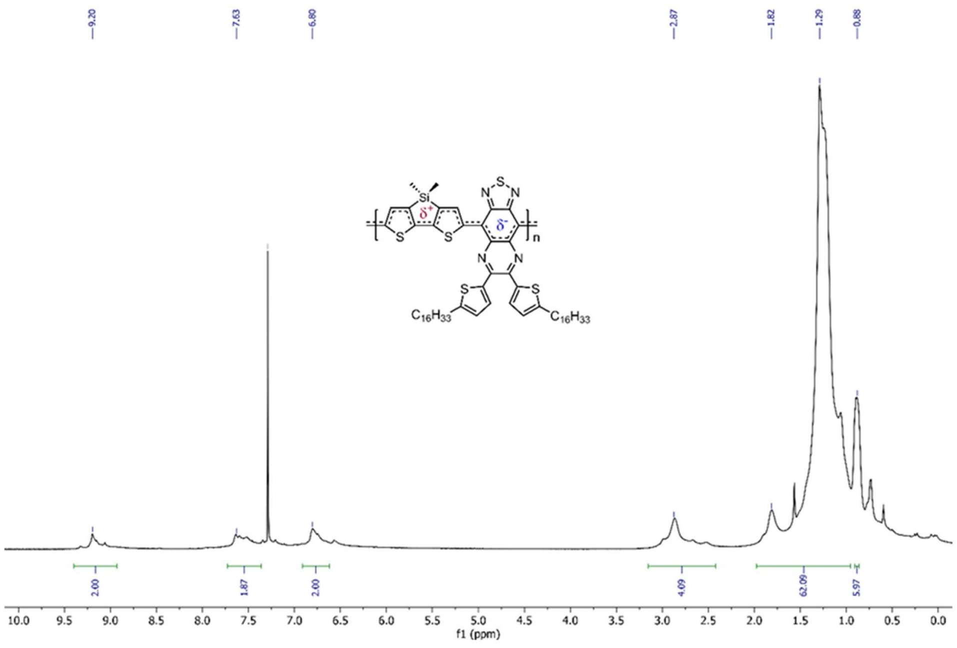

Figure S21. ^{1}H NMR spectrum of poly[4-(4,4-dimethyl-4*H*-silolo[3,2-*b*:4,5-*b*']dithiophene)-*alt*-6,7-bis(5-hexadecyl-thiophen-2-yl)-[1,2,5]thiadiazolo[3,4-*g*]quinoxaline] (600 MHz, Tetrachloroethane-d_2, 398 K).

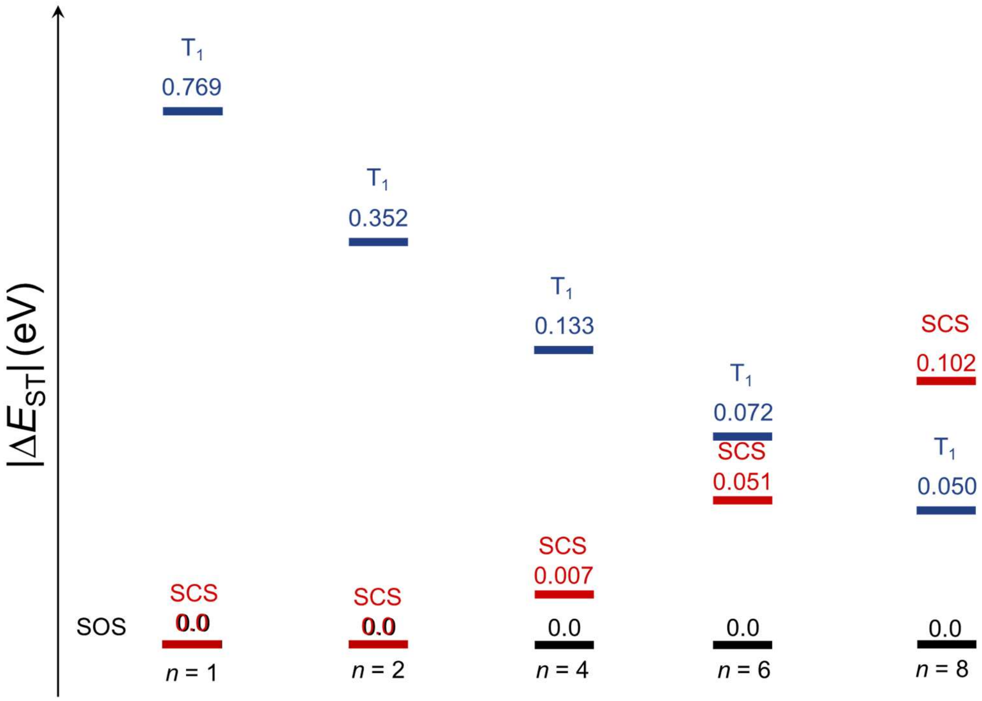

Figure S22. Absolute value of ΔE_{ST} of the as a function of *n* = 1, 2, 4, 6, and 8 repeat units showing the change in relative energies of the various spin states with reference to singlet open-shell (SOS) state, triplet state (T_1), and singlet closed shell (SCS).

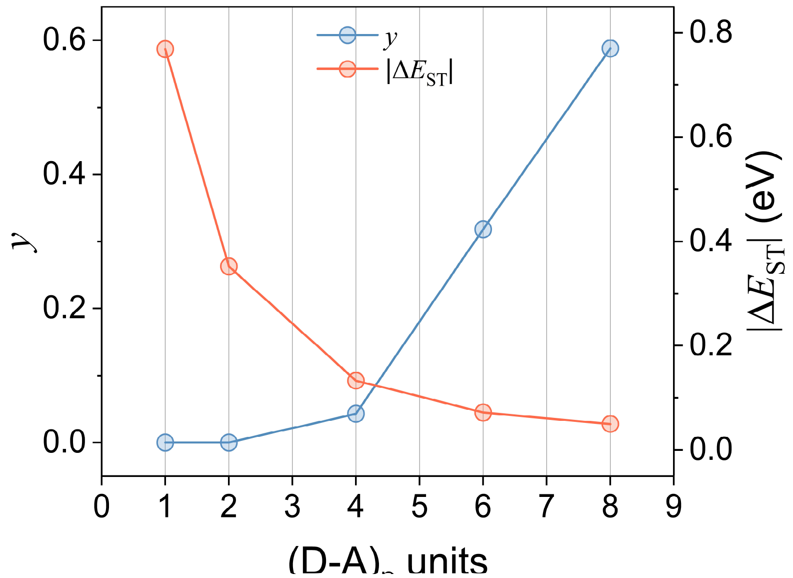

Figure S23. Diradical character index (y) plotted against the absolute value of $|\Delta E_{ST}|$ for the oligomers with n = 1-8 repeat units.

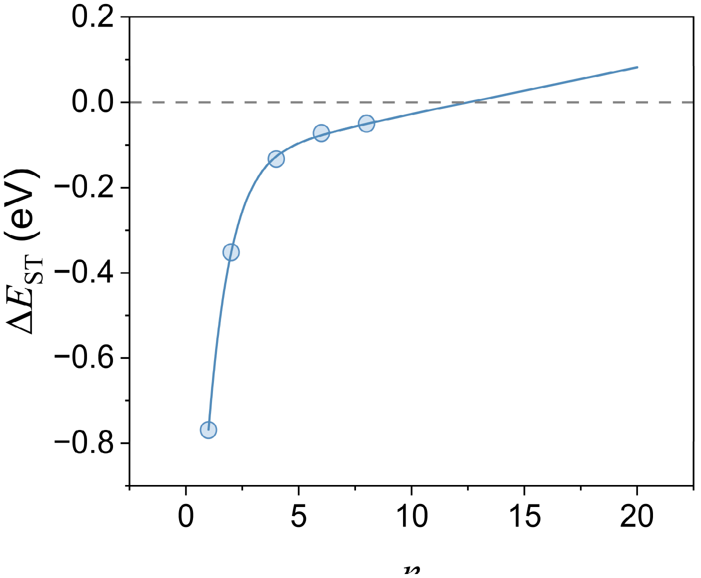

Figure S24. Singlet – triplet energy gap (ΔE_{ST}) of the oligomers. As n increases, E_{ST} increases rapidly and reaches a value of -0.05 eV for n = 8. Extrapolation of these data indicates that an inflection point is achieved at n ~ 13 (dotted line), corresponding to an M_w of ~13.6 kg mol^{-1}.

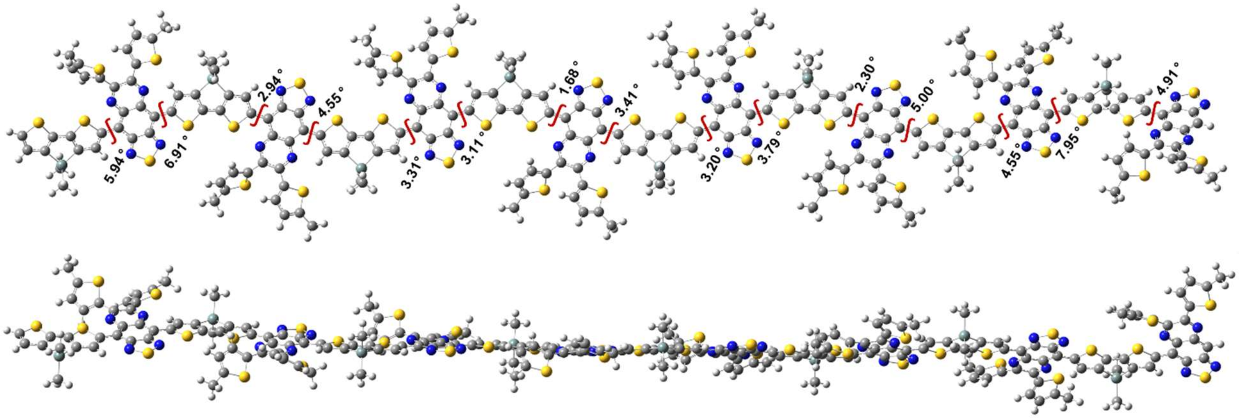

Figure S25. Triplet-optimized geometries of the octamer (n = 8) with selected dihedral angles and the enhanced planarity of the π-framework can be seen from the side perspective.

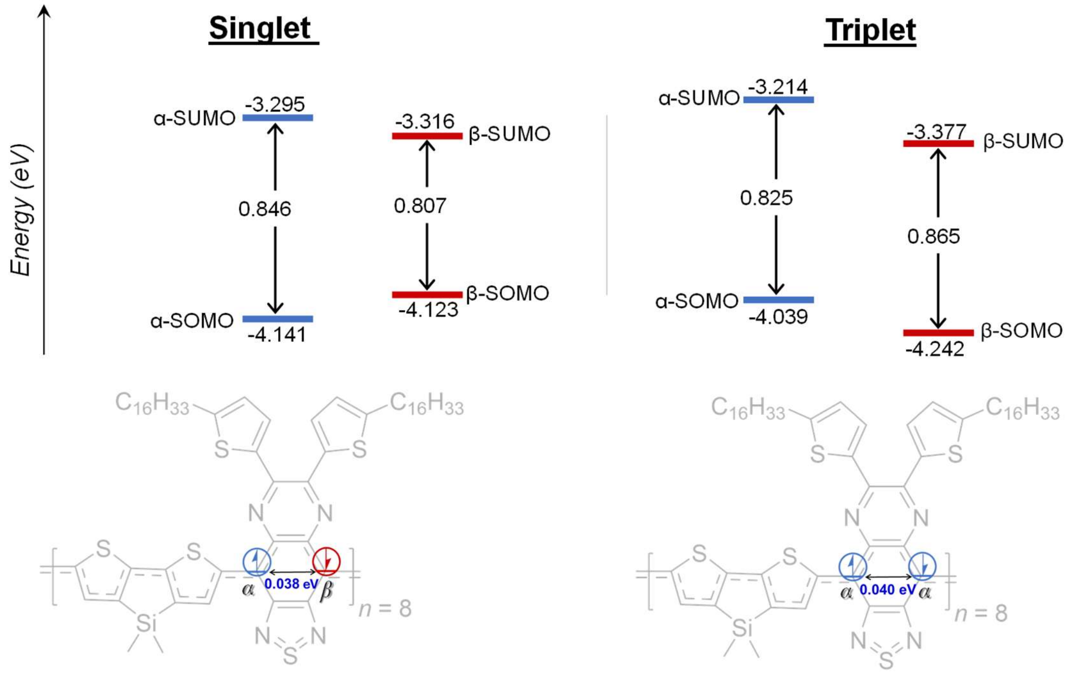

Figure S26. Schematic diagram illustrates the degree of degeneracy between two spin (α and β) states corresponding to the optimized octamer geometry at the singlet and triplet ground state configuration.

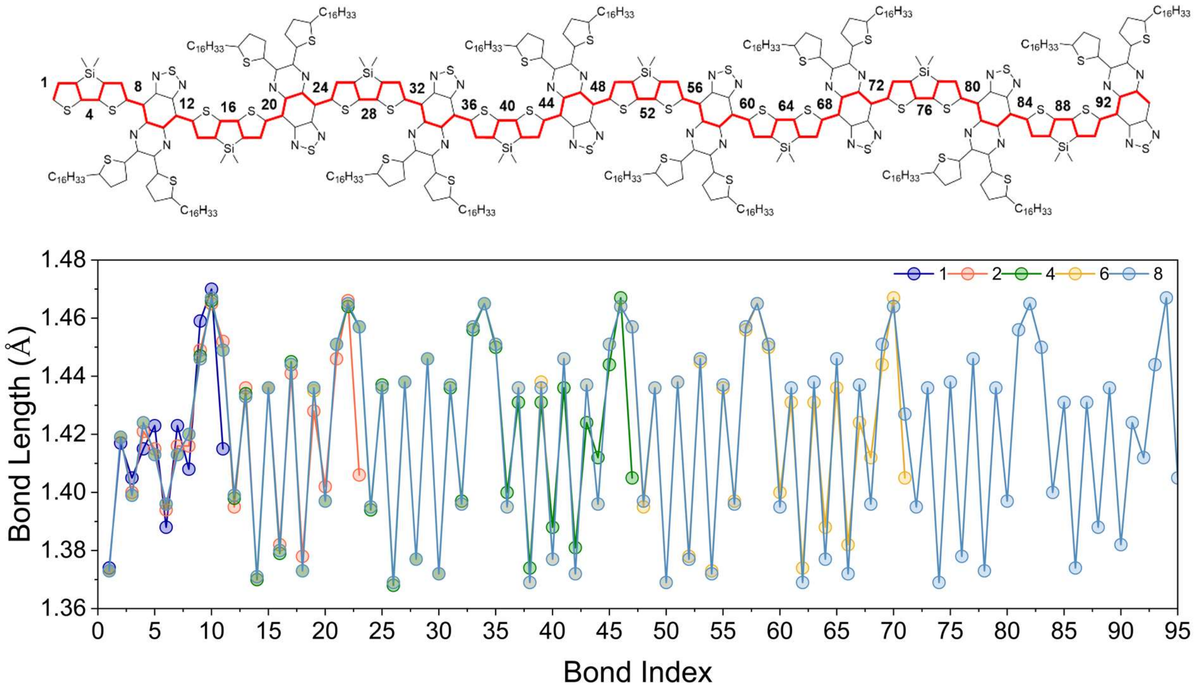

Figure S27. Bond index plots of the optimized geometries for n = 1, 2, 4, 6, and 8 repeat units.

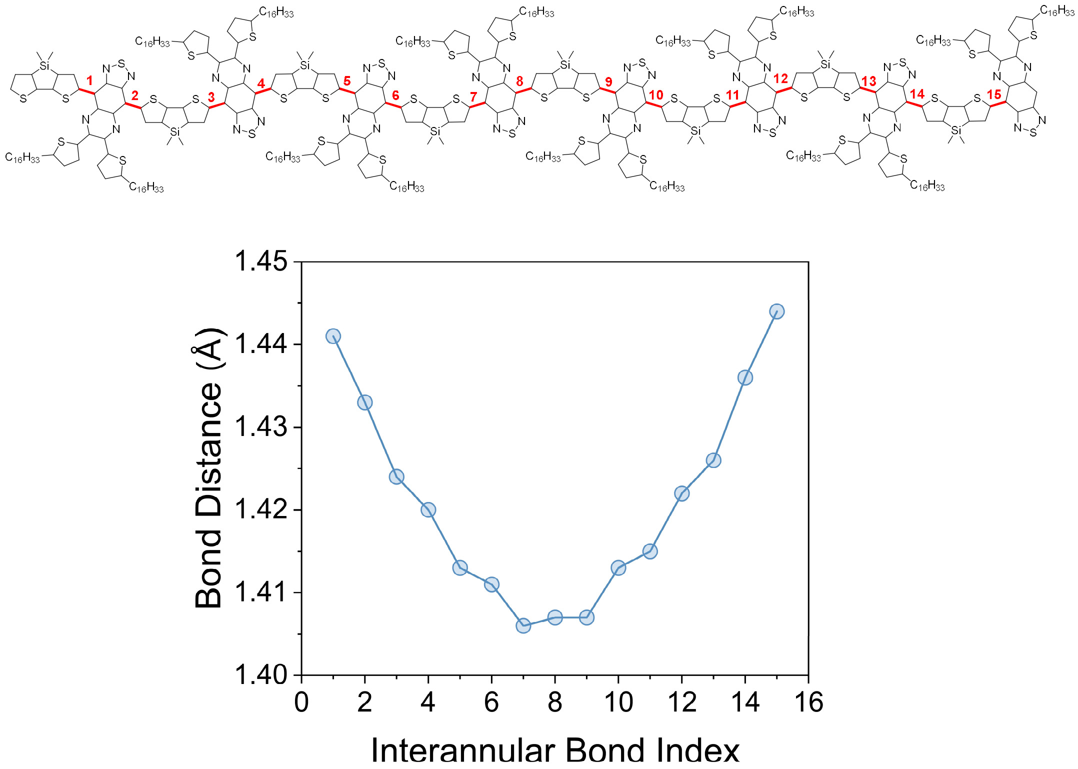

Figure S28. Interannular bond distance between the donor and acceptor segments from the optimized geometries of the n = 8 oligomer.

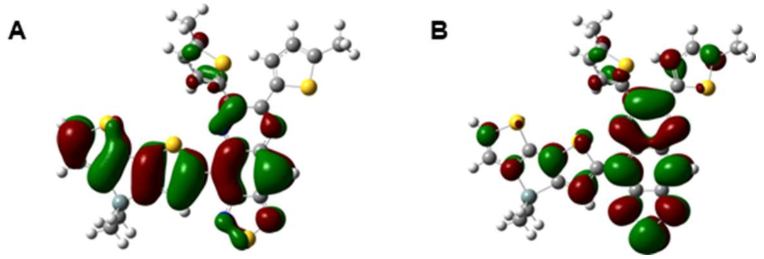

Figure S29. Optimized closed-shell ground state geometric structures for the single (n = 1) D-A unit and pictorial representations of the frontier MOs. (A) HOMO and (B) LUMO. The green and red surfaces represent positive and negative signs of the MO at isovalue = 0.02 au, respectively. Color codes for the atoms are: gray for C, blue for N, yellow for S, and mint for Si.

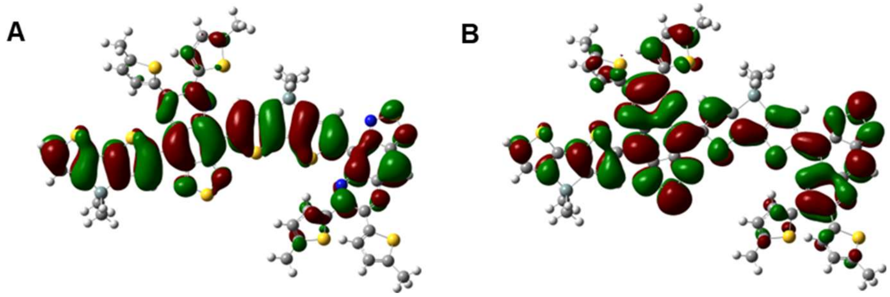

Figure S30. Optimized closed-shell ground state geometric structures for the dimer (n = 2) and pictorial representations of the frontier MOs. (A) HOMO and (B) LUMO. The green and red surfaces represent positive and negative signs of the MO at isovalue = 0.02 au, respectively. Color codes for the atoms are gray for C, blue for N, yellow for S, and mint for Si.

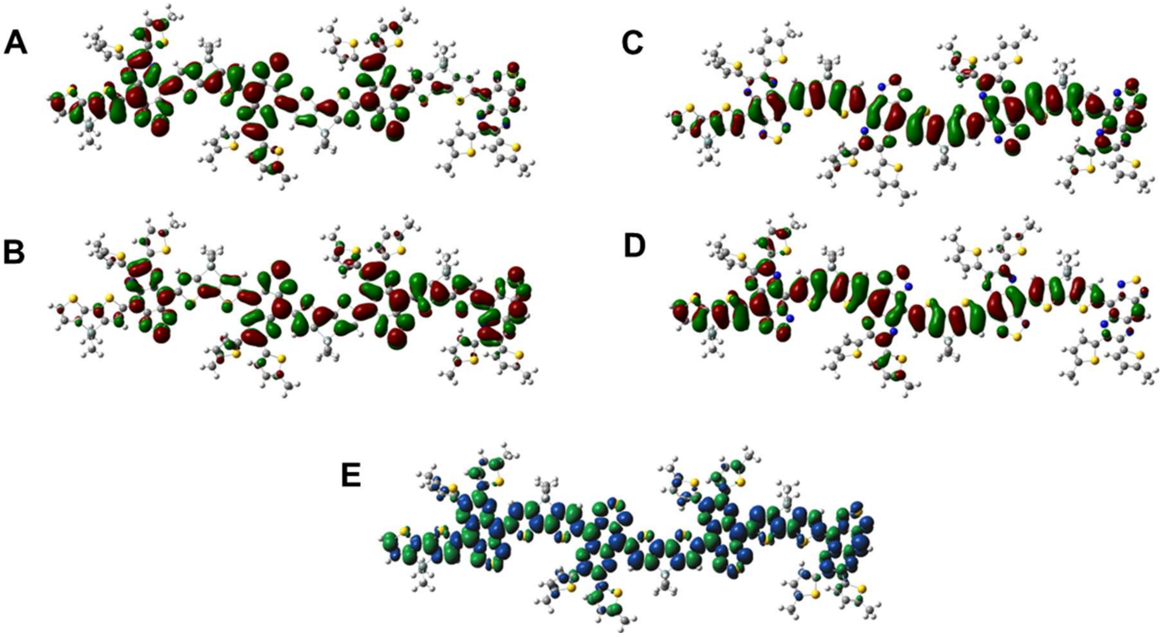

Figure S31. Optimized open-shell ground state geometric structures for the tetramer (n = 4) and pictorial representations of the frontier MOs and spin density distribution. (a) α-SUMO and (b) β-SUMO, (c) α-SOMO and (d) β-SOMO, and (e) Spin density distribution of the open-shell singlet. The green and red surfaces represent positive and negative signs of the MO at isovalue = 0.02 au, respectively. The blue and green surfaces represent positive and negative contributions of the spin density at an isovalue = 0.0004 au. Color codes for the atoms are gray for C, blue for N, yellow for S, and mint for Si.

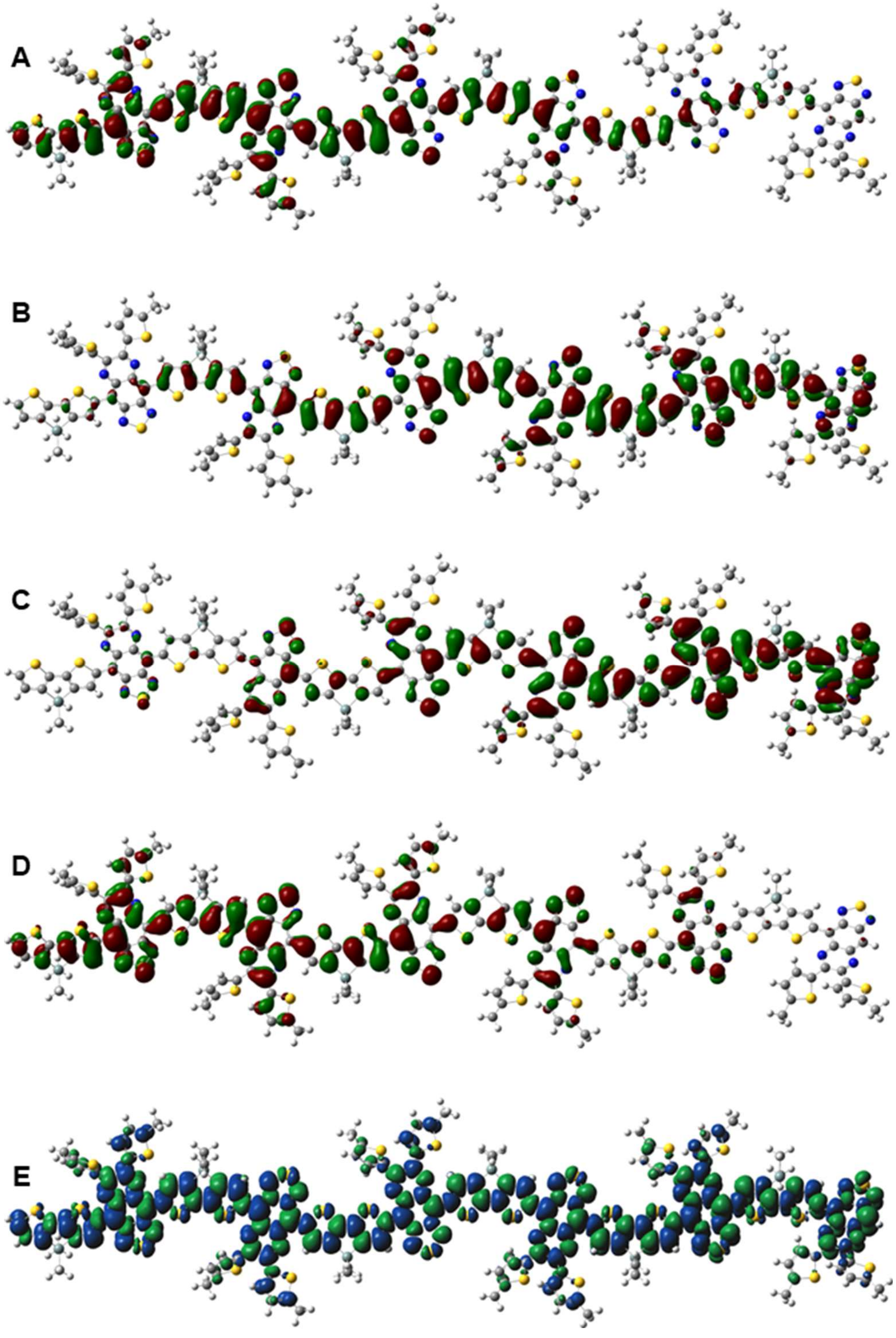

Figure S32. Optimized open-shell ground state geometric structures for the hexamer (n = 6) and pictorial representations of the frontier MOs and spin density distribution. (a) α-SUMO and (b) β-SUMO, (c) α-SOMO and (d**)** β-SOMO, and (e) Spin density distribution of the open-shell singlet. The green and red surfaces represent positive and negative signs of the MO at isovalue = 0.02 au, respectively. The blue and green surfaces represent positive and negative contributions of the spin density at an isovalue = 0.0004 au. Color codes for the atoms are gray for C, blue for N, yellow for S, and mint for Si.

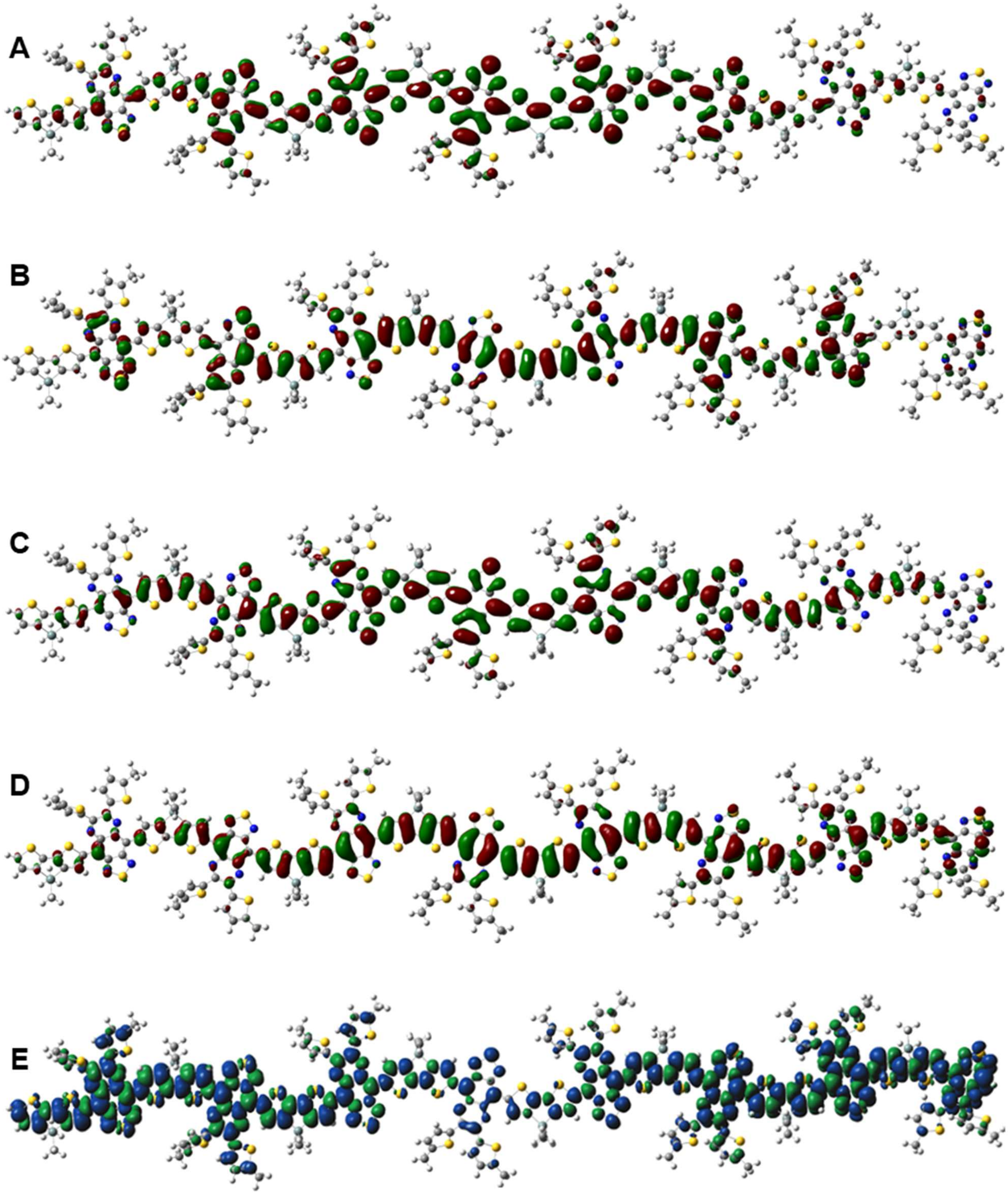

Figure S33. Optimized open-shell ground state geometric structures for the octamer (n = 8) and pictorial representations of the frontier MOs and spin density distribution. (a) α-SUMO and (b) β-SUMO, (c) α-SOMO and (d) β-SOMO, and (e) Spin density distribution of the open-shell triplet. The green and red surfaces represent positive and negative signs of the MO at isovalue = 0.02 au, respectively. The blue and green surfaces represent positive and negative contributions of the spin density at an isovalue = 0.0004 au. Color codes for the atoms are gray for C, blue for N, yellow for S, and mint for Si.

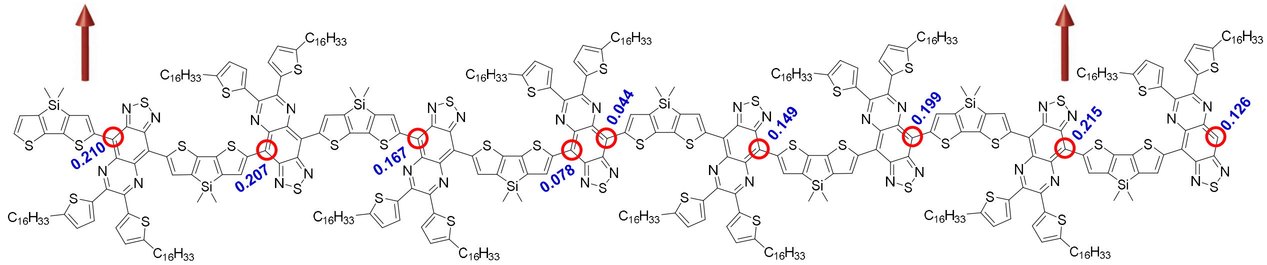

Figure S34. Red circles mark the spin centers with maximum spin density coefficients, highlighting the localization of unpaired electrons at the terminal repeat units in the *n* = 8 triplet ground state geometry.

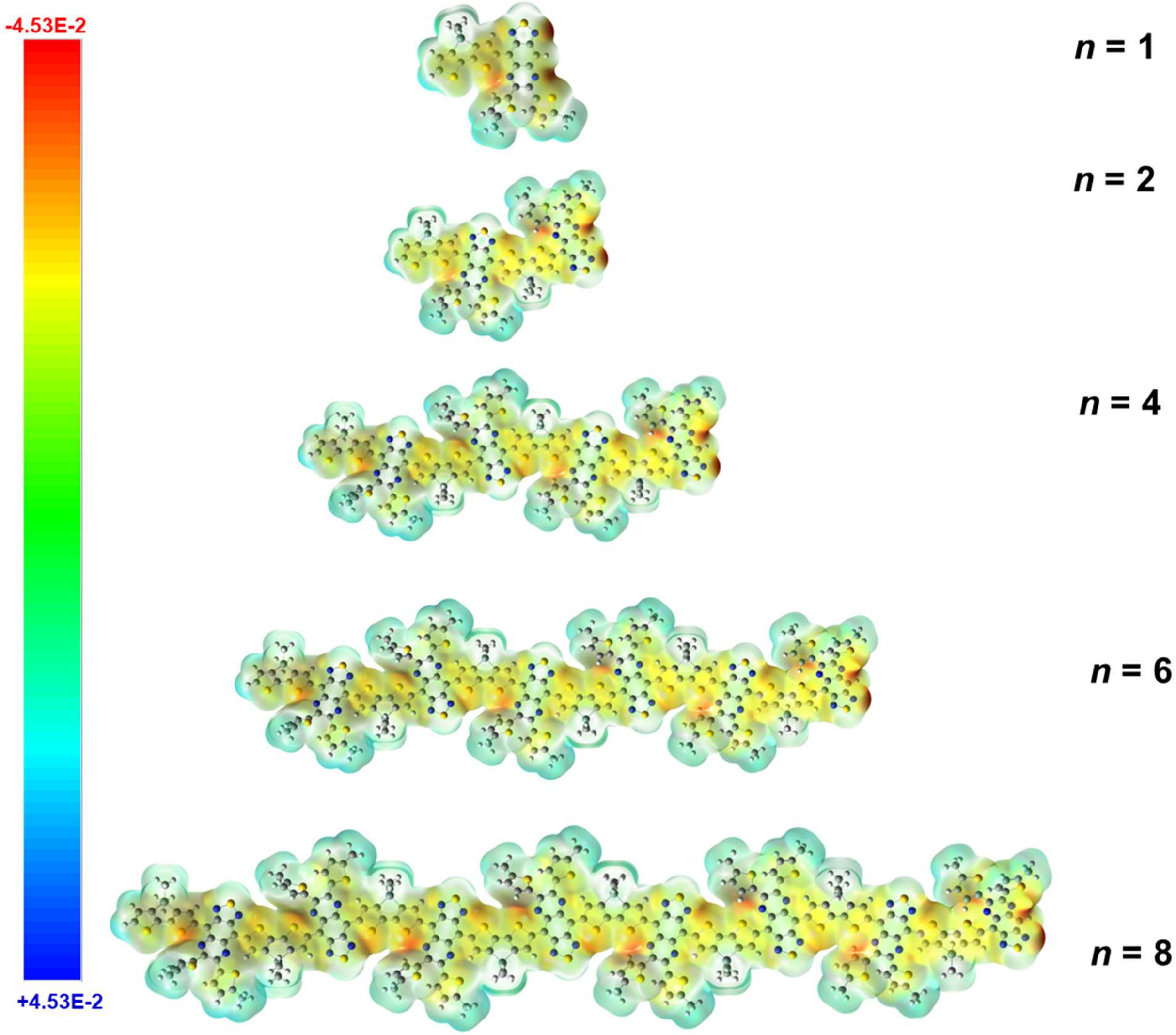

Figure S35. Molecular electrostatic potential (MESP) surface plots of the optimized oligomers with *n* = 1, 2, 4, 6, and 8 repeat units for the polymer reinforce the existence of intramolecular H-bonding and S···N interactions through a weak to moderate negative electrostatic environment between the D-A units**.**

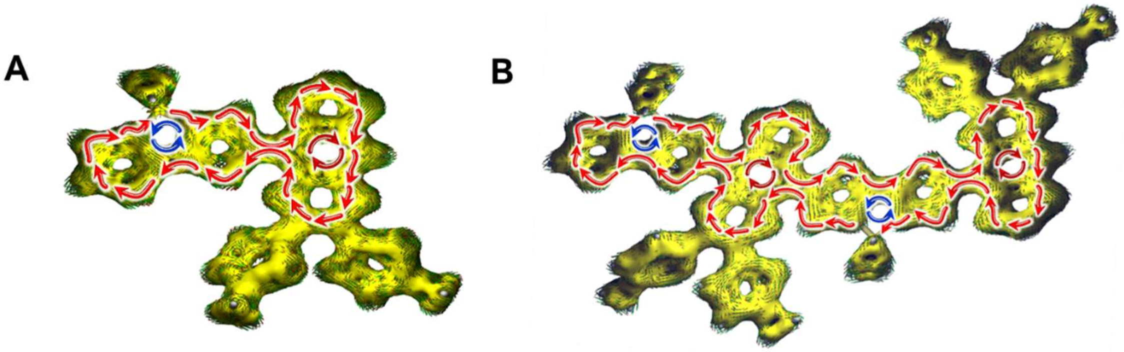

Figure S36. Anisotropy of the induced current density (ACID) of the π-system at CSGT-UCAMB3LYP/6-31G** level of theory for (A) single repeat unit (n = 1) and (B) dimer unit (n = 2). The current density vectors plotted on the ACID isosurface indicate ring current (clockwise, counterclockwise) and prevalent delocalization pathways present in these systems.

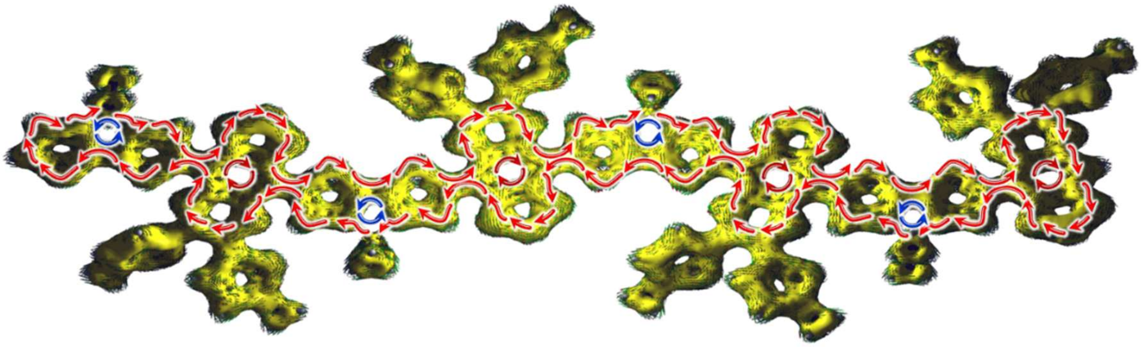

Figure S37. Anisotropy of the induced current density (ACID) of the π-system at CSGT-UCAMB3LYP/6-31G** level of theory for the tetramer unit (n = 4). The current density vectors plotted on the ACID isosurface indicate ring current (clockwise, counterclockwise) and prevalent delocalization pathways present in these systems.

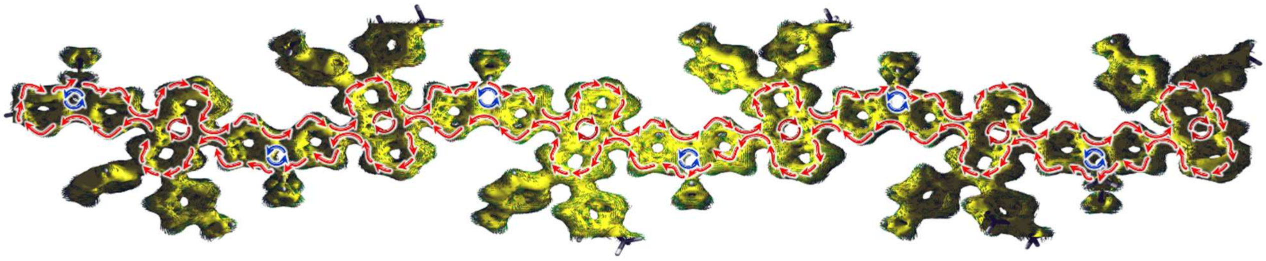

Figure S38. Anisotropy of the induced current density (ACID) of the π-system at CSGT-UCAMB3LYP/6-31G** level of theory for the hexamer unit (n = 6). The current density vectors plotted on the ACID isosurface indicate ring current (clockwise, counterclockwise) and prevalent delocalization pathways present in these systems.

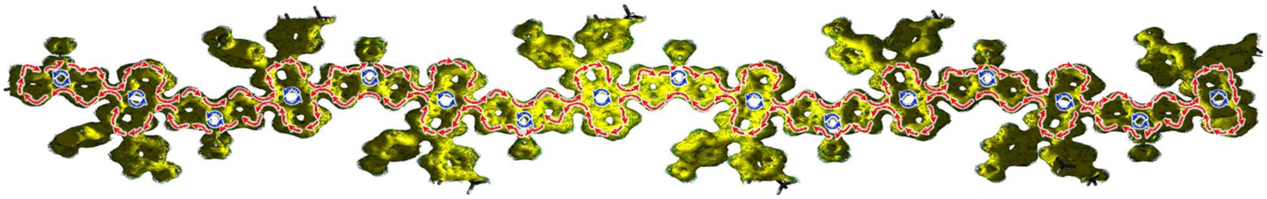

Figure S39. Anisotropy of the induced current density (ACID) of the π-system at CSGT-UCAMB3LYP/6-31G** level of theory for the octamer unit (n = 8). The current density vectors plotted on the ACID isosurface indicate ring current (clockwise, counterclockwise) and prevalent delocalization pathways present in these systems.

5. Supporting Tables

Table S1. Selected electronic properties of oligomers as a function of the number of repeat units.

n^a	$\|\Delta E_{ST}\|^b$ *(eV)*	nHONO^c	nLUNO^c	y^d	$S_{degen}{}^e$	$T_{degen}{}^e$	*SOS-SCS (eV)*	$\mu_g{}^f$ *(Debye)*
**1**	0.769	2	0	0	0	0.527	0	4.06
**2**	0.352	2	0	0	0	0.509	0	6.04
**4**	0.133	1.74174	0.25826	0.043	0.025	0.145	0.007	10.28
**6**	0.072	1.39381	0.60619	0.318	0.034	0.069	0.05	13.55
**8**	0.05	1.21558	0.78442	0.588	0.038	0.040	0.102	15.71

^{a}Number of repeat units (n) for the calculated oligomers. ^{b}Singlet^{BS}-triplet energy gap. ^{c}Natural orbital occupancies, ^{d}diradical character index (y) calculated from Yamaguchi's formula. ^{e}Degeneracy of the singlet and triplet (energy difference between the two NBO e^-s) computed for the diradical (y) formation and ^{f}ground state dipole moment as determined at the UB3LYP/6-31G** level of theory. Natural orbital occupancies (HONO, LUNO) and y are unitless quantities. SCS: singlet closed shell; SOS: singlet open-shell.

Table S2. Bonding orbital interactions between C-Si-C bonds obtained from NBO analysis.

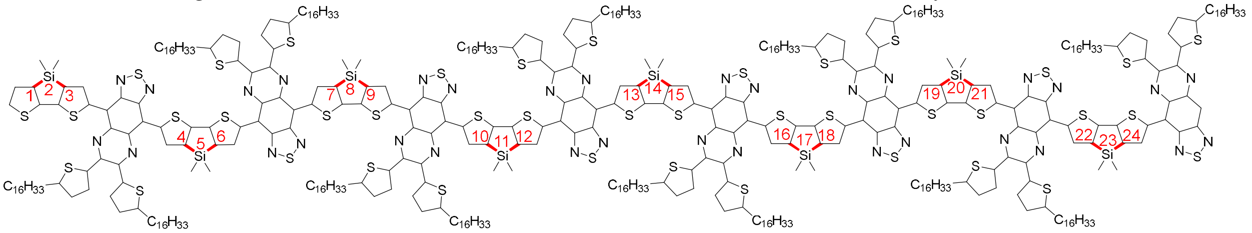

Atoms (Bonding orbitals)		*Percentage contribution*	*Coefficient*	*Hybridization*	
C_1-Si_2	C_1	71.97	0.8483	*s* (31.90%)<br>*p* (68.06%)<br>*d* (0.04%)	$sp^{2.13}$
	Si_2	28.03	0.5295	*s* (23.90%)<br>*p* (75.31%)<br>*d* (0.80%)	$sp^{3.15}d^{0.03}$
Si_2-C_3	Si_2	27.71	0.8502	*s* (32.00%)<br>*p* (67.97%)<br>*d* (0.04%)	$sp^{2.12}$
	C_3	72.29	0.5264	*s* (23.41%)<br>*p* (75.77%)<br>*d* (0.82%)	$sp^{3.24}d^{0.03}$
C_4-Si_5	C_4	72.15	0.8494	*s* (31.94%)<br>*p* (68.02%)<br>*d* (0.04%)	$sp^{2.13}$
	Si_5	27.85	0.5277	*s* (23.59%)<br>*p* (75.61%)<br>*d* (0.80%)	$sp^{3.21}d^{0.03}$
Si_5-C_6	Si_5	27.85	0.5278	*s* (23.56%)<br>*p* (75.64%)	$sp^{3.21}d^{0.03}$

				d (0.80%)	
	C_6	72.15	0.8494	*s* (31.98%)<br>*p* (67.99%)<br>*d* (0.04%)	$sp^{2.13}$
C_7-Si_8	C_7	72.16	0.8495	*s* (31.95%)<br>*p* (68.02%)<br>*d* (0.04%)	$sp^{2.13}$
	Si_8	27.84	0.5277	*s* (23.57%)<br>*p* (75.63%)<br>*d* (0.80%)	$sp^{3.21}d^{0.03}$
Si_8-C_9	Si_8	27.84	0.5276	*s* (23.53%)<br>*p* (75.67%)<br>*d* (0.80%)	$sp^{3.22}d^{0.03}$
	C_9	72.16	0.8495	*s* (31.99%)<br>*p* (67.98%)<br>*d* (0.04%)	$sp^{2.13}$
C_{10}-Si_{11}	C_{10}	72.16	0.8494	*s* (31.94%)<br>*p* (68.02%)<br>*d* (0.04%)	$sp^{2.13}$
	Si_{11}	27.84	0.5277	*s* (23.57%)<br>*p* (75.63%)<br>*d* (0.80%)	$sp^{3.21}d^{0.03}$
Si_{11}-C_{12}	Si_{11}	27.83	0.5276	*s* (23.52%)<br>*p* (75.67%)<br>*d* (0.80%)	$sp^{3.22}d^{0.03}$
	C_{12}	72.17	0.8495	*s* (31.99%)<br>*p* (67.97%)<br>*d* (0.04%)	$sp^{2.12}$
C_{13}-Si_{14}	C_{13}	72.15	0.8494	*s* (31.94%)<br>*p* (68.02%)<br>*d* (0.04%)	$sp^{2.13}$
	Si_{14}	27.85	0.5277	*s* (23.58%)<br>*p* (75.62%)<br>*d* (0.80%)	$sp^{3.21}d^{0.03}$
Si_{14}-C_{15}	Si_{14}	27.83	0.5276	*s* (23.52%)<br>*p* (75.67%)<br>*d* (0.80%)	$sp^{3.22}d^{0.03}$
	C_{15}	72.17	0.8495	*s* (31.99%)<br>*p* (67.97%)<br>*d* (0.04%)	$sp^{2.12}$
C_{16}-Si_{17}	C_{16}	72.15	0.8494	*s* (31.94%)<br>*p* (68.02%)<br>*d* (0.04%)	$sp^{2.13}$
	Si_{17}	27.85	0.5277	*s* (23.58%)<br>*p* (75.62%)<br>*d* (0.80%)	$sp^{3.21}d^{0.03}$
Si_{17}-C_{18}	Si_{17}	27.83	0.5276	*s* (23.52%)<br>*p* (75.67%)<br>*d* (0.80%)	$sp^{3.22}d^{0.03}$
	C_{18}	72.17	0.8495	*s* (31.99%)<br>*p* (67.97%)<br>*d* (0.04%)	$sp^{2.12}$
C_{19}-Si_{20}	C_{19}	72.15	0.8494	*s* (31.94%)<br>*p* (68.03%)<br>*d* (0.04%)	$sp^{2.13}$
	Si_{20}	27.85	0.5277	*s* (23.58%)	$sp^{3.21}d^{0.03}$

				p (75.62%)<br>*d* (0.80%)	
Si₂₀-C₂₁	Si_{20}	27.84	0.5276	*s* (23.53%)<br>*p* (75.67%)<br>*d* (0.80%)	$sp^{3.22}d^{0.03}$
	C_{21}	72.16	0.8495	*s* (32.00%)<br>*p* (67.96%)<br>*d* (0.04%)	$sp^{2.12}$
C_{22}-Si_{23}	C_{22}	72.16	0.8494	*s* (31.92%)<br>*p* (68.04%)<br>*d* (0.04%)	$sp^{2.13}$
	Si_{23}	27.84	0.5277	*s* (23.58%)<br>*p* (75.62%)<br>*d* (0.80%)	$sp^{3.21}d^{0.03}$
Si_{23}-C_{24}	Si_{23}	27.84	0.5277	*s* (23.56%)<br>*p* (75.63%)<br>*d* (0.80%)	$sp^{3.21}d^{0.03}$
	C_{24}	72.16	0.8494	*s* (32.03%)<br>*p* (67.93%)<br>*d* (0.04%)	$sp^{2.12}$

Table S3. Anti-bonding orbital interactions between C-Si-C bonds obtained from NBO analysis.

Atoms (Anti-bonding orbitals)		***Percentage contribution***	***Coefficient***	***Hybridization***	
C_1-Si_2	C_1	28.03	0.5295	*s* (31.90%)<br>*p* (68.06%)<br>*d* (0.04%)	$sp^{2.13}$
	Si_2	71.97	-0.8483	*s* (23.90%)<br>*p* (75.31%)<br>*d* (0.80%)	$sp^{3.15}d^{0.03}$
Si_2-C_3	Si_2	27.71	0.5264	*s* (32.00%)<br>*p* (67.97%)<br>*d* (0.04%)	$sp^{2.12}$
	C_3	72.29	-0.8502	*s* (23.41%)<br>*p* (75.77%)<br>*d* (0.82%)	$sp^{3.24}d^{0.03}$
C_4-Si_5	C_4	27.85	0.5277	*s* (31.94%)<br>*p* (68.02%)<br>*d* (0.04%)	$sp^{2.13}$
	Si_5	72.15	-0.8494	*s* (23.59%)<br>*p* (75.61%)<br>*d* (0.80%)	$sp^{3.21}d^{0.03}$
Si_5-C_6	Si_5	72.15	0.8494	*s* (23.56%)<br>*p* (75.64%)<br>*d* (0.80%)	$sp^{3.21}d^{0.03}$
	C_6	27.85	-0.5278	*s* (31.98%)<br>*p* (67.99%)<br>*d* (0.04%)	$sp^{2.13}$
C_7-Si_8	C_7	27.84	0.5277	*s* (31.95%)<br>*p* (68.02%)<br>*d* (0.04%)	$sp^{2.13}$
	Si_8	72.16	-0.8495	*s* (23.57%)<br>*p* (75.63%)	$sp^{3.21}d^{0.03}$

				d (0.80%)	
Si₈-C₉	Si_8	72.16	0.8495	*s* (23.53%)<br>*p* (75.67%)<br>*d* (0.80%)	$sp^{3.22}d^{0.03}$
	C_9	27.84	-0.5276	*s* (31.99%)<br>*p* (67.98%)<br>*d* (0.04%)	$sp^{2.13}$
C_{10}-Si_{11}	C_{10}	27.84	0.5277	*s* (31.94%)<br>*p* (68.02%)<br>*d* (0.04%)	$sp^{2.13}$
	Si_{11}	72.16	-0.8494	*s* (23.57%)<br>*p* (75.63%)<br>*d* (0.80%)	$sp^{3.21}d^{0.03}$
Si_{11}-C_{12}	Si_{11}	72.17	0.8495	*s* (23.52%)<br>*p* (75.67%)<br>*d* (0.80%)	$sp^{3.22}d^{0.03}$
	C_{12}	27.83	-0.5276	*s* (31.99%)<br>*p* (67.97%)<br>*d* (0.04%)	$sp^{2.12}$
C_{13}-Si_{14}	C_{13}	27.85	0.5277	*s* (31.94%)<br>*p* (68.02%)<br>*d* (0.04%)	$sp^{2.13}$
	Si_{14}	72.15	-0.8494	*s* (23.58%)<br>*p* (75.62%)<br>*d* (0.80%)	$sp^{3.21}d^{0.03}$
Si_{14}-C_{15}	Si_{14}	72.17	0.8495	*s* (23.52%)<br>*p* (75.67%)<br>*d* (0.80%)	$sp^{3.22}d^{0.03}$
	C_{15}	27.83	-0.5276	*s* (31.99%)<br>*p* (67.97%)<br>*d* (0.04%)	$sp^{2.12}$
C_{16}-Si_{17}	C_{16}	27.85	0.5277	*s* (31.94%)<br>*p* (68.02%)<br>*d* (0.04%)	$sp^{2.13}$
	Si_{17}	72.15	-0.8494	*s* (23.58%)<br>*p* (75.62%)<br>*d* (0.80%)	$sp^{3.21}d^{0.03}$
Si_{17}-C_{18}	Si_{17}	72.17	0.8495	*s* (23.52%)<br>*p* (75.67%)<br>*d* (0.80%)	$sp^{3.22}d^{0.03}$
	C_{18}	27.83	-0.5276	*s* (31.99%)<br>*p* (67.97%)<br>*d* (0.04%)	$sp^{2.12}$
C_{19}-Si_{20}	C_{19}	27.85	0.5277	*s* (31.94%)<br>*p* (68.03%)<br>*d* (0.04%)	$sp^{2.13}$
	Si_{20}	72.15	-0.8494	*s* (23.58%)<br>*p* (75.62%)<br>*d* (0.80%)	$sp^{3.21}d^{0.03}$
Si_{20}-C_{21}	Si_{20}	72.16	0.8495	*s* (23.53%)<br>*p* (75.67%)<br>*d* (0.80%)	$sp^{3.22}d^{0.03}$
	C_{21}	27.84	-0.5276	*s* (32.00%)<br>*p* (67.96%)<br>*d* (0.04%)	$sp^{2.12}$
C_{22}-Si_{23}	C_{22}	27.84	0.5277	*s* (31.92%)	$sp^{2.13}$

				p (68.04%)<br>*d* (0.04%)	
	Si_{23}	72.16	-0.8494	*s* (23.58%)<br>*p* (75.62%)<br>*d* (0.80%)	$sp^{3.21}d^{0.03}$
Si_{23}-C_{24}	Si_{23}	72.16	0.8494	*s* (23.56%)<br>*p* (75.63%)<br>*d* (0.80%)	$sp^{3.21}d^{0.03}$
	C_{24}	27.84	-0.5277	*s* (32.03%)<br>*p* (67.93%)<br>*d* (0.04%)	$sp^{2.12}$

Table S4. Tabulated NICS values of the single repeat unit (*n* = 1).

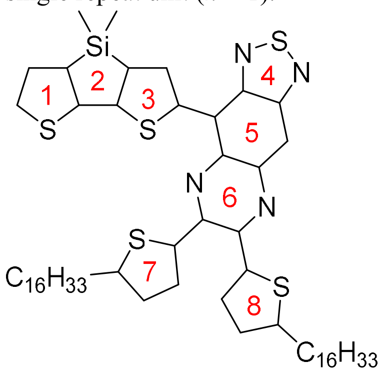

Ring index	Singlet
**1**	-9.87
**2**	+0.89
**3**	-7.90
**4**	-15.28
**5**	-9.63
**6**	-6.76
**7**	-9.33
**8**	-9.00

Table S5. Tabulated NICS values of the dimer (*n* = 2).

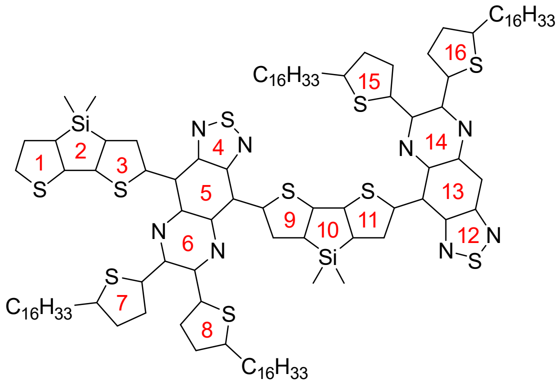

Ring index	Singlet	Ring index	Singlet
**1**	-9.79	**9**	-7.80
**2**	+1.00	**10**	+0.92
**3**	-7.90	**11**	-7.63
**4**	-14.90	**12**	-15.52
**5**	-8.18	**13**	-9.42
**6**	-7.16	**14**	-6.79
**7**	-8.91	**15**	-9.30
**8**	-9.09	**16**	-8.95

Table S6. Tabulated NICS values of the tetramer (n = 4).

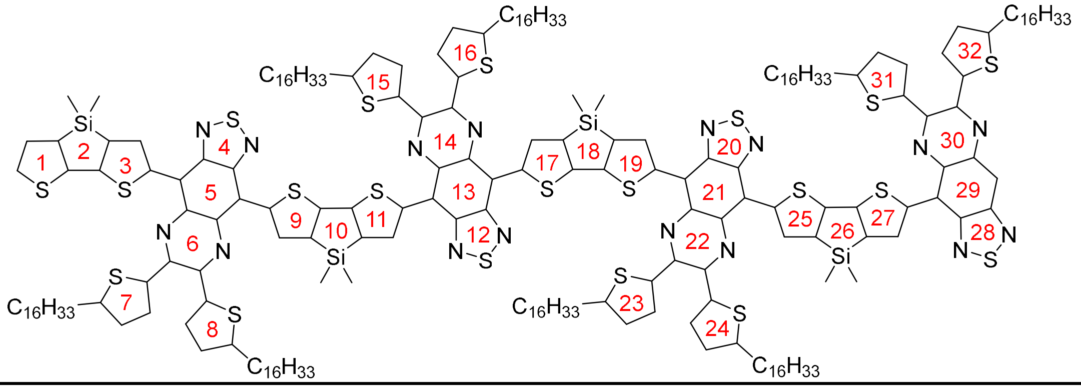

Ring index	Singlet	Ring index	Singlet
**1**	-9.84	**17**	-7.83
**2**	+1.14	**18**	+0.85
**3**	-7.75	**19**	-7.55
**4**	-15.17	**20**	-14.60
**5**	-7.79	**21**	-7.50
**6**	-7.20	**22**	-7.32
**7**	-8.91	**23**	-8.75
**8**	-8.95	**24**	-8.86
**9**	-7.63	**25**	-7.78
**10**	+0.88	**26**	+0.83
**11**	-7.52	**27**	-7.64
**12**	-14.65	**28**	-15.15
**13**	-7.18	**29**	-9.35
**14**	-6.97	**30**	-6.66
**15**	-8.65	**31**	-9.32
**16**	-8.92	**32**	-8.82

Table S7. Tabulated NICS values of the hexamer (n = 6).

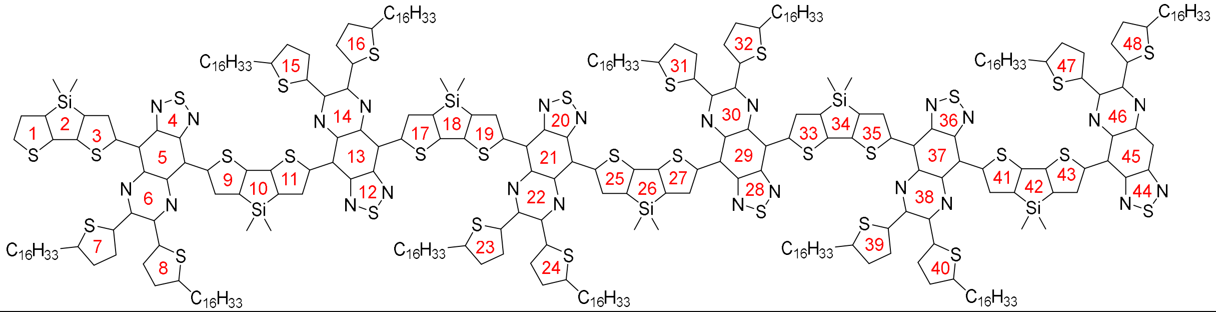

Ring index	Singlet	Ring index	Singlet	Ring index	Singlet
**1**	-9.95	**17**	-7.66	**33**	-7.71
**2**	+1.24	**18**	+0.68	**34**	+0.70
**3**	-7.88	**19**	-7.37	**35**	-7.39
**4**	-15.04	**20**	-14.26	**36**	-14.44
**5**	-7.65	**21**	-6.66	**37**	-7.21
**6**	-7.30	**22**	-7.50	**38**	-7.34
**7**	-8.84	**23**	-8.76	**39**	-8.68
**8**	-8.93	**24**	-9.06	**40**	-8.94
**9**	-7.51	**25**	-7.37	**41**	-7.70
**10**	+0.91	**26**	+0.66	**42**	+0.77
**11**	-7.46	**27**	-7.30	**43**	-7.60
**12**	-14.52	**28**	-14.12	**44**	-15.05
**13**	-6.80	**29**	-6.67	**45**	-9.29
**14**	-7.18	**30**	-7.22	**46**	-6.83
**15**	-8.49	**31**	-8.85	**47**	-8.95
**16**	-8.98	**32**	-8.85	**48**	-9.20

Table S8. Tabulated NICS values of the octamer (n = 8).

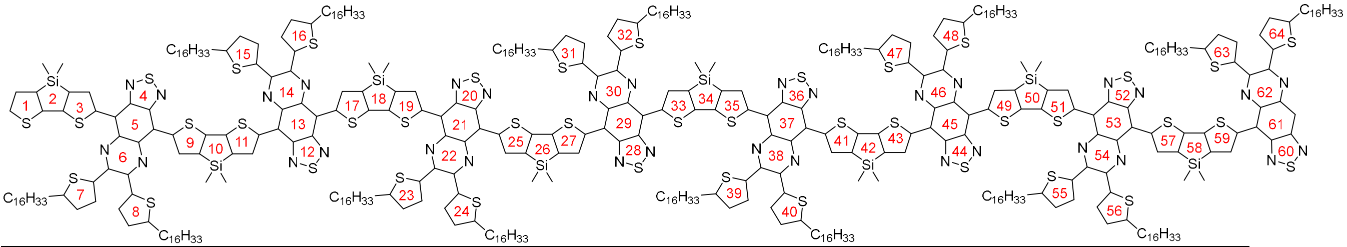

Ring index	Triplet	Ring index	Triplet	Ring index	Triplet	Ring index	Triplet
**1**	-9.35	**17**	-6.54	**33**	-5.92	**49**	-6.76
**2**	+0.55	**18**	-0.18	**34**	-0.55	**50**	+0.03
**3**	-7.15	**19**	-6.35	**35**	-5.95	**51**	-6.87
**4**	-11.77	**20**	-11.67	**36**	-11.97	**52**	-12.92
**5**	-4.36	**21**	-1.54	**37**	-1.38	**53**	-3.35

**6**	-6.88	**22**	-7.34	**38**	-7.35	**54**	-6.98
**7**	-8.60	**23**	-8.95	**39**	-8.92	**55**	-8.83
**8**	-8.48	**24**	-8.51	**40**	-8.63	**56**	-9.28
**9**	-6.73	**25**	-5.83	**41**	-6.20	**57**	-7.04
**10**	+0.03	**26**	-0.51	**42**	-0.20	**58**	+0.71
**11**	-6.45	**27**	-5.81	**43**	-6.27	**59**	-7.24
**12**	-12.04	**28**	-10.57	**44**	-12.22	**60**	-14.52
**13**	-2.42	**29**	-0.78	**45**	-2.12	**61**	-7.85
**14**	-7.04	**30**	-7.09	**46**	-7.04	**62**	-6.50
**15**	-8.96	**31**	-8.86	**47**	-9.02	**63**	-8.81
**16**	-8.93	**32**	-9.07	**48**	-8.99	**64**	-9.33

Table S9. Tabulated bond length values (Å) of the single repeat unit (n = 1).

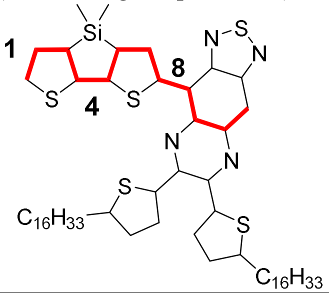

Bond Index	Bond Length (Å)	Bond Index	Bond Length (Å)
**1**	1.372	**7**	1.397
**2**	1.424	**8**	1.450
**3**	1.392	**9**	1.421
**4**	1.449	**10**	1.452
**5**	1.395	**11**	1.386
**6**	1.408		

Table S10. Tabulated bond length values (Å) of the dimer (n = 2).

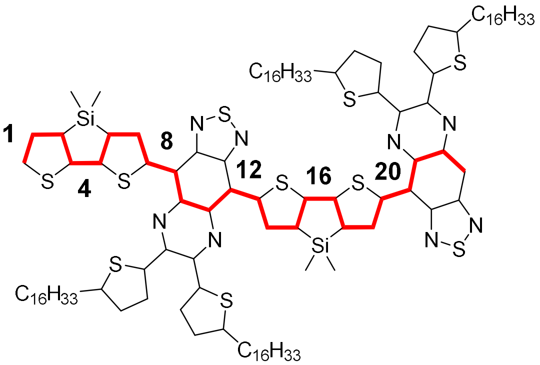

Bond Index	Bond Length (Å)	Bond Index	Bond Length (Å)

**1**	1.372	**13**	1.401
**2**	1.424	**14**	1.403
**3**	1.393	**15**	1.401
**4**	1.448	**16**	1.439
**5**	1.396	**17**	1.401
**6**	1.407	**18**	1.404
**7**	1.399	**19**	1.401
**8**	1.447	**20**	1.447
**9**	1.421	**21**	1.423
**10**	1.453	**22**	1.452
**11**	1.426	**23**	1.387
**12**	1.448		

Table S11. Tabulated bond length values (Å) of the tetramer (n = 4).

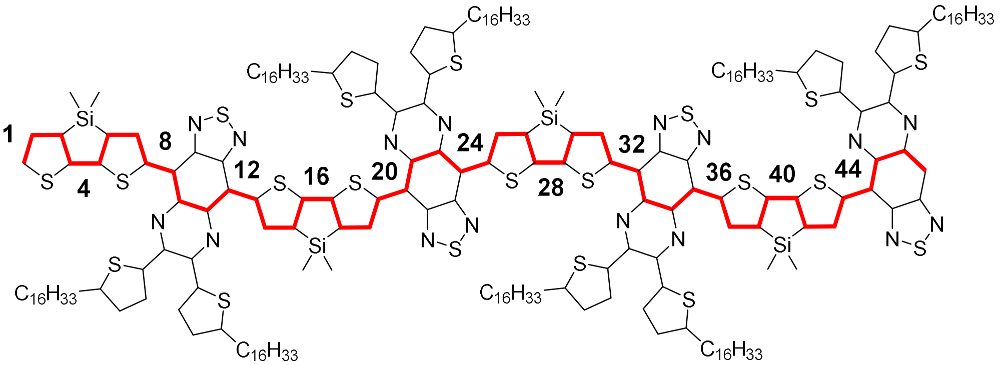

Bond Index	Bond Length (Å)	Bond Index	Bond Length (Å)	Bond Index	Bond Length (Å)	Bond Index	Bond Length (Å)
**1**	1.372	**13**	1.409	**25**	1.412	**37**	1.407
**2**	1.423	**14**	1.396	**26**	1.394	**38**	1.399
**3**	1.394	**15**	1.408	**27**	1.410	**39**	1.404
**4**	1.447	**16**	1.428	**28**	1.426	**40**	1.435
**5**	1.397	**17**	1.409	**29**	1.410	**41**	1.404
**6**	1.406	**18**	1.395	**30**	1.395	**42**	1.401
**7**	1.401	**19**	1.411	**31**	1.411	**43**	1.403
**8**	1.444	**20**	1.434	**32**	1.435	**44**	1.445
**9**	1.426	**21**	1.432	**33**	1.431	**45**	1.424
**10**	1.452	**22**	1.449	**34**	1.450	**46**	1.451
**11**	1.434	**23**	1.439	**35**	1.435	**47**	1.388
**12**	1.440	**24**	1.435	**36**	1.441		

Table S12. Tabulated bond length values (Å) of the hexamer (n = 6).

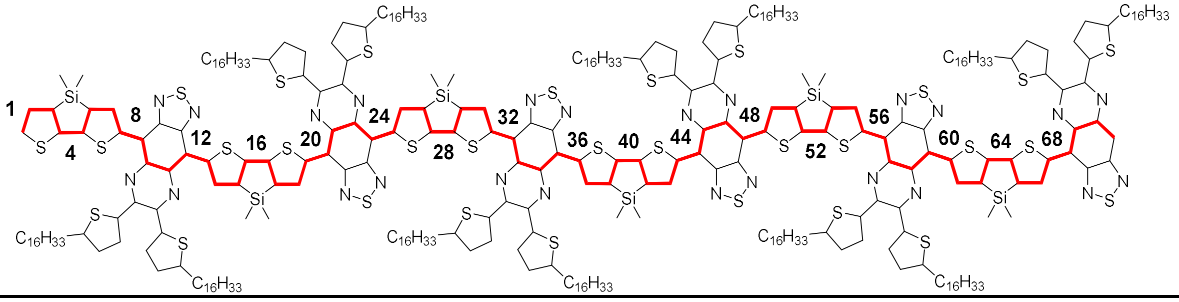

Bond Index	Bond Length (Å)	Bond Index	Bond Length (Å)	Bond Index	Bond Length (Å)
**1**	1.373	**25**	1.419	**49**	1.417
**2**	1.423	**26**	1.388	**50**	1.389
**3**	1.393	**27**	1.417	**51**	1.415
**4**	1.446	**28**	1.417	**52**	1.421
**5**	1.398	**29**	1.418	**53**	1.414
**6**	1.405	**30**	1.388	**54**	1.391
**7**	1.402	**31**	1.420	**55**	1.415
**8**	1.442	**32**	1.424	**56**	1.430
**9**	1.428	**33**	1.440	**57**	1.434
**10**	1.451	**34**	1.446	**58**	1.449
**11**	1.438	**35**	1.448	**59**	1.438
**12**	1.436	**36**	1.425	**60**	1.438
**13**	1.412	**37**	1.420	**61**	1.409
**14**	1.393	**38**	1.387	**62**	1.397
**15**	1.412	**39**	1.418	**63**	1.406
**16**	1.424	**40**	1.416	**64**	1.433
**17**	1.413	**41**	1.418	**65**	1.405
**18**	1.392	**42**	1.388	**66**	1.401
**19**	1.415	**43**	1.419	**67**	1.404
**20**	1.428	**44**	1.424	**68**	1.445
**21**	1.437	**45**	1.439	**69**	1.425
**22**	1.447	**46**	1.446	**70**	1.451
**23**	1.446	**47**	1.446	**71**	1.388
**24**	1.427	**48**	1.428		

Table S13. Tabulated bond length values (Å) of the octamer (n = 8).

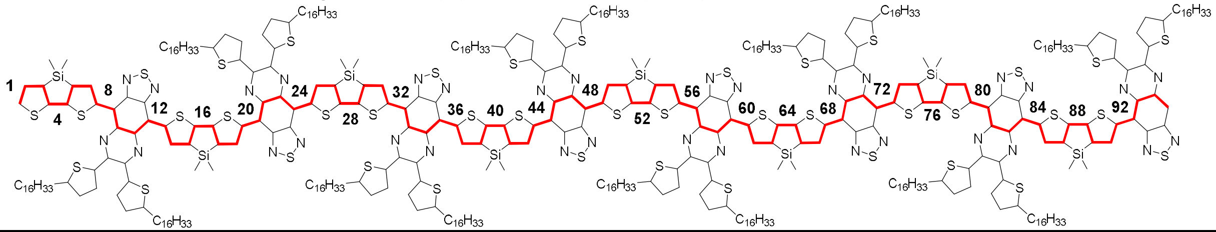

Bond Index	Bond Length (Å)	Bond Index	Bond Length (Å)	Bond Index	Bond Length (Å)
**1**	1.373	**33**	1.448	**65**	1.427
**2**	1.423	**34**	1.443	**66**	1.380
**3**	1.394	**35**	1.459	**67**	1.428
**4**	1.446	**36**	1.411	**68**	1.415
**5**	1.398	**37**	1.433	**69**	1.445
**6**	1.404	**38**	1.375	**70**	1.444
**7**	1.403	**39**	1.432	**71**	1.452
**8**	1.441	**40**	1.397	**72**	1.422
**9**	1.429	**41**	1.433	**73**	1.422
**10**	1.450	**42**	1.374	**74**	1.385
**11**	1.440	**43**	1.435	**75**	1.419
**12**	1.433	**44**	1.406	**76**	1.415
**13**	1.415	**45**	1.452	**77**	1.418
**14**	1.391	**46**	1.441	**78**	1.388
**15**	1.414	**47**	1.462	**79**	1.419
**16**	1.419	**48**	1.407	**80**	1.426
**17**	1.416	**49**	1.436	**81**	1.437
**18**	1.389	**50**	1.373	**82**	1.447
**19**	1.419	**51**	1.434	**83**	1.441
**20**	1.424	**52**	1.395	**84**	1.436
**21**	1.441	**53**	1.434	**85**	1.411
**22**	1.445	**54**	1.374	**86**	1.396
**23**	1.451	**55**	1.435	**87**	1.407
**24**	1.420	**56**	1.407	**88**	1.432
**25**	1.425	**57**	1.451	**89**	1.406
**26**	1.381	**58**	1.442	**90**	1.400
**27**	1.424	**59**	1.459	**91**	1.404
**28**	1.407	**60**	1.413	**92**	1.444
**29**	1.426	**61**	1.430	**93**	1.425
**30**	1.381	**62**	1.378	**94**	1.451
**31**	1.428	**63**	1.428	**95**	1.388
**32**	1.413	**64**	1.403		

Table S14. Measurement conditions for the materials reported in **Figure S16**.

Material	***Frequency***	***Sample State***
Fe_8	240 GHz	Single crystal
Cr_7Ni	X band	0.2 mg mL^{-1} solution in toluene
CpTi(cot)	X band	3% in solid- state isostructural diamagnetic matrix
$Ni(phen)_3$	W band	1 mM solution in 1:1 H_2O Glycerol

VO(dmit)₃	X band	5% in solid- state isostructural diamagnetic matrix
CuPc	Q band	0.1% in solid- state isostructural diamagnetic matrix
N@C$_{60}$	X band	1 μM solution in CS_2
NV Center	240 GHz	Solid- state impurity density 10^{17}-10^{18} cm^{-3}
TTBrM	X band	0.01 w/w in PMMA
PTM	Q band	0.1% in PSF
TM	X band	1:1000 in solid- state diamagnetic matrix
TEMPO SAM	Q band	0.2 × 1 cm strip
Graphenoid	X band	Powder
GNR	X Band	Powder
DPPT-TT	X Band	FET device on fused-quartz
PT	X Band	Film with 1% doping on quartz
IDTBT	X Band	FET device on quartz
NDI	X Band	FET device on quartz
SiC	1.35 GHz	PL6 divacancy in 12.5 μM single crystal 4H-SiC with N dopant density of 5 × 10^{15} cm^{-3}

6. Supporting References

[1] K. S. Mayer, D. J. Adams, N. Eedugurala, et al., Topology and ground state control in open-shell donor-acceptor conjugated polymers, *Cell Rep. Phys. Sci.* **2021**, 2, 100467.

[2] L. Huang, N. Eedugurala, A. Benasco, et al., Open-Shell Donor–Acceptor Conjugated Polymers with High Electrical Conductivity, *Adv. Funct. Mater.* **2020**, 30, 1909805.

[3] N. Eedugurala, M. E. Steelman, P. Mahalingavelar, et al., Strong Acceptor Annulation Enables Control of Electronic Structure and Spin Configuration in Donor–Acceptor Conjugated Polymers, *Chem. Mater.* **2023**, 35, 3115.

[4] S. Stoll, A. Schweiger, EasySpin, a comprehensive software package for spectral simulation and analysis in EPR, *J. Magn. Reson.* **2006**, 178, 42.

[5] M. Brustolon, E. Giamello, *Electron Paramagnetic Resonance: A Practitioner's Toolkit*, John Wiley & Sons, **2009**.

[6] D. Goldfarb, S. Stoll, *EPR Spectrscopy: Fundamentals and Methods*, John Wiley & Sons, **2018**.

[7] J. R. Klauder, P. W. Anderson, Spectral Diffusion Decay in Spin Resonance Experiments, *Phys. Rev.* **1962**, 125, 912.

[8] S. Mugiraneza, A. M. Hallas, Tutorial: a beginner's guide to interpreting magnetic susceptibility data with the Curie-Weiss law, *Commun. Phys.* **2022**, 5, 95.

[9] D. J. Adams, K. S. Mayer, M. Steelman, J. D. Azoulay, Magnetic Characterization of Open-Shell Donor–Acceptor Conjugated Polymers, *J. Phys. Chem. C* **2022**, 126, 5701.

[10] A. Rajca, Organic Diradicals and Polyradicals From Spin Coupling to Magnetism, *Chem. Rev.* **1994**, 94, 871.

[11] M. E. Steelman, D. J. Adams, K. S. Mayer, et al., Magnetic Ordering in a High-Spin Donor-Acceptor Conjugated Polymer, *Adv. Mater.* **2022**, 34, 2206161.

[12] F. Lombardi, A. Lodi, J. Ma, et al., Quantum units from the topological engineering of molecular graphenoids, *Science* **2019**, 366, 1107.

[13] M. J. Frisch, G. W. Trucks, H. B. Schlegel, et al., Gaussian 16 Rev. C.01., **2016**.

[14] A. D. Becke, A new mixing of Hartree–Fock and local density-functional theories *J. Chem. Phys.* **1993**, 98, 1372.

[15] M. M. Francl, W. J. Pietro, W. J. Hehre, et al., Self-consistent molecular orbital methods. XXIII. A polarization-type basis set for second-row elements, *J. Chem. Phys.* **1982**, 77, 3654.

[16] S. Yamanaka, T. Kawamura, T. Noroa, K. Yamaguchi, Heisenberg model for radical reactions. Part 3. Direct exchange coupling between transition metal ions and triplet methylene, *J. Mol. Struct.* **1994**, 310, 185.

[17] S. Takahashi, I. S. Tupitsyn, J. van Tol, C. C. Beedle, D. N. Hendrickson, P. C. Stamp, Decoherence in crystals of quantum molecular magnets, *Nature* **2011**, 476, 76.

[18] A. Ardavan, O. Rival, J. J. Morton, et al., Will spin-relaxation times in molecular magnets permit quantum information processing?, *Phys. Rev. Lett.* **2007**, 98, 057201.

[19] L. C. de Camargo, M. Briganti, F. S. Santana, et al., Exploring the Organometallic Route to Molecular Spin Qubits: The [CpTi(cot)] Case, *Angew. Chem. Int.* **2021**, 60, 2588.

[20] M. K. Wojnar, D. W. Laorenza, R. D. Schaller, D. E. Freedman, Nickel(II) Metal Complexes as Optically Addressable Qubit Candidates, *J. Am. Chem. Soc.* **2020**, 142, 14826.

[21] M. Atzori, E. Morra, L. Tesi, et al., Quantum Coherence Times Enhancement in Vanadium(IV)-based Potential Molecular Qubits: the Key Role of the Vanadyl Moiety, *J. Am. Chem. Soc.* **2016**, 138, 11234.

[22] M. Warner, S. Din, I. S. Tupitsyn, et al., Potential for spin-based information processing in a thin-film molecular semiconductor, *Nature* **2013**, 503, 504.

[23] J. J. Morton, A. M. Tyryshkin, A. Ardavan, K. Porfyrakis, S. A. Lyon, G. A. D. Briggs, Electron spin relaxation of N@C60 in CS2 in CS2, *J. Chem. Phys.* **2006**, 124, 14508.

[24] S. Takahashi, R. Hanson, J. van Tol, M. S. Sherwin, D. D. Awschalom, Quenching spin decoherence in diamond through spin bath polarization, *Phys. Rev. Lett.* **2008**, 101, 047601.

[25] Y.-S. Zhang, Y.-F. Fan, X.-Q. Tao, et al., Potential molecular qubits with long coherence time constructed using bromo-substituted trityl radicals, *J. Mater. Chem. C* **2024**, 12, 5150.

[26] D. Schafter, J. Wischnat, L. Tesi, et al., Molecular One- and Two-Qubit Systems with Very Long Coherence Times, *Adv. Mater.* **2023**, 35, 2302114.

[27] Y. Z. Dai, B. W. Dong, Y. Kao, et al., Chemical Modification toward Long Spin Lifetimes in Organic Conjugated Radicals, *ChemPhysChem* **2018**, 19, 2972.

[28] L. Tesi, F. Stemmler, M. Winkler, et al., Modular Approach to Creating Functionalized Surface Arrays of Molecular Qubits, *Adv. Mater.* **2023**, 35, 2208998.

[29] M. Slota, A. Keerthi, W. K. Myers, et al., Magnetic edge states and coherent manipulation of graphene nanoribbons, *Nature* **2018**, 557, 691.

[30] R. L. Carey, S. Giannini, S. Schott, et al., Spin relaxation of electron and hole polarons in ambipolar conjugated polymers, *Nat. Commun.* **2024**, 15, 288.

[31] M. Scharli, H. Kiess, G. Harbeke, E.S.R. of BF4- Doped Polythiophene, *Synth. Met.* **1988**, 22, 317.

[32] S. Schott, U. Chopra, V. Lemaur, et al., Polaron spin dynamics in high-mobility polymeric semiconductors, *Nat. Phys.* **2019**, 15, 814.

[33] Q. Li, J. F. Wang, F. F. Yan, et al., Room-temperature coherent manipulation of single-spin qubits in silicon carbide with a high readout contrast, *Natl. Sci. Rev.* **2022**, 9, nwab122.
